# Acyl carrier protein is essential for apicoplast biogenesis in malaria parasites independent of fatty acid synthesis

**DOI:** 10.7554/eLife.111494

**Published:** 2026-09-14

**Authors:** Sage WR Geher, Seyi Falekun, Jessica N Pita-Aquino, Russell P Swift, Megan Okada, Yasaman Jami-Alahmadi, James A Wohlschlegel, Sean T Prigge, Paul A Sigala

**Affiliations:** 1 Department of Biochemistry, University of Utah School of Medicine Salt Lake City United States; 2 Department of Molecular Microbiology and Immunology, Johns Hopkins School of Public Health Baltimore United States; 3 Department of Biological Chemistry, University of California, Los Angeles Los Angeles United States; University of Geneva Switzerland; University of Geneva Switzerland

**Keywords:** malaria parasites, apicoplast, metabolic regulation, pyruvate kinase, acyl carrier protein, FASII, *P. falciparum*

## Abstract

Acyl carrier protein (ACP) and its 4-phosphopantetheine prosthetic group canonically function as the soluble scaffold for acyl chain assembly and elongation during type II fatty acid biosynthesis (FASII). *Plasmodium* malaria parasites retain a FASII pathway in the apicoplast organelle that has been the subject of considerable scrutiny and confusion. Although apicoplast FASII is essential for *Plasmodium falciparum* growth within mosquitoes and the human liver, this pathway is dispensable and largely inactive in blood-stage parasites that can scavenge host fatty acids. In contrast to FASII enzymes that can be disrupted without fitness defect, we report that knockout or ligand-dependent knockdown of apicoplast ACP is lethal to blood-stage *P. falciparum*, indicating an essential FASII-independent function. Loss of ACP impairs the biosynthesis of essential isoprenoid precursors and blocks apicoplast biogenesis. Using proximity biotinylation and biochemical interaction studies, we identified a key role for ACP in binding and stabilizing apicoplast pyruvate kinase II (PKII). This critical enzyme is the only known source of nucleoside triphosphates (NTPs) in this organelle and is required for isoprenoid synthesis and apicoplast biogenesis. Our work reveals that ACP knockdown results in destabilization and loss of PKII, which is sufficient to explain ACP essentiality in this stage. This work unveils essential ACP function at a key biochemical hub controlling broad apicoplast metabolism in malaria parasites that is independent of the canonical ACP role in FASII.

## Introduction

Malaria remains a pressing global health threat, with a disproportionate impact on under-resourced communities in Africa that is likely to worsen due to funding cutbacks by key donor countries (Stover et al., 2025). At the same time, the efficacy of current therapies is threatened by ongoing resistance development by virulent *Plasmodium falciparum* parasites (The Lancet Microbe, 2024; White, 2004). These concerns highlight the critical need to deepen understanding of *Plasmodium* biology to optimize the use of current medicines and to develop new therapeutic strategies.

*Plasmodium* parasites retain two critical organelles, the mitochondrion and a non-photosynthetic plastid termed the apicoplast, which carry out critical metabolic functions and are validated drug targets (Dahl et al., 2006; Yeh and DeRisi, 2011; Painter et al., 2007; Fry and Pudney, 1992). Since human cells lack an apicoplast, this organelle and its prokaryotic-like pathways were considered to be fertile ground for discovering new parasite-specific drug targets. However, the identification of essential pathways within the apicoplast has been challenging, since many of its metabolic functions are dispensable during parasite growth within red blood cells (RBCs) and are only required in the mosquito and/or liver stages. For example, biosynthetic pathways in the apicoplast for making acetyl CoA, lipoate, fatty acids, and heme biosynthesis intermediates are all dispensable during blood-stage growth (Sigala et al., 2015; Rizopoulos et al., 2016; Cobbold et al., 2013; Swift et al., 2022; Günther et al., 2007; Shears et al., 2015; van Schaijk et al., 2014; Yu et al., 2008). Indeed, biosynthesis of the isoprenoid precursor, isopentenyl pyrophosphate (IPP), and the final step in the production of coenzyme A are the only known essential apicoplast pathways whose metabolic outputs are required outside the organelle (Yeh and DeRisi, 2011; Swift et al., 2021). However, pathways that support apicoplast maintenance and IPP synthesis (Elahi and Prigge, 2023; Okada and Sigala, 2023), including protein and DNA synthesis (Dahl and Rosenthal, 2007; Uddin et al., 2018; Lindner et al., 2011), RNA transcription (Blackwell et al., 2024; Hollin et al., 2025), Fe-S cluster biogenesis (Swift et al., 2023; Haussig et al., 2014), and (d)NTP production (Swift et al., 2020a), are also essential for parasite viability.

**Figure 1.**
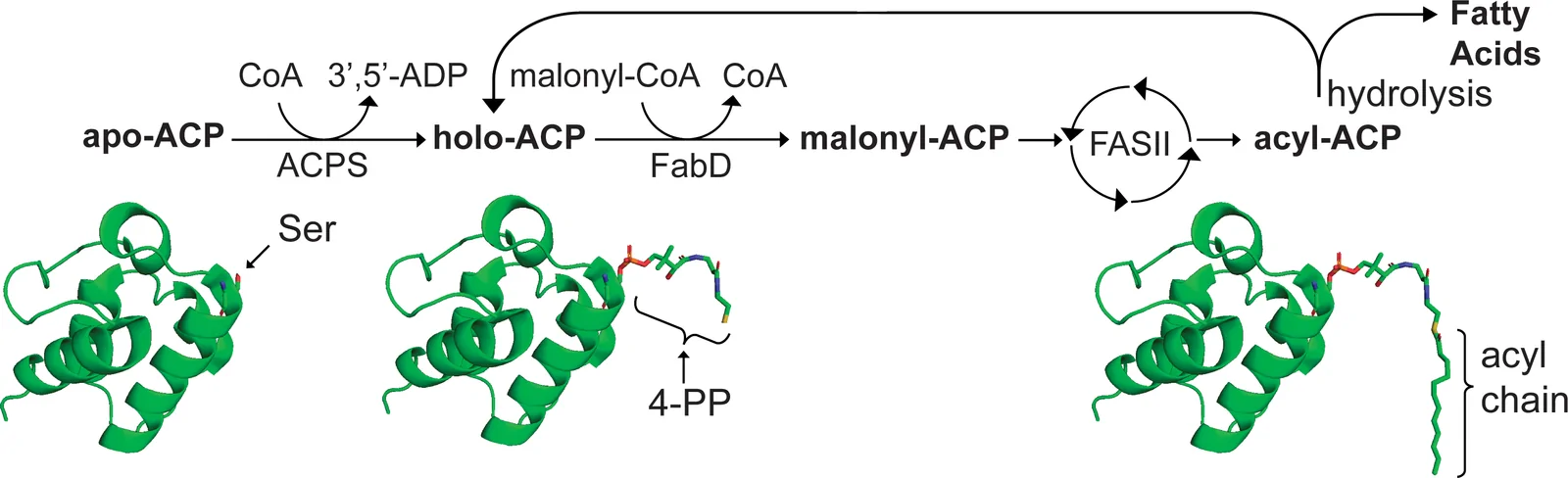
Schematic depiction of apicoplast acyl carrier protein (ACP) and the type II fatty acid biosynthesis (FASII) pathway in *P.* *falciparum*. CoA = coenzyme A, ACPS = holo-ACP synthase, FabD = malonyl-CoA:ACP *S*-malonyltransferase, 4-PP = 4-phosphopantetheine. The ACP structural models are based on the X-ray structures of *P. falciparum* holo-aACP (PDB 3GZM) (Gallagher and Prigge, 2010) and *Escherichia coli* acyl-ACP (PDB 5USR) (Cory et al., 2017).

Fatty acid synthesis in the apicoplast has been the subject of considerable confusion and controversy since the sequencing of the *P. falciparum* genome (Ralph et al., 2004; Gardner et al., 2002; Spalding and Prigge, 2008). The apicoplast encodes a type II fatty acid biosynthesis (FASII) pathway for making octanoic acid used in lipoate biosynthesis and for elongation into longer-chain fatty acids (Ralph et al., 2004). FASII activity involves over seven enzymes of prokaryotic origin for initiation, modification, and elongation of a growing acyl chain tethered to the terminal thiol of a 4-phosphopantetheine (4-PP) group on the acyl carrier protein (ACP, Pf3D7_0208500), which serves as the soluble scaffold for fatty acid synthesis (Figure 1; Chan and Vogel, 2010). This pathway was originally proposed to be essential for blood-stage parasites, based on the anti-*Plasmodium* activity of triclosan, an inhibitor of the FASII enoyl-ACP reductase enzyme (FabI) in bacteria (Surolia and Surolia, 2001). However, subsequent gene-expression and knockout studies of FabI and other FASII enzymes and isotope-tracing experiments clarified that this pathway is dispensable and largely inactive in blood-stage *Plasmodium*, which can scavenge host-derived fatty acids (Shears et al., 2015; van Schaijk et al., 2014; Botté et al., 2013). Indeed, FASII enzymes only appear to be critical for the highly proliferative stages of *P. falciparum* growth in mosquitoes and the human liver (van Schaijk et al., 2014; Yu et al., 2008).

Although FASII activity is dispensable for blood-stage parasites, we noted that genome-wide knockout and insertional mutagenesis studies in *Plasmodium berghei* and *P. falciparum* reported that apicoplast ACP (aACP) could not be disrupted, suggesting an essential function independent of FASII (Zhang et al., 2018; Bushell et al., 2017). ACP is a small, highly α-helical protein that contains a strictly conserved Ser residue modified by the 4-PP group derived from coenzyme A (Figure 1; Beld et al., 2014; Gallagher and Prigge, 2010). The 4-PP group is attached to ACP by the holo-ACP synthase enzyme (Pf3D7_0420200), which is also predicted to be essential for blood-stage *P. falciparum* (Zhang et al., 2018). Organelle targeting and proteolytic processing of ACP have been heavily studied in *P. falciparum* (Waller et al., 2000; van Dooren et al., 2002), but ACP roles apart from FASII have not been identified in the parasite apicoplast or in related plastids like chloroplasts (Hölzl and Dörmann, 2019). However, expanded ACP functions beyond acyl chain synthesis are well known in mitochondria. Indeed, most eukaryotes retain a complete mitochondrial FASII pathway whose ACP also plays a critical role in binding and stabilizing key proteins involved in Fe-S cluster biogenesis and assembly of respiratory chain complexes and (in mammals) of mitochondrial ribosomes (Van Vranken et al., 2018; Masud et al., 2019; Angerer et al., 2017; Brown et al., 2017). In contrast to most eukaryotes, *P. falciparum* and other apicomplexan parasites lack mitochondrial FASII. Nevertheless, they retain an essential ACP homolog (Pf3D7_1208300) in this organelle that uses a divergent molecular interface lacking a 4-PP group to bind and stabilize the Isd11-Nfs1 complex required for mitochondrial Fe-S cluster biogenesis (Falekun et al., 2021).

We set out to test and understand the FASII-independent function of aACP in blood-stage *P. falciparum*. ACP knockout and knockdown studies confirmed an essential role for this protein in apicoplast biogenesis and IPP synthesis. Biochemical interaction studies uncovered a critical stabilizing interaction between aACP and the apicoplast pyruvate kinase II (PKII) enzyme that is essential for (d)NTP and pyruvate synthesis required for broad apicoplast metabolism. This interaction appears to require the 4-PP prosthetic group on aACP, whose modification status may differentially regulate PKII activity and thus apicoplast function in distinct host environments. Our study unveils a new molecular paradigm for essential ACP function at a key metabolic hub in the apicoplast of malaria parasites, independent of the canonical ACP role in FASII.

## Results

### ACP is essential for parasite viability and apicoplast biogenesis

**Figure 2.**
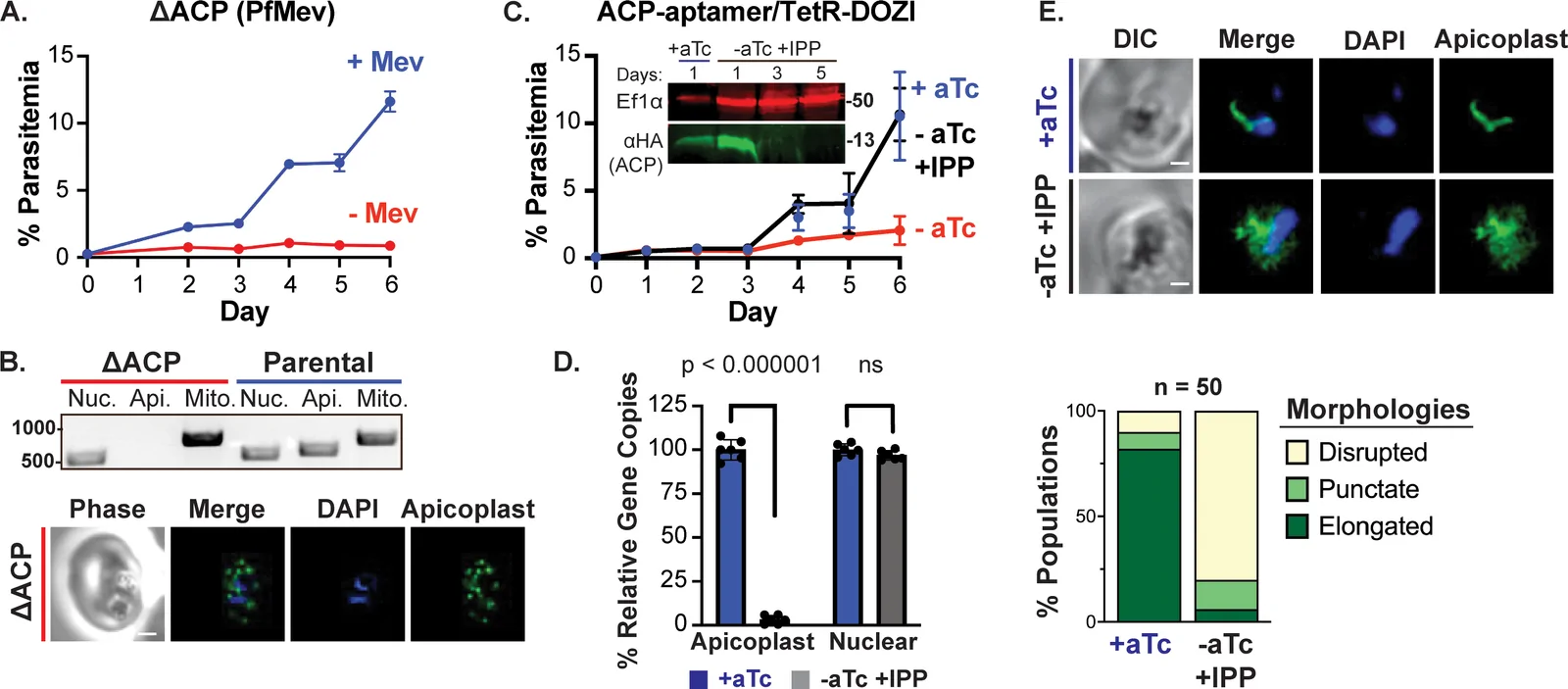
Knockout or knockdown of acyl carrier protein (ACP) expression blocks parasite growth and apicoplast biogenesis. (**A**) Synchronous growth assay of ∆ACP PfMev parasites cultured ±50 µM mevalonate (Mev). Parasitemia values are the average ± SD from biological triplicate samples. (**B**) Genomic PCR analysis and live parasite imaging show selective loss of the apicoplast genome and disrupted apicoplast morphology based on fluorescence of the ACP_L_-GFP marker protein in PfMev parasites. Nuc.=nuclear gene (LDH, Pf3D7_1324900), Api.=apicoplast gene (SufB, Pf3D7_API04700), Mito.=mitochondrial gene (CoxI, Pf3D7_MIT02100). (**C**) Synchronous growth assay of apicoplast ACP-aptamer/TetR-DOZI Dd2 parasites cultured ±1 µM aTc and ±200 µM isopentenyl pyrophosphate (IPP). Parasitemia values are the average ± SD from biological triplicates. Inset: western blot analysis of parasites harvested after 1, 3, or 5 days at the indicated growth conditions and probed with anti-EF1α (cytosolic loading control) or anti-HA (ACP) antibodies. (**D**) Quantitative PCR analysis of the apicoplast:nuclear genome ratio for parasites in panel C cultured 84 hr in the indicated conditions, based on amplification of apicoplast (SufB, ClpM: Pf3D7_API03600, TufA: Pf3D7_API02900) relative to nuclear (STL: Pf3D7_0717700, I5P: Pf3D7_0802500, ADSL: Pf3D7_0206700) genes. Indicated quantitative PCR (qPCR) ratios were normalized to +aTc and are the average ± SD of biological triplicates. Significance was analyzed by unpaired Student’s t-test to determine the indicated p-values. (**E**) Immunofluorescence microscopy of parasites in panel C cultured for 5 days (120 hr) in the indicated conditions and stained with anti-apicoplast ACP or DAPI (nucleus). Below: population analysis of apicoplast morphology scored for disrupted (dispersed), punctate, or elongated GFP signal in 50 total parasites from biological triplicates. Scale bars ~1 µm. Figure 2—source data 1.PDF file containing uncropped gel images for PCR analysis in Figure 2B and western blot analysis in Figure 2C. Figure 2—source data 2.Original files for PCR gel analysis in Figure 2B and western blot analysis in Figure 2C.

Prior studies successfully deleted most FASII enzymes in blood-stage *Plasmodium*, but there have been no reported attempts to disrupt aACP (Shears et al., 2015). To directly test aACP essentiality for blood-stage parasites, we targeted the ACP gene (Pf3D7_0208500) for deletion in PfMev NF54 parasites (Swift et al., 2020b). These parasites express apicoplast-targeted GFP and have been engineered to use exogenous mevalonate to synthesize IPP in the cytoplasm, which bypasses the endogenous apicoplast IPP synthesis pathway and rescues parasites from lethal apicoplast defects. Parasites successfully returned from transfection only in the presence of mevalonate, and genomic PCR analysis confirmed successful deletion of the aACP gene (Figure 2—figure supplement 1). Growth of ∆ACP parasites was strictly dependent on exogenous mevalonate (Figure 2A). Fluorescence microscopy revealed that the apicoplast-targeted GFP signal in ∆ACP parasites displayed a dispersed constellation of GFP foci, suggesting organelle disruption due to a defect in apicoplast biogenesis and inheritance (Figure 2B, Figure 2—figure supplement 2). PCR analysis confirmed selective loss of the apicoplast genome in these parasites (Figure 2B), supporting the conclusion that loss of aACP results in a lethal defect in apicoplast biogenesis and IPP synthesis.

**Figure 2—figure supplement 1.**
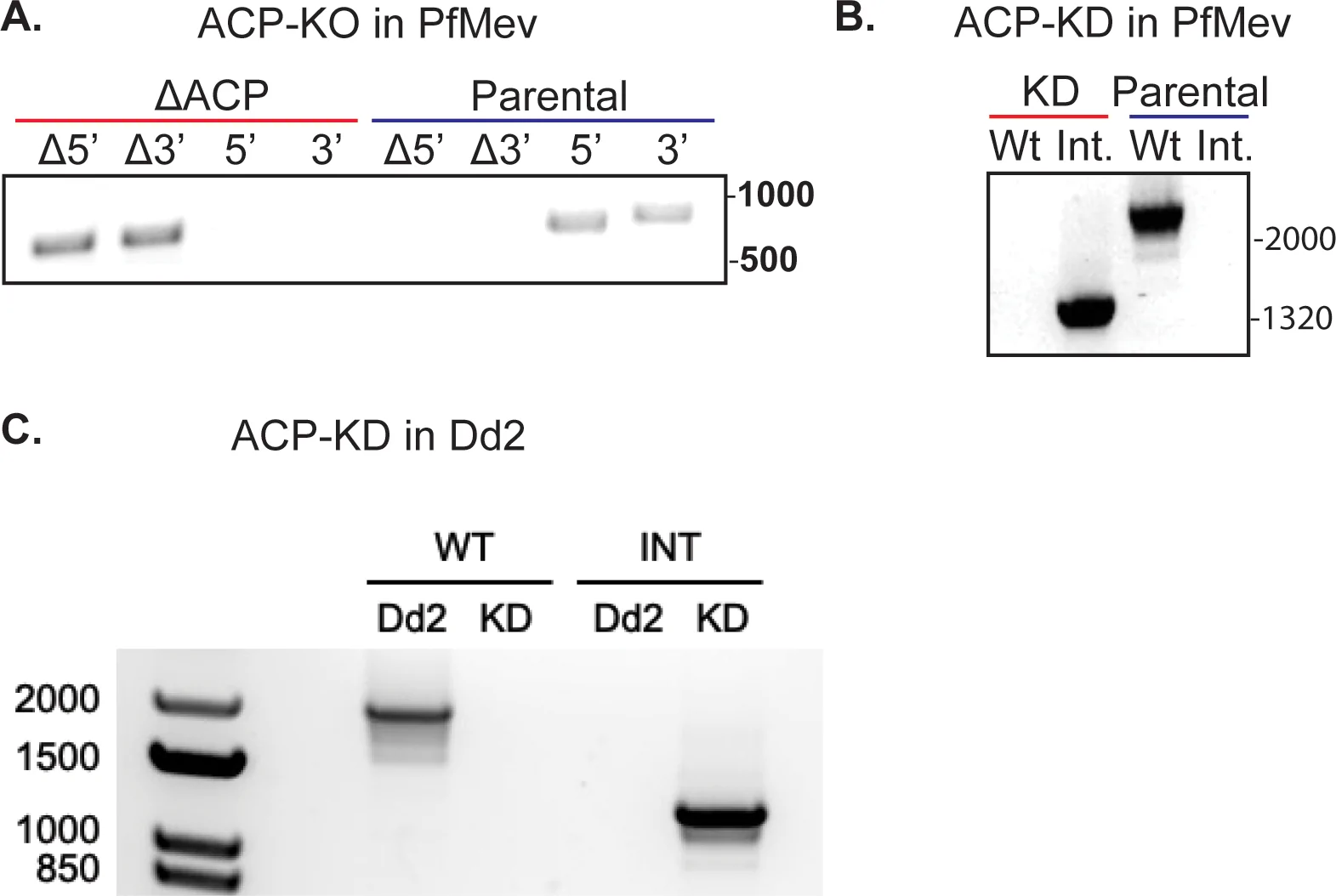
Integration PCRs for deletion or modification of the apicoplast acyl carrier protein (ACP) gene. (**A**) Genotyping PCR for successful deletion of the apicoplast ACP gene (Pf3D7_0208500) in polyclonal PfMev parasites showing selective amplification of sequence at the 5’ and 3’ ends of the drug resistance marker (∆5’ and ∆3’) in knockout parasites but not in parental PfMev parasites and exclusive amplification of sequence at the 5’ and 3’ ends of the ACP CDS in wild-type (WT) but not ∆ACP parasites. Genotype PCR analysis confirming on-target integration of the 3’ aptamer/TetR-DOZI cassette at the apicoplast ACP locus in polyclonal PfMev (**B**) or clonal Dd2 parasites (**C**) and the absence of the unmodified WT locus, with parental parasites serving as negative control for integration. Figure 2—figure supplement 1—source data 1.PDF containing uncropped gel images for integration PCR analyses shown in Figure 2—figure supplement 1. Figure 2—figure supplement 1—source data 2.Original files for PCR gel analyses in Figure 2—figure supplement 1.

**Figure 2—figure supplement 2.**
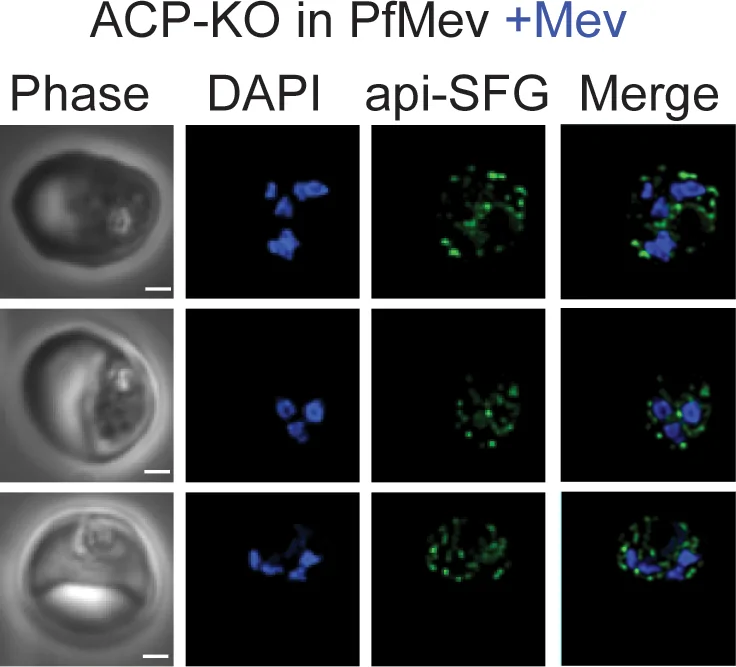
Additional microscopy images of ∆ACP PfMev NF54 parasites. Phase = phase contrast images, api-SFG=ACP_L_-superfolder GFP, DAPI = nuclear DNA stain. Scale bars ~1 µm.

**Figure 2—figure supplement 3.**
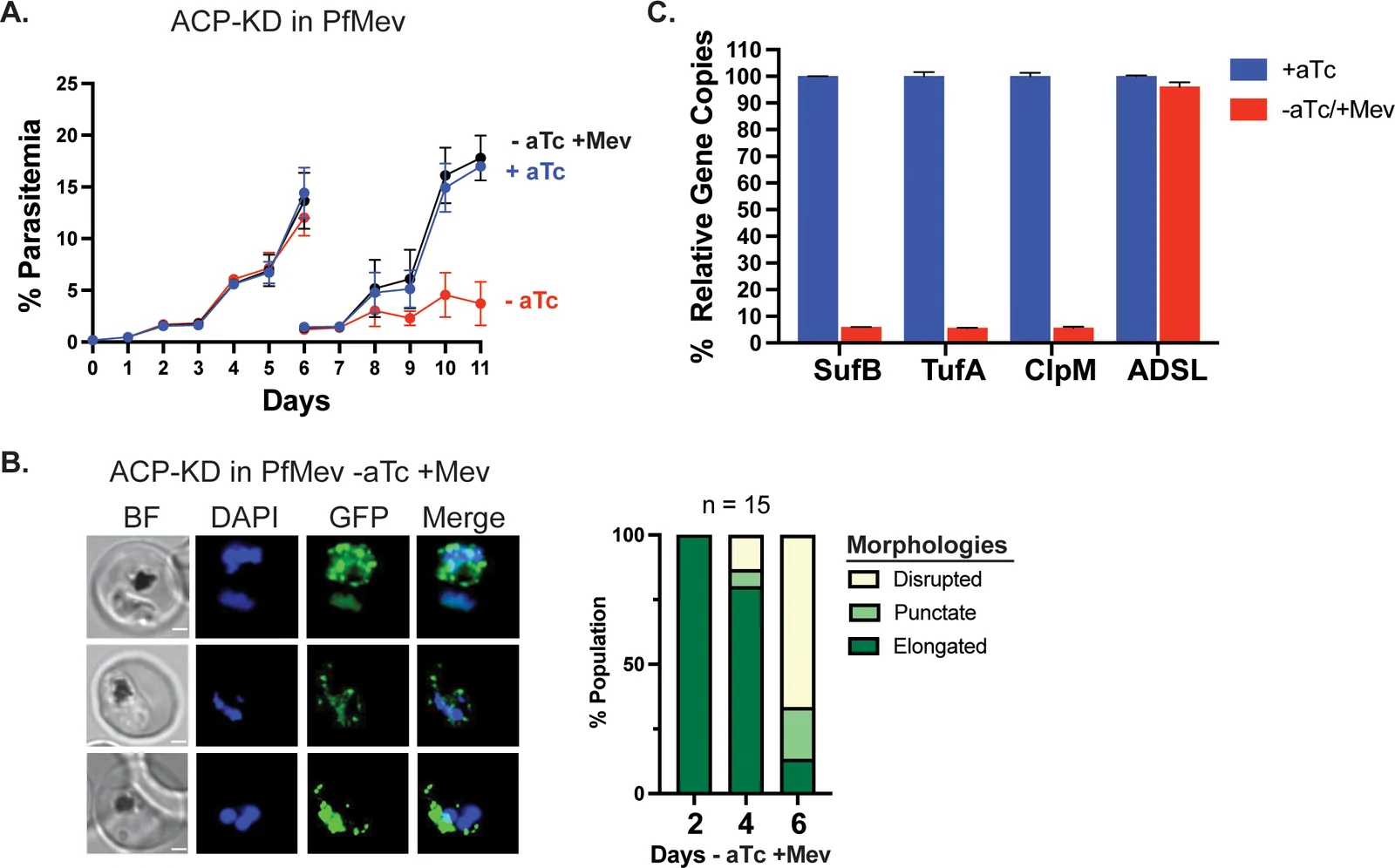
Synchronous growth assays (**A**), fluorescence microscopy (**B**), and genomic quantitative PCR (qPCR) (**C**) of acyl carrier protein (ACP) knockdown in PfMev NF54 parasites. For the growth assay in panel A, cultures were split on day 6. Samples for genomic qPCR were cultured as indicated for ≥8 days. Gene copy numbers were based on amplification of apicoplast (SufB: Pf3D7_API04700, ClpM: Pf3D7_API03600, TufA: Pf3D7_API02900) or nuclear (ADSL: Pf3D7_0206700) relative to nuclear (STL: Pf3D7_0717700, I5P: Pf3D7_0802500) genes. Indicated qPCR ratios were normalized to +aTc and are the average ± SD of biological triplicates. Scale bars ~1 µm.

**Figure 2—figure supplement 4.**
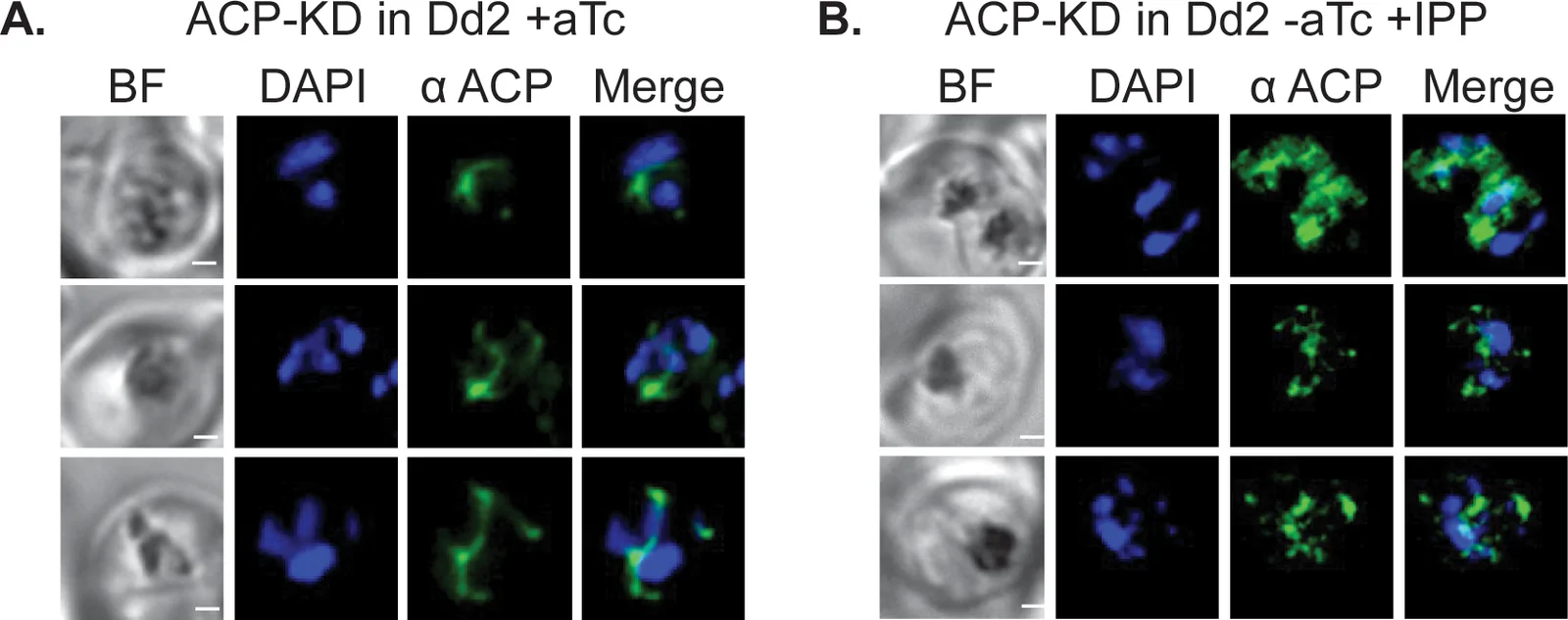
Additional immunofluorescence assay (IFA) images for apicoplast morphology upon acyl carrier protein (ACP) knockdown in Dd2 parasites in +aTc (**A**) or –aTc/+isopentenyl pyrophosphate (IPP) (**B**) conditions. BF = bright field. Scale bars ~1 µm.

**Figure 2—figure supplement 5.**
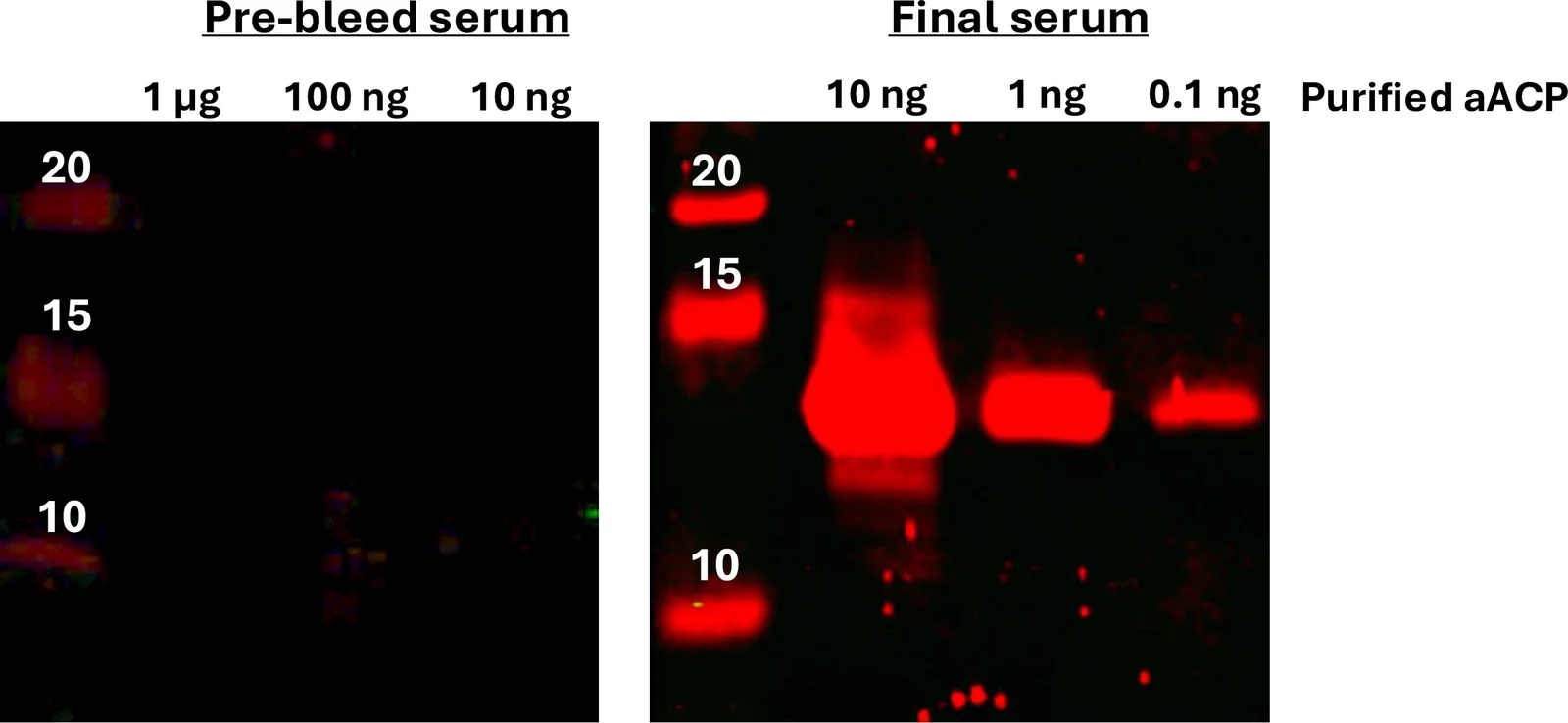
Validation of custom anti-apicoplast acyl carrier protein (aACP) antibody. Indicated amount of purified aACP antigen was loaded and fractionated on SDS-PAGE gel, transferred to membrane, blocked, and probed with 1:1000 dilution of rabbit pre-bleed serum or final serum, washed, and probed with donkey anti-rabbit IRDye680 secondary antibody. Figure 2—figure supplement 5—source data 1.PDF containing the uncropped gel image and labels for Figure 2—figure supplement 5. Figure 2—figure supplement 5—source data 2.Original file for western blot analysis in Figure 2—figure supplement 5.

To further test this conclusion and to dissect ACP function in the apicoplast, we used CRISPR/Cas9 to tag the aACP gene in both Dd2 and PfMev NF54 parasites to encode a C-terminal HA-FLAG epitope tag fusion and the aptamer-TetR/DOZI system for conditional knockdown (Ganesan et al., 2016). Successful integration in clonal parasites for both lines was confirmed by genomic PCR (Figure 2—figure supplement 1). Parasites grew normally in the presence of exogenous anhydrotetracycline (aTc), which enables normal aACP translation and detection of HA-FLAG-tagged ACP at the expected size by western blot analysis (Figure 2C). However, parasite growth was strongly suppressed after 2–3 cycles following aTc washout that resulted in loss of detectable aACP expression (Figure 2C, Figure 2—figure supplement 3). In both Dd2 and PfMev parasites, culture supplementation with exogenous IPP or mevalonate, respectively, fully restored normal parasite growth in the absence of aTc, confirming an apicoplast-specific defect upon ACP knockdown. Fluorescence microscopy and genomic quantitative PCR (qPCR) analysis in IPP-rescued parasites confirmed disruption of the apicoplast and selective loss of the apicoplast genome upon sustained culture without aTc (Figure 2D and E, Figure 2—figure supplement 2, and Figure 2—figure supplement 3). These results strongly support the conclusion that aACP is essential for parasite viability and organelle biogenesis.

### Blood-stage parasites require ACP modification by holo-ACP synthase but not FabD

Canonical ACP function requires modification by holo-ACP synthase (ACPS), which uses coenzyme A to transfer a 4-PP group to a highly conserved Ser residue on ACP (Figure 1). aACP retains this conserved Ser (Figure 1), and parasites express an apicoplast-targeted ACPS enzyme (Pf3D7_0420200) (Ralph et al., 2004; Cory et al., 2017), as expected for 4-PP attachment to ACP to support essential FASII activity in mosquito- and liver-stage parasites (van Schaijk et al., 2014; Yu et al., 2008). Because FASII activity is dispensable in blood-stage parasites, the modification state of aACP and role of ACPS in this stage have been uncertain (Gallagher and Prigge, 2010).

**Figure 3.**
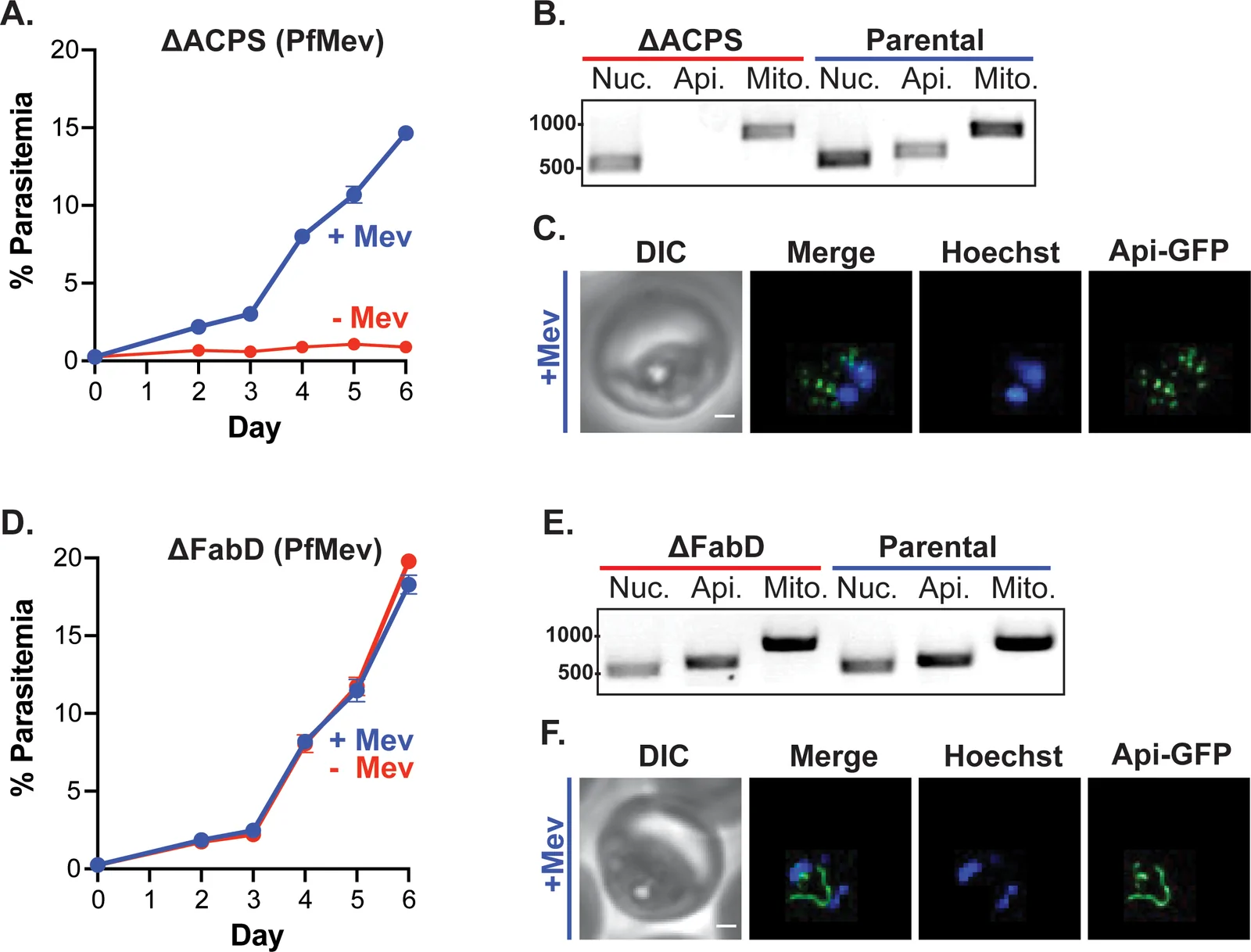
Holo-acyl carrier protein (ACP) synthase (ACPS) but not FabD is essential for blood-stage parasites and apicoplast biogenesis. (**A**) Synchronous growth assay of ∆ACPS PfMev parasites cultured ±50 µM mevalonate. (**B**) Genomic PCR analysis and (**C**) live parasite imaging show selective loss of the apicoplast genome and disrupted apicoplast morphology based on fluorescence of the ACP_L_-GFP marker protein in PfMev parasites. (**D**) Synchronous growth assay of ∆FabD PfMev parasites cultured ±50 µM mevalonate. (**E**) Genomic PCR analysis and (**F**) live parasite imaging indicate retention of the apicoplast genome and normal apicoplast morphology based on ACP_L_-GFP fluorescence in ∆FabD parasites. Parasitemia values are the average ± SD from biological triplicates. Nuc.=nuclear gene (LDH), Api.=apicoplast gene (SufB), Mito.=mitochondrial gene (CoxI), DIC = differential interference contrast. Scale bars ~1 µm. Figure 3—source data 1.PDF containing uncropped gel images for apicoplast status PCRs for Figure 3B and E. Figure 3—source data 2.Original files for PCR gel analyses in Figure 3B and E.

To directly test if ACPS function is essential for blood-stage parasites, we targeted the ACPS gene for deletion in PfMev NF54 parasites. Like aACP, parasites only returned from transfection in the presence of exogenous mevalonate, and genomic PCR analysis confirmed disruption of the ACPS gene (Figure 3—figure supplement 1). Growth of ∆ACPS parasites was strictly dependent on mevalonate (Figure 3A), and analysis by genomic PCR (Figure 3B) fluorescence microscopy (Figure 3C and Figure 3—figure supplement 2) revealed disruption of apicoplast morphology and selective loss of the apicoplast genome. These results indicate that ACPS function is essential for parasite viability and apicoplast biogenesis, as expected if 4-PP attachment is required for essential aACP function in blood-stage parasites. Additional biochemical results described below also support the conclusion that essential aACP function requires 4-PP attachment.

**Figure 3—figure supplement 1.**
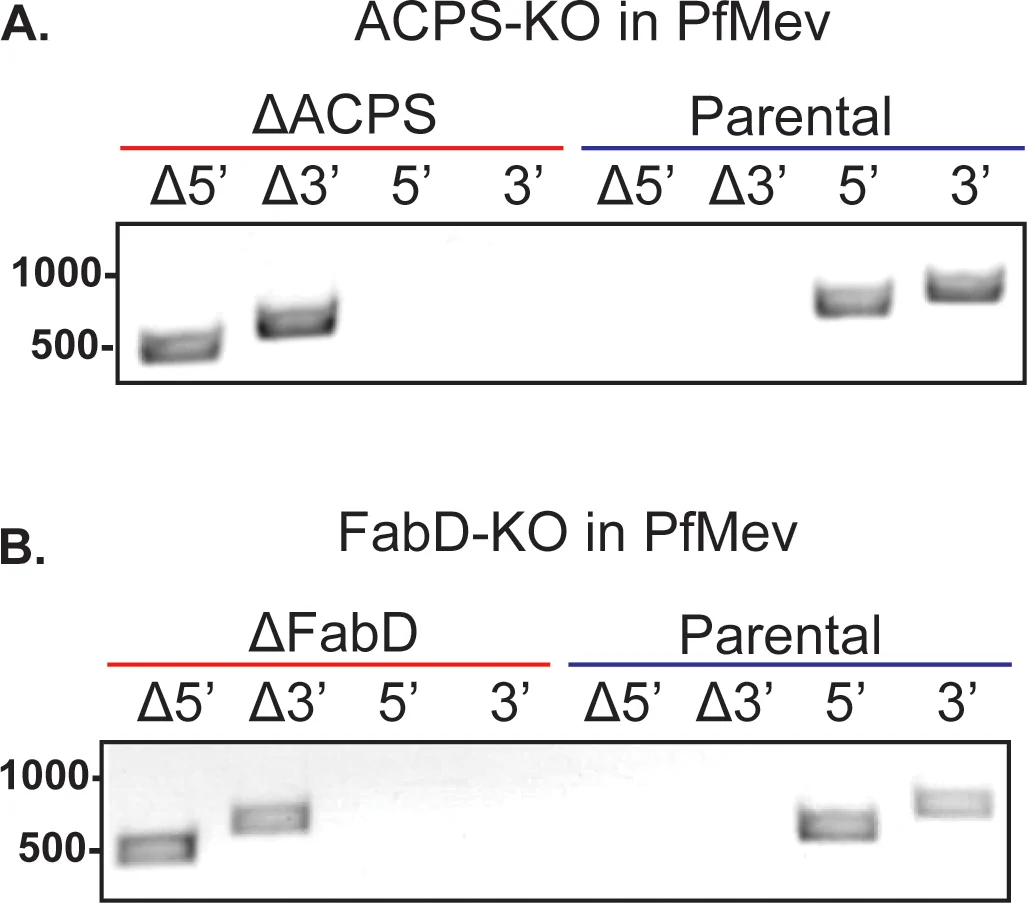
Integration PCRs for deletion of acyl carrier protein synthase (ACPS) (**A**) or FabD (**B**) in PfMev parasites. Figure 3—figure supplement 1—source data 1.PDF containing uncropped gel images for integration PCRs for Figure 3—figure supplement 1. Figure 3—figure supplement 1—source data 2.Original files for integration PCR gel analyses in Figure 3—figure supplement 1.

**Figure 3—figure supplement 2.**
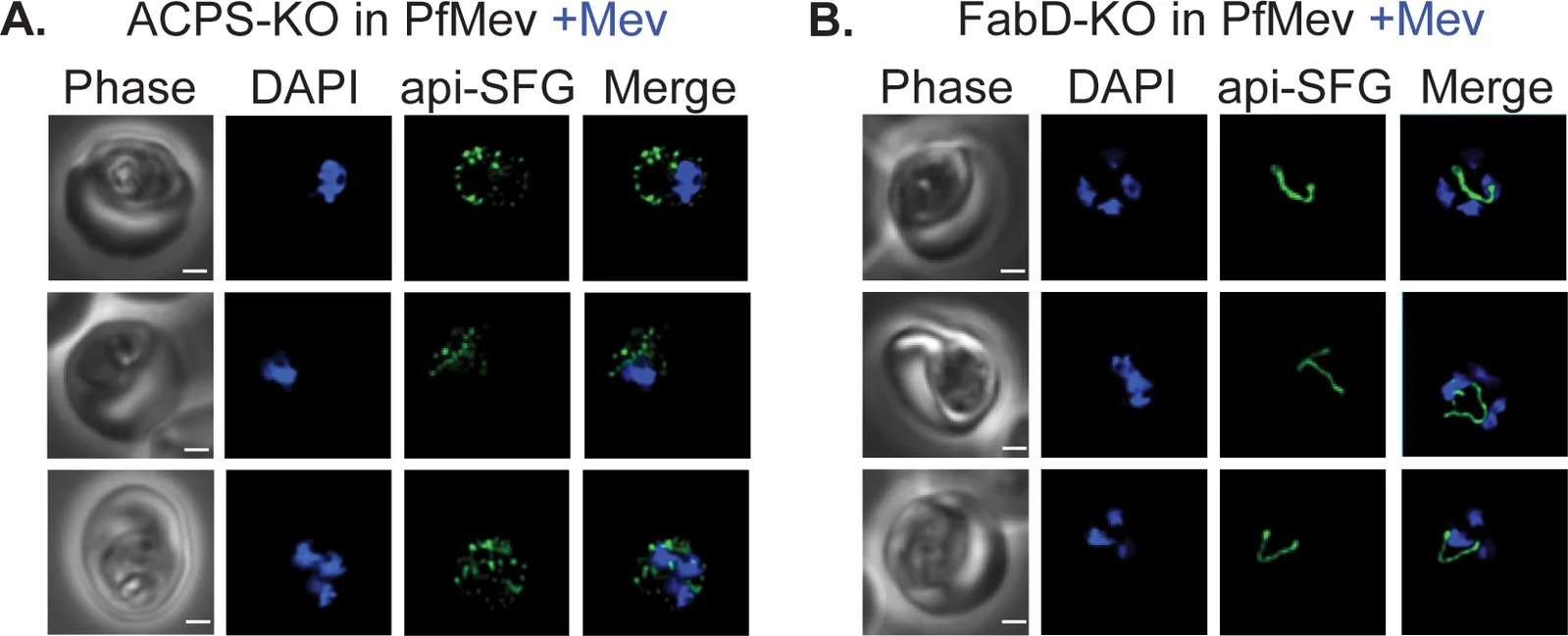
Additional microscopy images of apicoplast morphology in ∆ACPS (**A**) and ∆FabD (**B**) PfMev parasites. ACPS = acyl carrier protein synthase, Phase = phase contrast, api-SFG = apicoplast-targeted superfolder GFP. Scale bars ~1 µm.

**Figure 3—figure supplement 3.**
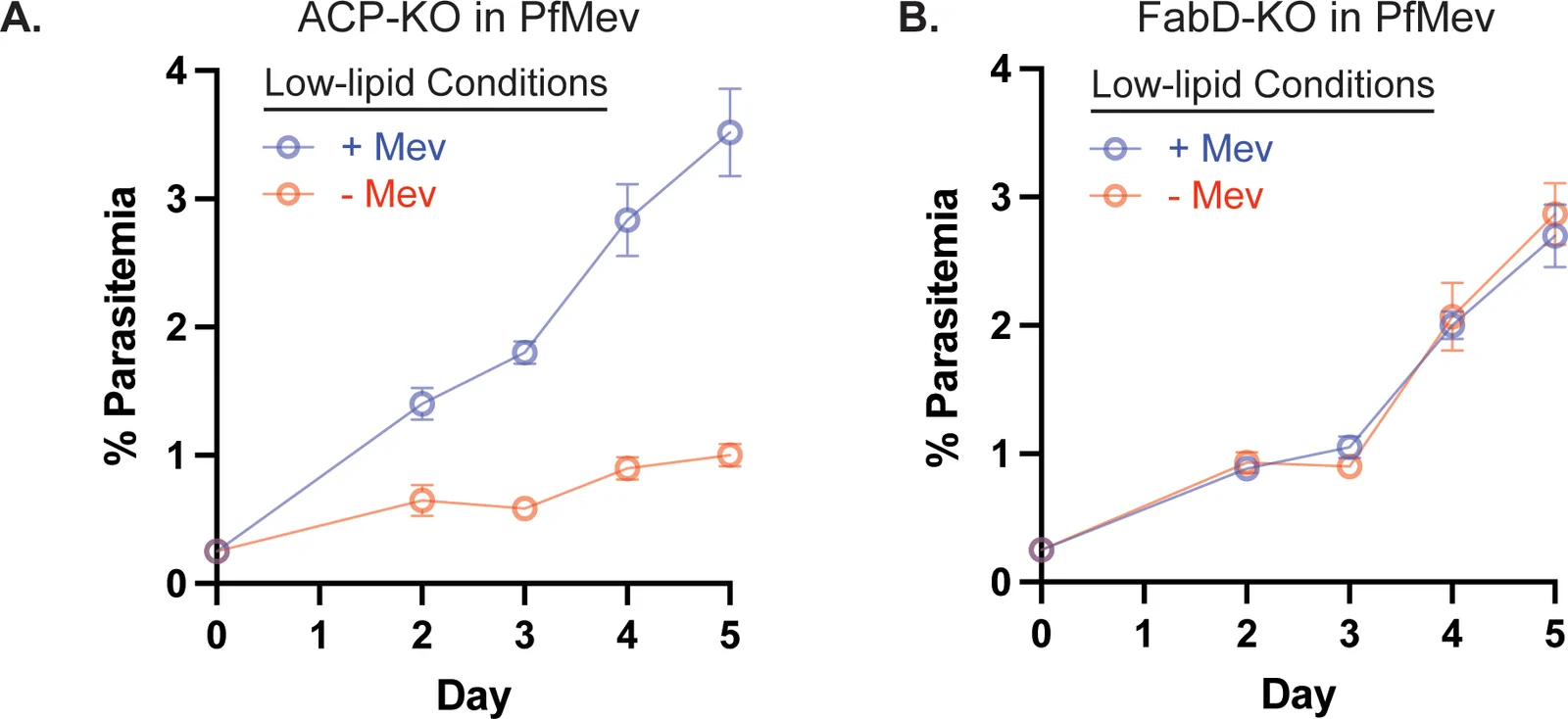
Growth of ∆ACP (**A**) and ∆FabD (**B**) PfMev parasites in low-lipid conditions with daily media changes. ACP = acyl carrier protein.

The initial step in FASII activity involves FabD-catalyzed transfer of a malonyl group from malonyl-CoA to holo-ACP to form malonyl-ACP (Figure 1). Although downstream FASII enzymes that catalyze successive modification and elongation of malonyl-ACP into acyl chains of variable length have been successfully deleted in blood-stage *P. falciparum* (Shears et al., 2015), there are no reports of targeted disruption of FabD (Pf3D7_1312000). Prior genome-wide studies in blood-stage parasites have reported conflicting results, with FabD listed as essential in *P. falciparum* (Zhang et al., 2018) but dispensable in *P. berghei* (Bushell et al., 2017). To test if aACP modification by FabD is required for essential aACP function, we targeted FabD for deletion in PfMev NF54 parasites. Parasites returned from transfection without exogenous mevalonate, and genomic PCR analysis confirmed successful deletion of the FabD gene (Figure 3—figure supplement 1). Growth of ∆FabD parasites was identical in ±mevalonate conditions (Figure 3D), and analysis by genomic PCR (Figure 3E) and fluorescence microscopy (Figure 3F, Figure 3—figure supplement 2) indicated normal apicoplast morphology and genome status. We conclude that FabD, like other FASII enzymes, is dispensable for blood-stage *P. falciparum* and not required for essential aACP function.

Prior isotope-tracing studies suggested that apicoplast FASII is largely inactive in blood-stage parasites growing in standard media but may be activated in low-lipid conditions (Botté et al., 2013; Ouji et al., 2024). A subsequent study thus suggested that the apicoplast FASII pathway might be essential for blood-stage *P. falciparum* growth in low-lipid conditions (Amiar et al., 2020). We tested this suggestion by growing ∆ACP and ∆FabD PfMev parasites in low-lipid conditions, in which Albumax was replaced in the culture medium with lipid-free bovine serum albumin supplemented with oleic and palmitic acids with daily media changes (Botté et al., 2013; Dellibovi-Ragheb et al., 2018). Both knockout lines grew steadily in low-lipid conditions, and ∆ACP parasites with disrupted apicoplast retained exclusive dependence on exogenous mevalonate for viability (Figure 3—figure supplement 3). These results contrast with the prior study (Amiar et al., 2020) of ∆FabI parasites and the proposed model that blood-stage *P. falciparum* requires FASII activity for growth in low-lipid conditions and suggest that parasites can rely on scavenging host-derived fatty acids over a wide range of lipid conditions. Future studies involving tandem growth and isotope-labeling experiments of WT and ∆FASII parasites will be required to fully test and understand FASII function and the dependence of *P. falciparum* growth on this pathway in low-lipid conditions.

### ACP associates with apicoplast PKII using its 4-PP group

Beyond its role in FASII, mitochondrial ACP (mACP) in other eukaryotes binds to LYR motif adapter proteins that interact with diverse protein partners involved in Fe-S cluster biogenesis and assembly of mitochondrial respiratory complexes and (in humans) mitoribosomes (Masud et al., 2019; Angerer, 2015). Expanded roles for ACP beyond FASII in chloroplasts and other plastids are sparsely defined, and there is no evidence for LYR motif proteins in plastids, including the *P. falciparum* apicoplast (Falekun et al., 2021; Dohnálek and Doležal, 2024). Nevertheless, based on strong biochemical precedent that ACP in mitochondria and bacteria engage in a variety of critical protein-protein interactions, we hypothesized that essential ACP function in the parasite apicoplast might involve an essential interaction with one or more key proteins required for organelle function and biogenesis (Gully et al., 2003; Cronan, 2023).

**Figure 4.**
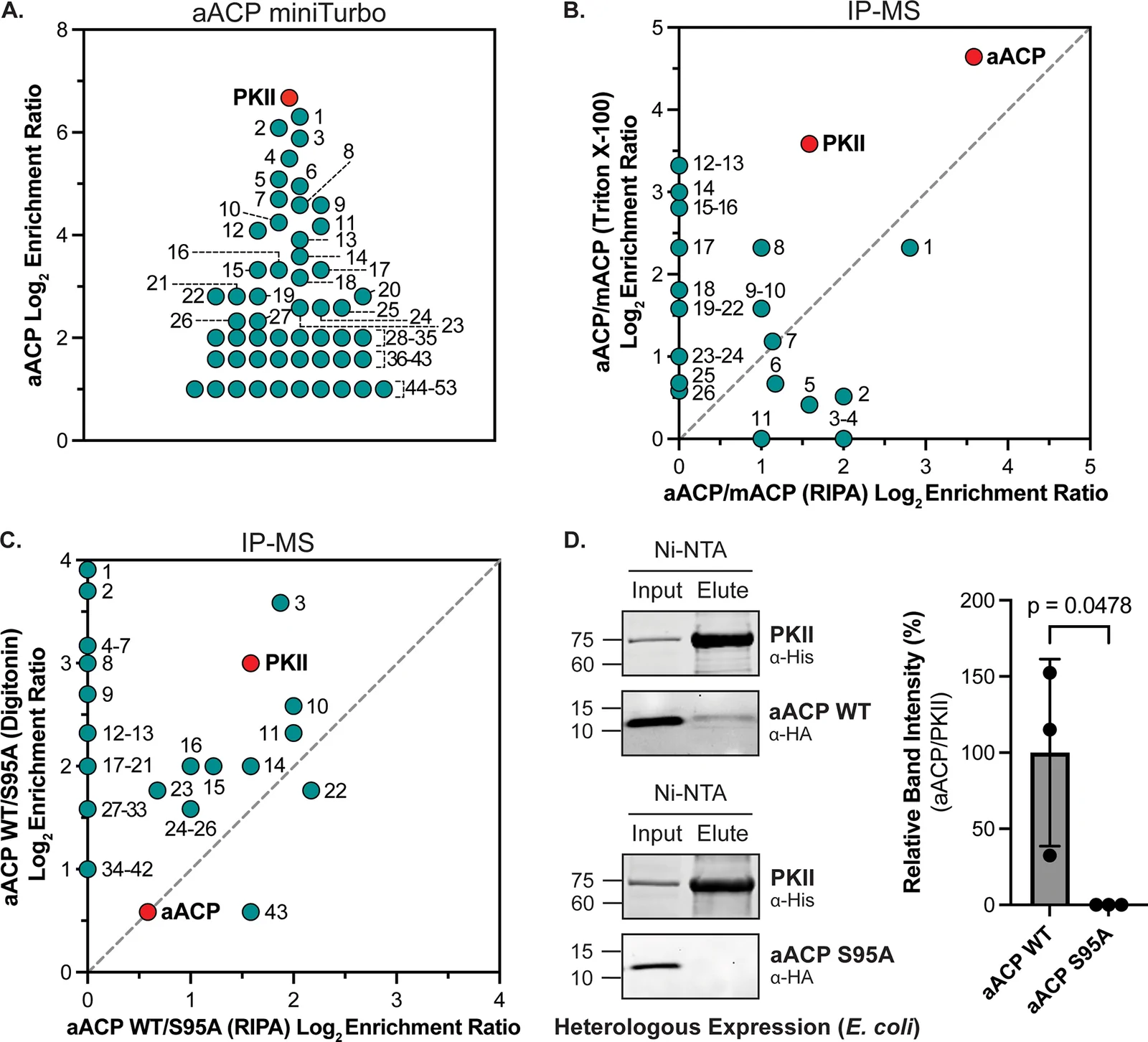
Apicoplast acyl carrier protein (ACP) associates with apicoplast pyruvate kinase II. (**A**) Proximity biotinylation study of Dd2 parasites episomally expressing apicoplast ACP tagged with C-miniTurbo, showing log_2_ enrichment ratio of known apicoplast-targeted proteins compared to parental Dd2 parasites, based on spectral intensity of proteins detected by tandem mass spectrometry. (**B**) Immunoprecipitation-mass spectrometry (IP-MS) study of apicoplast ACP interacting proteins, based on anti-HA-tag IP of apicoplast versus mitochondrial ACP (mACP)-HA_2_ in Dd2 parasites lysed in Triton X-100 or RIPA buffer. Axes display the log_2_ enrichment ratio for detection of each protein in IP samples of aACP versus mACP. Dashed diagonal line has a slope of one. (**C**) IP-MS analysis based on anti-HA-tag IP of lysates from Dd2 parasites episomally expressing wild-type (WT) or the S95A mutant of apicoplast ACP-HA_2_ and lysed in digitonin or RIPA buffer. Axes display the log_2_ enrichment ratio for detection of each protein in IP samples of WT versus S95A ACP. Dashed diagonal line has a slope of one. The identity of numbered proteins in panels A–C is shown in Supplementary file 1. A list of all proteins detected by mass spectrometry is shown in Figure 4—source data 3. (**D**) Affinity pull-down and western blot analysis of lysates from *E. coli* bacteria heterologously expressing parasite His_6_-tagged PKII or apicoplast ACP-HA_2_ (WT or S95A mutant). PKII was affinity isolated from bacterial lysates using nickel-nitrilotriacetic acid (Ni-NTA) resin, eluted with free imidazole, and analyzed by western blot for co-purification with aACP. Membranes were probed with anti-His_6_ and anti-HA-tag antibodies. The signal intensity for WT or S95A ACP relative to PKII in pull-down samples was quantified by densitometry in biological triplicate samples and plotted as the average ± SD, with significance analyzed by Student’s t-test to determine the indicated p-value. Figure 4—source data 1.PDF containing uncropped gels for western blot analyses in Figure 4D. Figure 4—source data 2.Original files for western blot gel images in Figure 4D. Figure 4—source data 3.Excel file of all proteins detected in proximity biotinylation and immunoprecipitation studies of apicoplast acyl carrier protein (ACP).

To identify proximal interacting proteins of the aACP, we first took a proximity biotinylation approach by episomally expressing C-terminal fusions of aACP with miniTurbo or BioID2 in blood-stage Dd2 *P. falciparum* (Branon et al., 2018; Kim et al., 2016). Biotinylation of proteins that spanned a range of molecular masses was exclusively detected in transgenic but not parental Dd2 parasites (Figure 4—figure supplement 1). Labeled proteins were identified by tandem mass spectrometry after pull-down with streptavidin resin, stringent washes, and tryptic digest. Pull-downs of parental Dd2 parasites served as negative control. Over 50 apicoplast-targeted proteins were detected as specifically enriched in parasites expressing ACP-miniTurbo, with apicoplast PKII (Pf3D7_1037100) showing the strongest enrichment (Figure 4A and Supplementary file 1 – supplementary table 1). Comparatively fewer proteins were identified from ACP-BioID2-expressing parasites, but PKII was one of two apicoplast-targeted proteins detected (Figure 4—figure supplement 2). We thus considered the possibility that aACP had a functionally relevant interaction with PKII.

**Figure 4—figure supplement 1.**
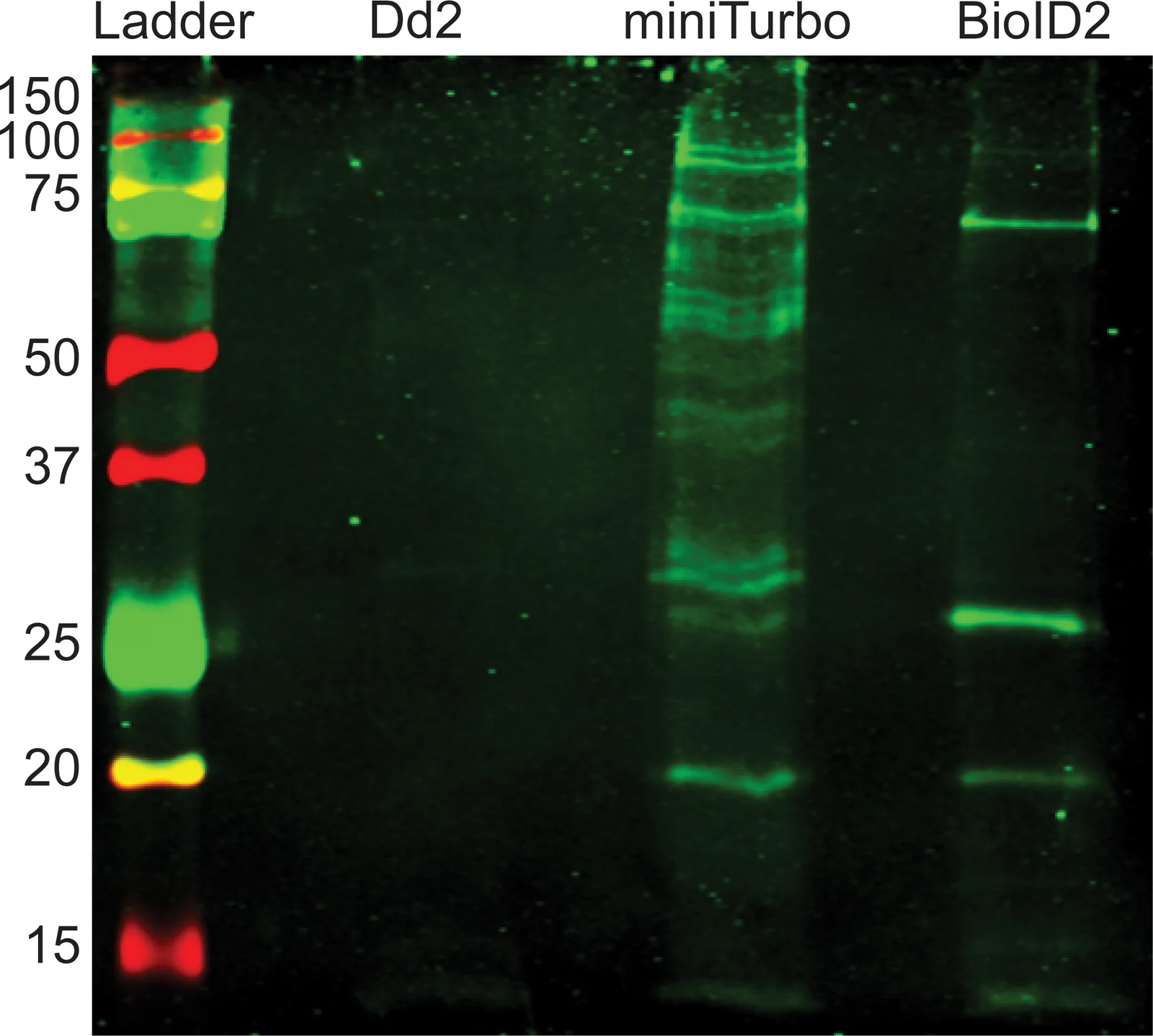
Western blot detection of biotinylated proteins in lysates from parental, apicoplast acyl carrier protein (aACP)-miniTurbo, or aACP-BioID2 Dd2 parasites. Figure 4—figure supplement 1—source data 1.PDF containing uncropped gel image for Figure 4—figure supplement 1. Figure 4—figure supplement 1—source data 2.Original files for western blot gel analysis in Figure 4—figure supplement 1.

**Figure 4—figure supplement 2.**
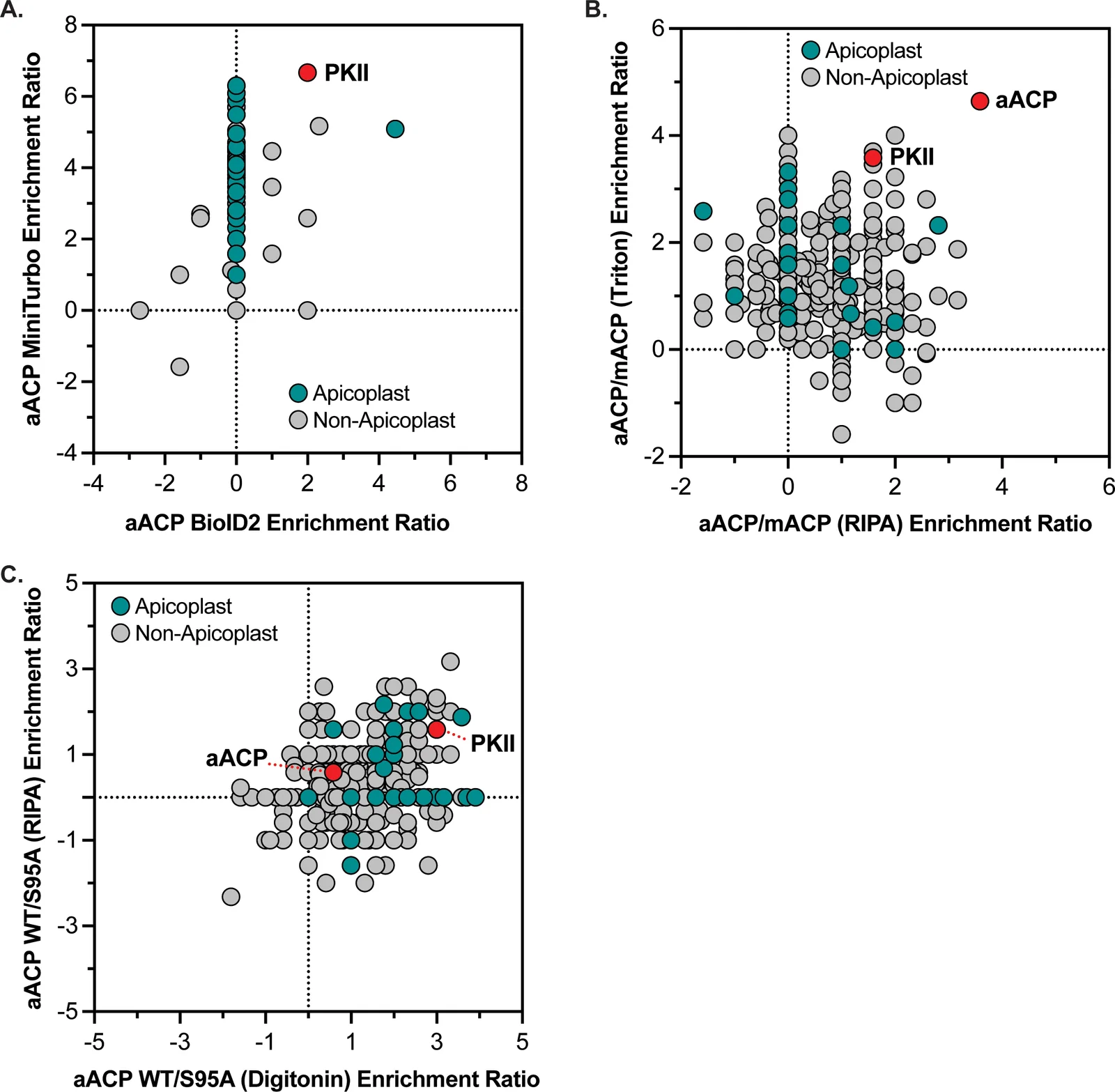
Enriched protein interactors of apicoplast acyl carrier protein (ACP) detected by proximity biotinylation or immunoprecipitation (IP). (**A**) Apicoplast ACP interactors identified by miniTurbo versus BioID2. (**B**) Interacting proteins of ACP enriched in IP samples of apicoplast compared to mitochondrial ACP in Triton X-100 or RIPA buffer. (**C**) Interacting proteins of ACP enriched in IP samples of wild-type (WT) compared to S95A ACP in RIPA or digitonin buffer. All axes display the log_2_ enrichment ratio.

**Figure 4—figure supplement 3.**
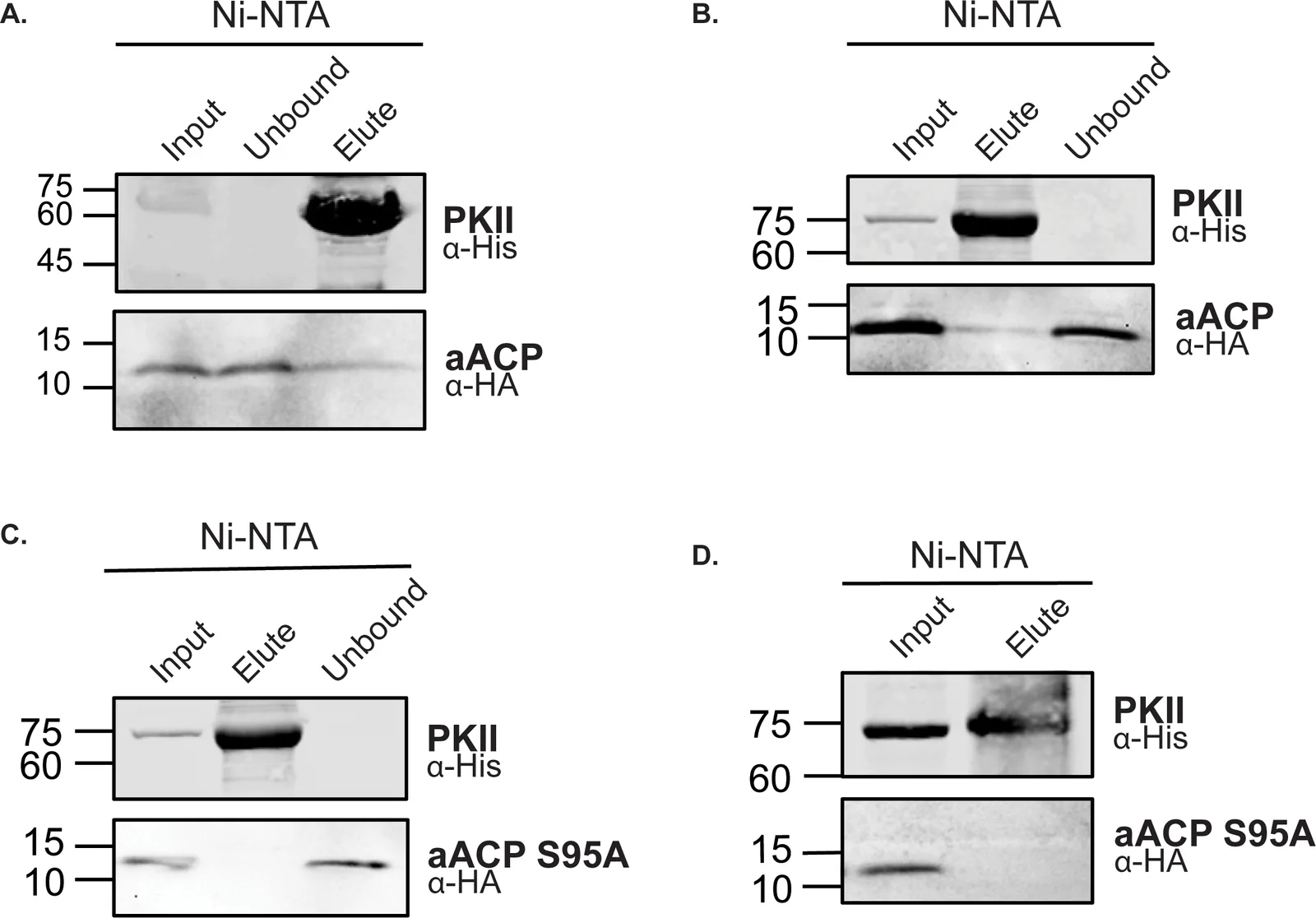
Replicate western blot images for Ni-NTA pull-down of pyruvate kinase II (PKII) association with wild-type (WT) (**A, B**) or Ser95Ala (**C, D**) acyl carrier protein (ACP) in *E. coli*. Figure 4—figure supplement 3—source data 1.PDF containing uncropped gel image for Figure 4—figure supplement 3. Figure 4—figure supplement 3—source data 2.Original files for western blot gel analysis in Figure 4—figure supplement 3.

Proximity biotinylation does not require stable interaction between bait and labeled proteins. To identify apicoplast proteins that stably associate with aACP, we performed immunoprecipitation/tandem mass spectrometry (IP/MS) studies of Dd2 parasites episomally expressing ACP with a C-terminal HA_2_ tag. We lysed parasites in either RIPA or 1% Triton X-100 buffer, with parasites expressing mACP-HA_2_ (Pf3D7_1208300) serving as negative control (Falekun et al., 2021). Over 20 apicoplast-targeted proteins were identified in both lysis conditions, with PKII identified as the most or nearly most-enriched protein interactor of aACP in both samples (Figure 4B, Figure 4—figure supplement 2, and Supplementary file 1 – supplementary table 2). Apicoplast PKII is an essential enzyme whose production of pyruvate and a range of NTPs and dNTPs is critical to support apicoplast biogenesis and nearly all apicoplast metabolic pathways, including IPP synthesis by the non-mevalonate pathway, Fe-S cluster biogenesis by the Suf pathway, DNA replication, RNA transcription, and protein translation (Swift et al., 2020a). On the basis of this essentiality and consistent detection of PKII as the most or nearly most-enriched interactor of aACP across four proximity biotinylation and IP/MS experiments, we focused our attention on the association of aACP and PKII and the physiological consequence of this interaction.

Our observation that loss of ACPS impairs apicoplast biogenesis (Figure 3) suggested most simply that essential function of aACP requires post-translational attachment of 4-PP. To test a role for 4-PP attachment to aACP in mediating association with PKII, we performed IP/MS studies of parasites episomally expressing wild-type (WT) or the Ser95Ala mutant of ACP-HA_2_. Ser95 is the strictly conserved ACP residue required for 4-PP attachment by ACPS (Figure 1; Gallagher and Prigge, 2010), and its mutation thus ablates the ability of ACP to covalently bind 4-PP (Falekun et al., 2021). Parasites were lysed in either RIPA or digitonin buffer prior to identification of associated proteins by anti-HA-tag IP/MS. Over 40 apicoplast-targeted proteins were identified across both lysis conditions as enriched in samples of WT over Ser95Ala ACP (Figure 4C, Figure 4—figure supplement 2). PKII was strongly detected as interacting with WT ACP but was not detected in IP/MS samples of Ser95Ala ACP (Supplementary file 1 – supplementary table 3), strongly suggesting that the 4-PP group on ACP contributes critically to its association with PKII.

Our IP experiments in parasites cannot distinguish if association between aACP and PKII is direct or indirect or requires other apicoplast proteins. To test if aACP can bind to PKII without other *Plasmodium* proteins, we heterologously expressed ACP-HA_2_ and His_6_-PKII in *E. coli* bacteria and performed affinity pull-downs of PKII using Ni-NTA resin to bind the His_6_ tag. The expressed proteins corresponded to the mature sequences expected upon apicoplast import and N-terminal processing (Swift et al., 2020a; van Dooren et al., 2002; Falekun et al., 2021). Recombinant PKII isolated in this fashion co-purified with WT but not Ser95Ala ACP (Figure 4D, Figure 4—figure supplement 3). These observations support the conclusion that aACP can bind to PKII without other parasite proteins, as expected for a direct interaction, and that the 4-PP group on aACP contributes critically to this association.

### Loss of ACP destabilizes PKII and impairs apicoplast DNA and RNA levels

**Figure 5.**
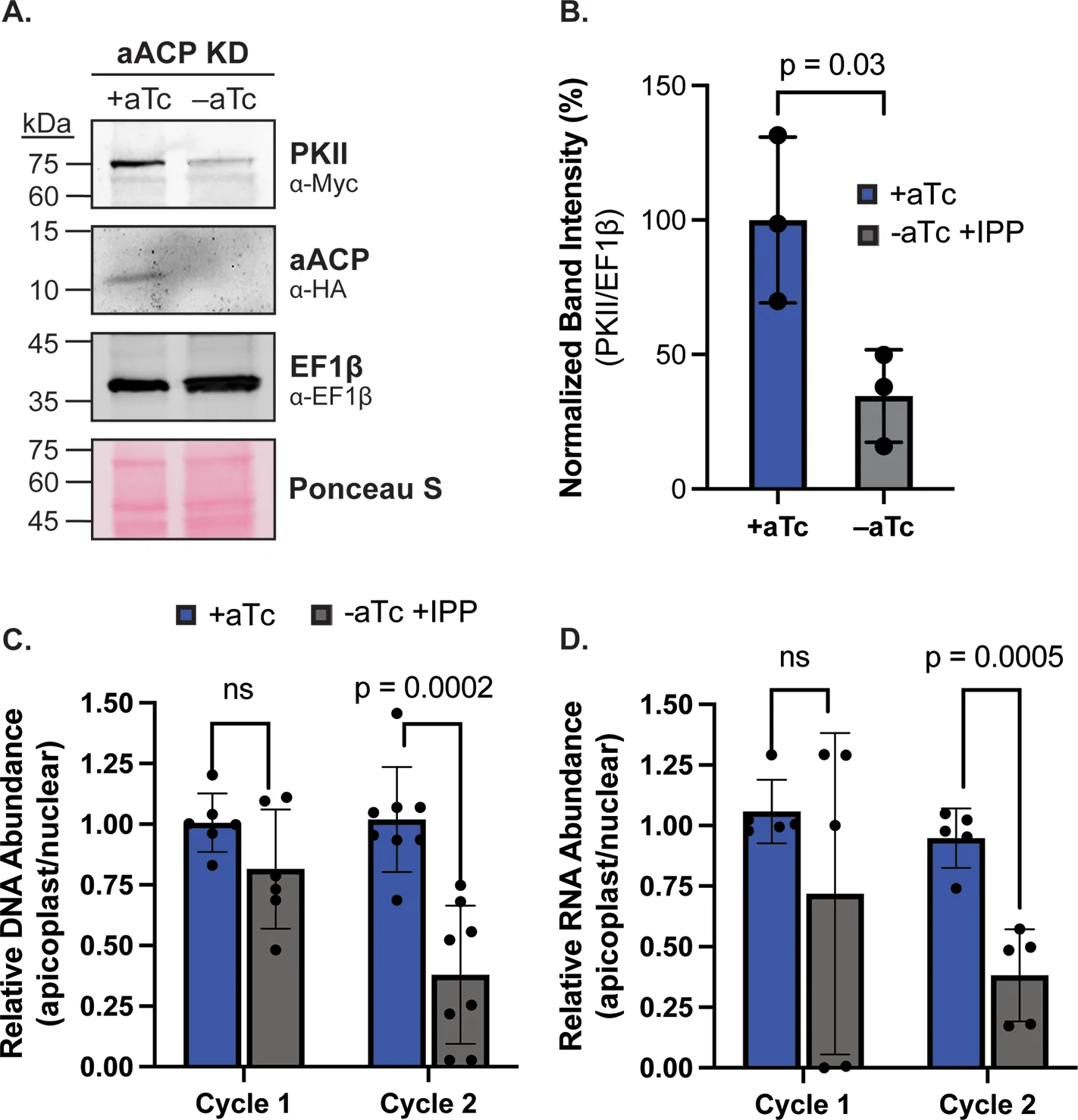
Acyl carrier protein (ACP) knockdown destabilizes pyruvate kinase II (PKII) and impairs apicoplast DNA and RNA levels. (**A**) Western blot analysis of apicoplast ACP-aptamer/TetR-DOZI Dd2 parasites episomally expressing PKII C-Myc_3_ and cultured for 3 days±1 µM aTc with 200 µM isopentenyl pyrophosphate (IPP). The blot was probed with α-Myc (PKII), α-HA (ACP), and α-ΕF1β (cytosolic loading control) antibodies and stained with Ponceau S for total protein. (**B**) Densitometry quantification of PKII and aACP protein levels normalized to ΕF1β based on western blot analysis of three biological replicates. Graph depicts the average ± SD, normalized to +aTc conditions. (**C**) Quantitative PCR analysis of DNA isolated from biological triplicate samples of apicoplast ACP-aptamer/TetR-DOZI parasites cultured for 36 or 84 hr±1 µM aTc with 200 µM IPP, with normalization of Ct values averaged from three apicoplast genes (SufB, TufA, ClpM) to Ct values averaged from three nuclear genes (STL, I5P, ADSL). Graphs depict the average ± SD of each timepoint, normalized to +aTc conditions. (**D**) Quantitative RT-PCR analysis of RNA isolated from biological triplicate samples obtained from parasites cultured under identical conditions as for panel C to determine normalized levels of apicoplast (SufB, TufA, ClpM) relative to nuclear (STL, I5P, ADSL) transcripts. Indicated p-values were determined by Student’s t-test analysis. Figure 5—source data 1.PDF containing the uncropped western blot images for Figure 5A. Figure 5—source data 2.Original image files for western blot analysis in Figure 5A.

In eukaryotes, mACP interaction with key protein partners contributes to their stability and function, such that loss of ACP destabilizes associating proteins and can lead to their degradation (Masud et al., 2019; Van Vranken et al., 2016). Based on this biochemical precedent, we posited that apicoplast association might enhance the stability of PKII, such that loss of aACP would destabilize and impair PKII levels and function and thus explain the lethal defect in apicoplast biogenesis observed upon ACP knockdown in *P. falciparum*. To test this model, we transfected Dd2 aACP knockdown parasites with a plasmid encoding expression of PKII with C-terminal Myc_3_ tag. We observed that aACP knockdown after 3 days of growth in –aTc/+IPP conditions resulted in selective ~75% reduction in detectable PKII levels by western blot analysis (Figure 5A and B, Figure 5—figure supplement 1). This observation directly supports our model that aACP association enhances the stability and levels of PKII in the parasite apicoplast.

**Figure 5—figure supplement 1.**
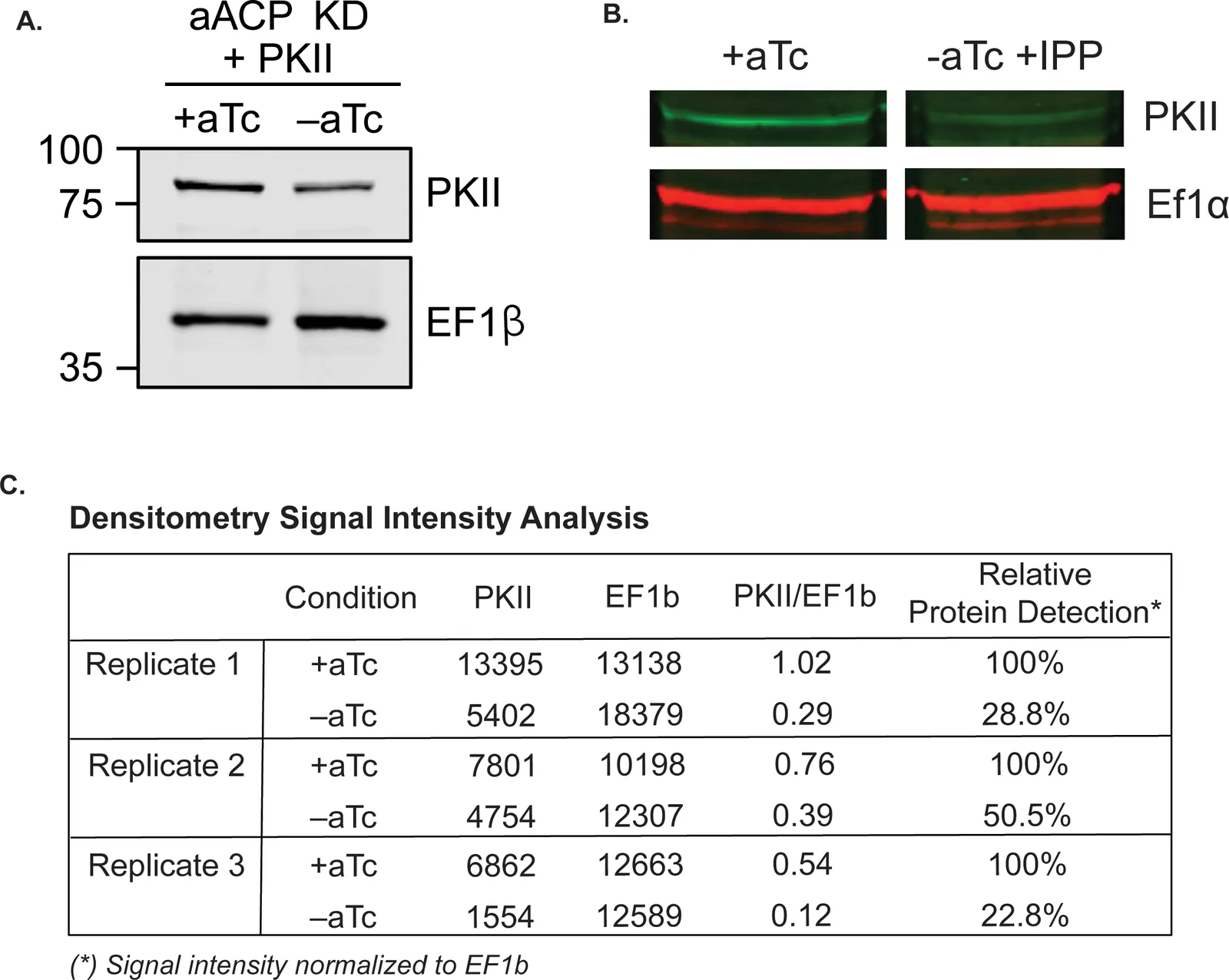
Replicate western blot images for acyl carrier protein (ACP) knockdown and pyruvate kinase II (PKII) levels. Indicated masses in panels A and B are kDa. Figure 5—figure supplement 1—source data 1.PDF containing uncropped gel images for Figure 5—figure supplement 1. Figure 5—figure supplement 1—source data 2.Original files for western blot gel analysis in Figure 5—figure supplement 1.

Current evidence suggests that PKII is the dominant or exclusive source of NTPs and dNTPs in the apicoplast, with PKII knockdown causing defects in apicoplast genome transcription and ATP levels (Swift et al., 2020a). To test if aACP knockdown and attendant decreases in PKII levels substantially reduced apicoplast-encoded DNA and RNA levels, we used qPCR and RT-qPCR to quantify apicoplast- versus nuclear-encoded gene and transcript levels in +aTc or –aTc/+IPP conditions in the first and second RBC growth cycles prior to apicoplast disruption. No significant differences in normalized DNA or RNA levels were observed in the first growth cycle ±aTc. However, aACP knockdown in –aTc conditions resulted in a nearly 70% reduction in apicoplast DNA and RNA levels in the second cycle, as expected for loss of PKII. This critical role for aACP in stabilizing PKII is sufficient to explain FASII-independent function and essentiality of aACP.

## Discussion

Organelles are central hubs of cellular metabolism and key drivers of eukaryotic evolution, including biochemical adaptations that specialize intracellular parasites to survive and grow within host cells. The apicoplast FASII pathway in *P. falciparum* has generated substantial interest and controversy as a possible antimalarial drug target (Shears et al., 2015). This pathway was initially thought to be essential for blood-stage parasites (Surolia and Surolia, 2001), but genetic knockouts of most FASII enzymes and isotope-tracing studies indicated that it was dispensable and largely inactive for parasites during RBC infection (Shears et al., 2015; van Schaijk et al., 2014; Botté et al., 2013). In contrast to FASII, we have uncovered an essential blood-stage function for the aACP and its 4-PP prosthetic group in binding and stabilizing PKII, which supports nearly all aspects of apicoplast function and biogenesis. This essential interaction, which depends on the ACPS enzyme for 4-PP attachment to aACP, is independent of canonical ACP function as a soluble scaffold for short-chain fatty acid synthesis and elongation during FASII. This discovery critically expands understanding of ACP function in the *Plasmodium* apicoplast and suggests that aACP has evolved an adaptive role at a key molecular nexus controlling broad organelle metabolism.

### Molecular association of ACP and PKII

Our results reveal that PKII protein levels depend critically on aACP expression, with stringent aACP knockdown resulting in a 75% decrease in PKII abundance (Figure 5B). We detected aACP interaction with PKII in parasites by both proximity biotinylation and IP followed by mass spectrometry (Figure 4). This interaction persisted during heterologous expression in *E. coli* and in the absence of other parasite-specific proteins, suggesting a direct binding interaction between PKII and aACP.

We noted that co-purification of aACP with PKII appeared to be sub-stoichiometric in pull-down experiments from bacteria, which may reflect a modest-affinity interaction that partially dissociates under non-equilibrium pull-down and wash conditions. *P. falciparum* aACP is known to be modified by a 4-PP group in *E. coli* and to functionally replace bacterial ACP in FASII (Gallagher and Prigge, 2010; De Lay and Cronan, 2007; Waters et al., 2002). The acylation status of aACP expressed in *E. coli* may therefore differ from its native modification status in parasites and impact its heterologous association with PKII in bacteria. It is also possible that aACP and PKII associate as part of a larger metabolic complex in the apicoplast, in which additional interacting proteins tune and tighten the ACP-PKII interaction. We noted that several apicoplast-targeted proteins were identified as ACP interactors by both miniTurbo and IP/MS, suggesting additional interactors of ACP and PKII. These proteins include DXS, which catalyzes the first step in isoprenoid precursor synthesis using PKII-generated pyruvate as a substrate, as well as acyl CoA synthetase 5; falcilysin; and chaperones ClpB1, CPN60, and HSP90 (Supplementary file 1 – supplementary table 4). Future biochemical and structural studies can assess the native assembly and potential complex formation of these proteins in parasites.

Two observations support a key role for the 4-PP group in mediating aACP association with PKII. Only WT aCP, but not the Ser95Ala mutant (which lacks 4-PP modification), was pulled down with PKII in parasites by IP/MS and in bacterial expression experiments by Ni-NTA/western blot (Figure 4). We also observed that ACPS, the enzyme that attaches 4-PP to ACP (Figure 1), is essential for parasite viability and apicoplast biogenesis (Figure 3). *P. falciparum* ACPS was previously noted to contain a fused metal hydrolase domain of uncertain function (Cai et al., 2005), and future studies will be required to dissect the contribution of this domain to apicoplast disruption in ∆ACPS parasites. Nevertheless, our results support the model that ACPS is essential for attaching CoA-derived 4-PP to aACP to support critical interaction with PKII. Prior work has shown that the terminal step in CoA synthesis catalyzed by dephospho-CoA kinase (DPCK) occurs in the apicoplast, despite other enzymes in this pathway localizing to the cytoplasm (Swift et al., 2021). The essential role for 4-PP attachment to aACP may provide the evolutionary driving force to localize DPCK for CoA production in the apicoplast.

**Figure 6.**
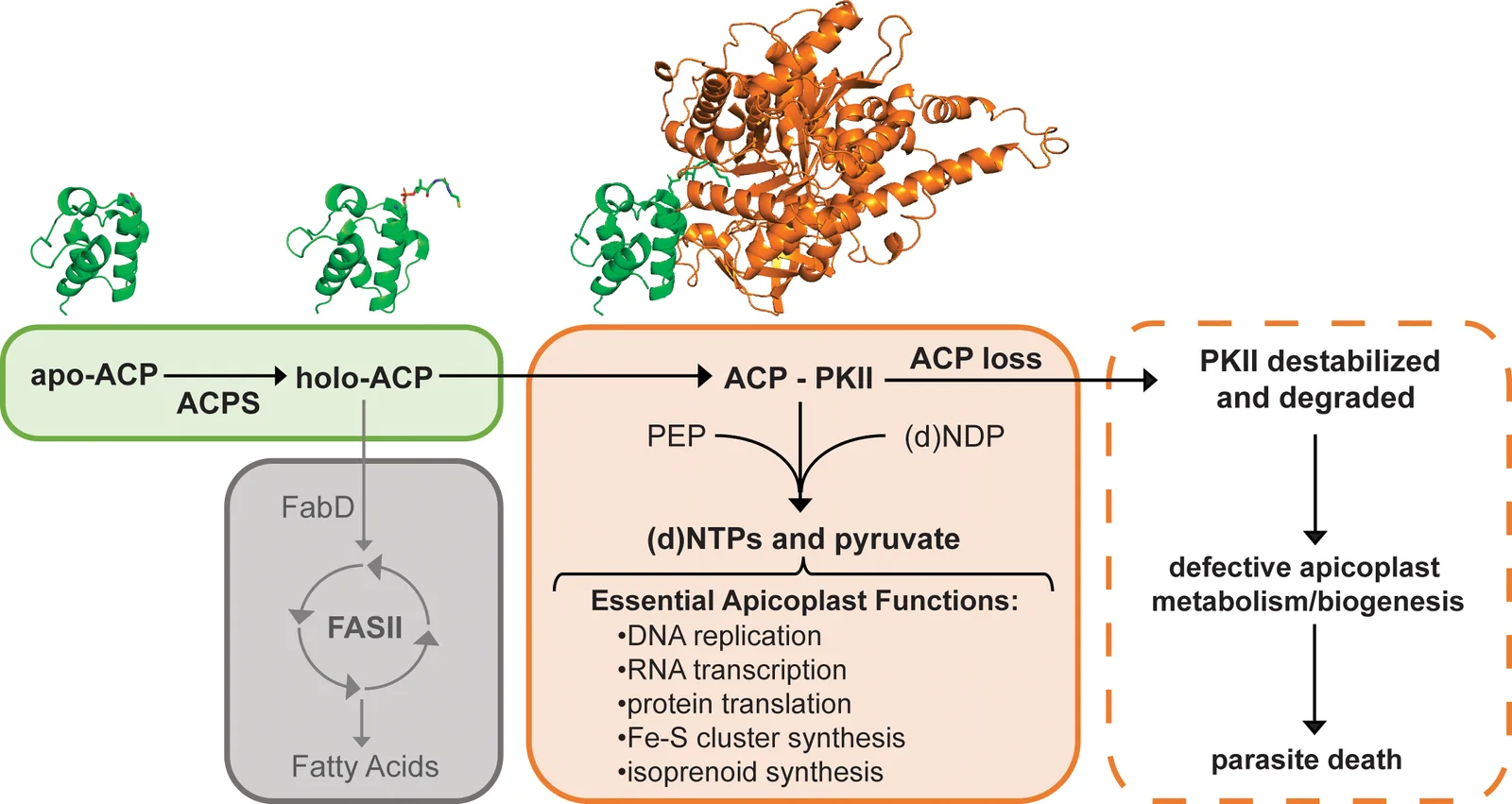
Scheme of acyl carrier protein (ACP) interaction with pyruvate kinase II (PKII) and dysfunctions upon ACP knockdown. Loss of ACP synthase (ACPS) is expected to phenocopy loss of ACP. PEP = phosphoenolpyruvate, (d)NTPs = nucleoside triphosphates and deoxynucleoside triphosphates. The aACP structural model is based on PDB 3GZM and 5USR. The structural model of aACP bound to PKII was generated using AlphaFold 3.

AlphaFold modeling supports direct association of aACP with the central A domain of PKII, with Ser95 and the 4-PP group of ACP positioned at the binding interface and proximal to the expected PKII active site between the A and B domains (Figure 6, Figure 6—figure supplement 1). PK enzymes from distinct organisms are known to be allosterically regulated by various small-molecule metabolites (Schormann et al., 2019). It is possible that aACP binding allosterically modulates PKII activity, but our results suggest that ACP association directly stabilizes PKII such that ACP knockdown or deletion causes PKII destabilization and loss (Figure 6). This stabilizing role for aACP is analogous to similar roles for mACP in binding and stabilizing key mitochondrial complexes via interaction with LYR-motif family proteins (Masud et al., 2019; Van Vranken et al., 2016), including in *P. falciparum* (Falekun et al., 2021). aACP interaction with PKII is not mediated by an LYR sequence, nor do LYR-motif proteins appear to exist in the apicoplast or in plastids (e.g. chloroplasts) more broadly (Dohnálek and Doležal, 2024; Saha et al., 2025; Richards and van der Giezen, 2006). However, bacterial ACPs are known to associate with diverse protein interactors beyond FASII enzymes without evidence for LYR-motif proteins (Gully et al., 2003; Cronan, 2023). The aACP-PKII interaction may resemble bacterial ACP interactions and provides a new molecular paradigm for expanded ACP function in eukaryotes. Future studies can clarify if this interaction is a *Plasmodium*-specific adaptation or general to other plastid-containing cells.

**Figure 6—figure supplement 1.**
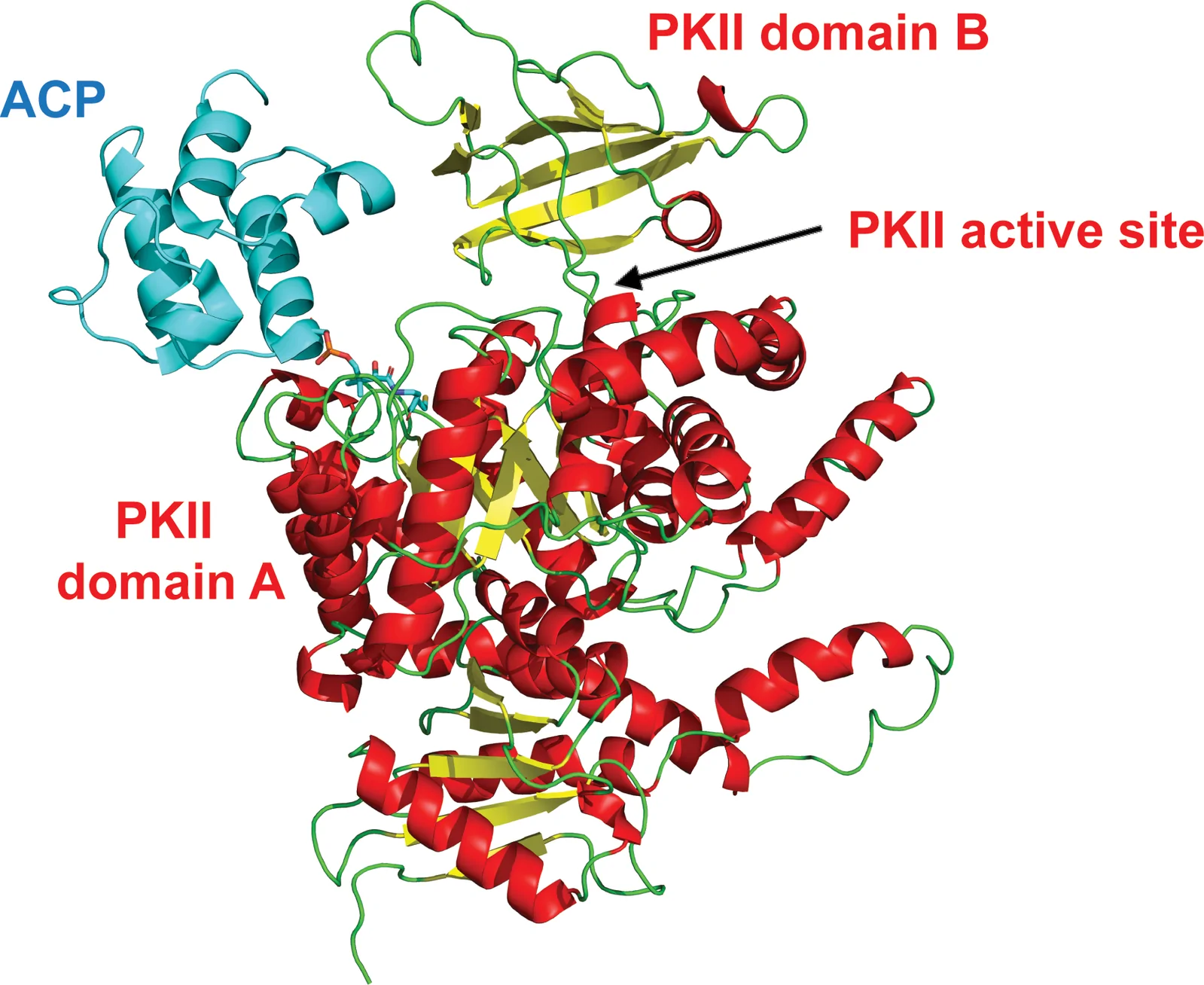
AlphaFold 3 model of apicoplast acyl carrier protein (ACP) bound to pyruvate kinase II (PKII), rendered in PyMOL. The unstructured N-terminus of parasite PKII, which is expected to function as the apicoplast-targeting sequence and be cleaved upon organelle import, was not included in modeling. The presence of the 4-phosphopantetheine group (4-PP) group was modeled by superposition with ACP in the PDB file 5USR.

### Essential ACP function in the apicoplast

The critical stabilizing interaction between aACP and PKII is sufficient to explain essential ACP function in apicoplast biogenesis in *P. falciparum*. It is possible that aACP interactions with other apicoplast proteins, including DXS and falcilysin, also contribute to its essential function (e.g. by regulating their stability and/or activity), but we did not further investigate these interactions. PKII is proposed to be the dominant source of pyruvate, NTPs, and dNTPs required for isoprenoid precursor synthesis via the methylerythritol phosphate pathway, RNA transcription, DNA replication, Fe-S cluster synthesis, and protein translation in the apicoplast (Swift et al., 2020a). Thus, PKII underpins nearly all aspects of apicoplast function, making it a central hub for organelle metabolism and biogenesis.

In eukaryotes with mitochondrial FASII, mACP association with respiratory complexes via LYR-motif proteins depends on the acylation state of mACP and is thought to serve a regulatory role coupling acetyl-CoA availability to assembly and function of the mitochondrial respiratory chain (Van Vranken et al., 2018; Masud et al., 2019; Nowinski et al., 2020). It is intriguing to consider whether apicoplast association of ACP with PKII might similarly depend on aACP modification status and thus serve a regulatory role that modulates PKII levels and broader organelle metabolism under differing growth conditions. The acylation status of aACP and its 4-PP group in blood-stage parasites has not been directly determined. With little to no detectable FASII activity, aACP may predominate in its holo, uncharged form with a free thiol on the 4-PP group (Figure 1), as suggested by prior experiments testing the impact of redox cycling on ACP electrophoretic mobility (Gallagher and Prigge, 2010).

It is possible that a small and variable population of acyl-ACP bearing host-derived fatty acids exists, which can occur if ACPS uses an acyl-CoA (bearing a scavenged fatty acid) to attach an acyl-PP group to aACP (Quadri et al., 1998). *P. falciparum* ACP has also been reported to have self-acylation activity via nucleophilic reaction with an acyl-CoA, although the very low rate constant (*k*_cat_ ~0.1 min^–1^) reported for this activity makes its biological relevance questionable (Misra et al., 2007). We emphasize, however, that future experiments will be required to rigorously determine the modification states of aACP, including tandem mass spectrometry experiments that can enrich, eject, and fragment the 4-PP group to interrogate its structure (Nam et al., 2020). Based on a model in which aACP exists predominantly in the holo, uncharged state in blood-stage parasites, we speculate that an increasing population of acyl-ACP might tighten interaction with PKII and thus increase overall PKII protein stability and activity. Variable ACP acylation and impacts on PKII activity might play a more critical role during parasite growth within mosquitoes or human hepatocytes, when FASII activity is upregulated to increase the pool of acyl-ACP and when parasites are undergoing large-scale proliferation with enhanced reliance on apicoplast biosynthetic functions (van Schaijk et al., 2014; Yu et al., 2008; Buchanan et al., 2022).

### Implications for other apicomplexan parasites

*Toxoplasma gondii*, like other apicomplexan parasites, retains an apicoplast FASII pathway that is dispensable in tachyzoites but enhances their fitness and growth (Liang et al., 2020; Krishnan et al., 2020). Knockdown of aACP in *T. gondii*, however, produces a much more severe phenotype with strong growth impairment (Mazumdar et al., 2006). This observation suggests that aACP may also play additional roles in *Toxoplasma* beyond FASII (Liang et al., 2020), which may also involve stabilizing interactions with diverse organelle proteins. However, apicoplast PKII is dispensable for *Toxoplasma* tachyzoites (Niu et al., 2022), suggesting that the specific interactions that underpin aACP essentiality in *T. gondii* may differ from *P. falciparum*. It remains unclear whether any FASII-independent functions for aACP in *Toxoplasma* depend on 4-PP attachment, but genome-wide CRISPR screens in tachyzoites suggest a critical role for ACPS (Sidik et al., 2016). Prior work in bacteria has suggested that ACPS is a viable therapeutic target (Ballinger et al., 2019; Foley et al., 2009), and inhibiting this essential enzyme may be a potent antimalarial strategy with efficacy against *P. falciparum*, as well as other apicomplexan parasites.

## Materials and methods

**Key resources table:** 

Reagent type (species) or resource	Designation	Source or reference	Identifiers	Additional information
Cell line (*Plasmodium falciparum*)	Dd2	PMID:1970614	BEI Resources MRA-156	
Cell line (*P. falciparum*)	NF54 PfMev	PMID:32059044		Can be obtained from Prigge lab
Cell line (*P. falciparum*)	NF54 PfMev aACP-HA-FLAG aptamer/TetR-DOZI	Made for this study		Can be obtained from Sigala lab
Cell line (*P. falciparum*)	NF54 PfMev aACP knockout	Made for this study		Can be obtained from Prigge lab
Cell line (*P. falciparum*)	NF54 PfMev ACPS knockout	Made for this study		Can be obtained from Prigge lab
Cell line (*P. falciparum*)	NF54 PfMev FabD knockout	Made for this study		Can be obtained from Prigge lab
Cell line (*P. falciparum*)	Dd2 mACP-HA_2_ (pTEOE)	PMID:34612205		Can be obtained from Sigala lab
Cell line (*P. falciparum*)	Dd2 aACP-HA_2_ (pTEOE)	PMID:34612205		Can be obtained from Sigala lab
Cell line (*P. falciparum*)	Dd2 S95A aACP-HA_2_ (pTEOE)	Made for this study		Can be obtained from Sigala lab
Cell line (*P. falciparum*)	Dd2 aACP-BioID2-HA_5_ (pTEOE)	Made for this study		Can be obtained from Sigala lab
Cell line (*P. falciparum*)	Dd2 aACP-MiniTurbo-HA_5_ (pTEOE)	Made for this study		Can be obtained from Sigala lab
Cell line (*P. falciparum*)	Dd2 aACP-HA-FLAG aptamer/TetR-DOZI	Made for this study		Can be obtained from Sigala lab
Cell line (*P. falciparum*)	Dd2 PKII-Myc_3_ (pTEOE) aACP-HA-FLAG aptamer/TetR-DOZI	Made for this study		Can be obtained from Sigala lab
Cell line (*Escherichia coli*)	BL21/DE3 MBP-PKII-HIS_6_ (pMAL) aACP-HA_2_ (pET28a)	Made for this study		Can be obtained from Sigala lab
Cell line (*E. coli*)	BL21/DE3 MBP-PKII-HIS_6_ (pMAL) S95A-aACP-HA_2_ (pET28a)	Made for this study		Can be obtained from Sigala lab
Antibody	Anti-HA (3F10) (rat, monoclonal)	Sigma (Roche)	Cat. No. 11867423001	(1:1000)
Antibody	Anti-aACP (rabbit, polyclonal)	Made for this study		(1:1000) Can be obtained from Sigala lab
Antibody	Anti-His_6_ DyLight680 (mouse, monoclonal)	Thermo Fisher	Cat. No. MA121315D680, RRID:AB_2536987	(1:1000)
Antibody	Anti-EF1α (rabbit, polyclonal)	PMID:11251817		(1:1000)
Antibody	Anti-Myc (mouse, monoclonal)	Invitrogen	Cat. No. MA1-21316	(1:1000)

### Parasite culturing

All experiments were performed using *P. falciparum* Dd2 (Wellems et al., 1990) or NF54 PfMev (Swift et al., 2020b) parasite strains. Parasite strains were confirmed based on expected drug resistance and were *Mycoplasma*-free by PCR. Parasite culturing was performed in Roswell Park Memorial Institute Medium (RPMI-1640, Thermo Fisher 23400021) supplemented with 2.5 g/L Albumax I Lipid-Rich BSA (Thermo Fisher 11020039), 15 mg/L hypoxanthine (Sigma H9636), 110 mg/L sodium pyruvate (Sigma P5280), 1.19 g/L HEPES (Sigma H4034), 2.52 g/L sodium bicarbonate (Sigma S5761), 2 g/L glucose (Sigma G7021), and 10 mg/L gentamicin (Invitrogen Life Technologies 15750060). Cultures were generally maintained at 2% hematocrit in deidentified human erythrocytes obtained from the University of Utah Hospital blood bank, at 37°C, and at 5% O_2_, 5% CO_2_, 90% N_2_.

### Cloning and transfection for episomal expression in *P. falciparum*

The genes encoding aACP (PF3D7_0208500) and mACP (PF3D7_1208300) were cloned into the pTEOE episomal protein expression vector with C-terminal HA_2_ tag as described previously (Falekun et al., 2021). The aACP S95A mutant in the pTEOE vector was made by site-directed mutagenesis of the prior aACP pTEOE plasmid using primers P1/P2 (primer sequences in Supplementary file 1 – supplementary table 5) and confirmed by Sanger sequencing using plasmid-specific primers. For cloning of aACP-BioID2-HA_5_/pTEOE and aACP-MiniTurbo-HA_5_/pTEOE plasmids, aACP was PCR-amplified from the aACP-HA_2_/pTEOE plasmid using primers P3-P5 to yield aACP amplicons with 5’ homology to the pTEOE vector and 3’ homology to BioID2 or miniTurbo. BioID2 and miniTurbo were PCR-amplified from previously published plasmids (Ambekar et al., 2022) using primers P6-P9 to produce amplicons with 5’ homology to aACP and 3’ homology to the HA_5_ tag sequence in a pTEOE vector. The aACP amplicon was combined with either the BioID2 or miniTurbo amplicon in a ligation-independent cloning reaction (NEBuilder HiFi DNA Assembly Master Mix) for insertion into pTEOE. PKII (PF3D7_1037100) was PCR-amplified from Dd2 parasite gDNA via primers P10/P11 and combined with a 3xMyc amplicon created with primers P12/P13 in a ligation-independent cloning reaction for insertion into pTEOE at XhoI/NotI sites to create a PKII-Myc_3_/pTEOE plasmid. Correct clonal plasmid sequences were confirmed by Sanger and/or whole-plasmid sequencing (https://plasmidsaurus.com/).

For episomal expression using the pTEOE vector, Dd2 parasite-infected erythrocytes were transfected in 1× cytomix containing 50–100 µg purified plasmids and 25 µg of the pHTH transposase plasmid (Balu et al., 2005) by electroporation in 0.2 cm cuvettes using a Bio-Rad Gene Pulser Xcell system (0.31 kV, 925 µF). Transfected cultures were allowed to expand in the absence of drug for 48 hr and then selected in 5 nM WR99210 (Jacobus Pharmaceuticals). Stable drug-resistant parasites returned from transfection in 2–8 weeks and were used as polyclonal cultures.

### Cloning and transfection for CRISPR/Cas9 gene editing for aACP knockdown

CRISPR/Cas9-stimulated repair by double-crossover homologous recombination was used to tag the aACP gene (Pf3D7_0208500) to encode a C-terminal HA-FLAG epitope tag fusion and the 3’ 10× aptamer/TetR-DOZI system (Ganesan et al., 2016) to enable regulated aACP expression using aTc (Cayman Chemicals 10009542). A guide RNA (gRNA) sequence of AGACCTCGTTGAATTAATTA corresponding to the sense strand in the third exon of the aACP gene was cloned by ligation-independent methods using primer pairs P14/P15 into a modified version of the pAIO CRISPR/Cas9 vector that contained a HindIII site in place of the BtgZI site. For homology-directed repair to tag the aACP gene, the donor pMG75 repair plasmid was created using ligation-independent cloning to insert a gBlock gene fragment from IDT into the unique AscI/AatII cloning sites. This gBlock contained 153 bp of the 3’ untranslated region of the aACP gene (starting at position 95 downstream from the TAA stop codon), an AfeI site, and the 316 bp of the 3’ end of the aACP coding sequence (excluding the 175 bp intron 2). The gBlock sequence included two underlined shield mutations within the targeted gRNA sequence (AGATCTCGTTGAATTAATCA) that resulted in silent mutations. All cloned sequences were verified by Sanger sequencing. Before transfection, the pMG75 vector was linearized by AfeI digestion performed overnight at 37°C.

Dd2 and PfMev parasites were transfected by electroporation of uninfected erythrocytes with 50 µg each of the linearized pMG75 donor plasmid and pAIO Cas9 plasmid in 1× cytomix using the Bio-Rad Gene Pulser Xcell system (0.31 kV, 950 µF) and 0.2 cm cuvettes. These transfected erythrocytes were then mixed with 1–2% parasitemia synchronous schizont-stage parasites, maintained in 0.5 µM aTc, and allowed to grow for 72 hr prior to drug selection with 6 µM blasticidin-S (Invitrogen R21001). Polyclonal parasites that returned from transfection were genotyped by genomic PCR using primers P16/P17 (WT) and P16/P18 (integrated). Integrated PfMev parasites were fully integrated without evidence for unmodified aACP locus and used as polyclonal parasites in downstream assays. Dd2 parasites were cloned by limiting dilution. Integrated aACP-aptamer/TetR-DOZI Dd2 parasites were transfected by electroporation with 50 µg of the PKII-Myc_3_/pTEOE plasmid as above, selected with 5 nM WR99210 (Jacobus Pharmaceuticals), and used as polyclonal parasites for subsequent experiments.

### Cloning and transfection for knockout of aACP, ACPS, and FabD

The genes encoding aACP (Pf3D7_0208500), holo-ACPS (Pf3D7_0420200), and FabD (Pf3D7_1312000) were disrupted in the NF54 PfMev line using CRISPR/Cas9 and gene deletion by double-crossover homologous recombination. Homology arm regions were PCR-amplified from genomic DNA with primers P19-P22 (aACP: 421 bp 5’ arm, 362 bp 3’ arm), P23-P26 (ACPS: 416 bp 5’ arm, 426 bp 3’ arm), and P27-P30 (FabD: 346 bp 5’ arm, 402 bp 3’ arm) and cloned into vector pRS (Swift et al., 2020b) using ligation-independent cloning (Clontech In-Fusion). DNA encoding gRNA with sequences AATGAACTCAAATTTTACCA (aACP), TAAAATATTGAACCCACGAG (ACPS), and GATAGAAAATTTGGTTTATG (FabD) were cloned into a modified pAIO vector called pCasG (Rajaram et al., 2020) using primers P31/P32, P33/P34, P35/P36, respectively.

For transfection, 75 µg of each pRS plasmid was combined with 75 µg of the matching pCasG plasmid and electroporated into NF54 PfMev parasites. Transfected parasites were allowed to expand for 48 hr in 50 µM mevalonate before selection with 5 nM WR99210 and 50 µM DL-mevalonolactone (Cayman Chemicals 20348, referred to as mevalonate in this work). Parasites returning from positive selection were genotyped by PCR using primers P37-P40 (aACP), P41-P44 (ACPS), P45-P48 (FabD) in conjunction with primers P49-50 to confirm complete on-target integration in polyclonal cultures. A primer scheme and expected amplicon sizes are provided in Supplementary file 2. Apicoplast (SufB: Pf3D7_API04700), mitochondrion (CoxI, Pf3D7_MIT02100), and nuclear (LDH, Pf3D7_1324900) genome PCR was performed using primers P51-P56 to confirm apicoplast status in knockout parasites.

### Cloning for recombinant expression in *E. coli*

The pMAL expression vector encoding codon-harmonized mature (AA42–745) PKII with N-terminal maltose binding protein (MBP) domain, TEV protease site, and His_6_ tag was cloned as described previously (Swift et al., 2020a). This plasmid was co-transformed with either a WT (∆2–40) aACP-HA_2_ pET28a plasmid published previously (Falekun et al., 2021) or a S95A (∆2–40) aACP-HA_2_ pET28a plasmid prepared from the WT aACP plasmid by site-directed mutagenesis using primers P1/P2 and confirmed by Sanger sequencing.

### Recombinant expression in *E. coli*

Bacterial expression experiments were performed as previously described (Falekun et al., 2021). Plasmids for co-expression of His_6_-PKII with either WT or S95A aACP-HA_2_ were transformed into chemically competent BL21/DE3 cells and selected with kanamycin (50 µg/mL) and carbenicillin (100 µg/mL) for inducible expression with 1 mM isopropyl 1-thio-β-galactopyranoside (IPTG) (Goldbio 367931) in 20 mL LB media. Cells were grown at 37°C until reaching an optical density (600 nm) of 0.6, induced with IPTG, and grown overnight at 20°C before harvest by centrifugation. Bacterial pellets were resuspended in 1 mL cold phosphate-buffered saline (PBS, pH 7.4), lysed by sonication on ice using a Branson sonicator equipped with a microtip, and clarified by centrifugation at 17,000×*g* in a Fisher Scientific accuSpin 17R microcentrifuge. Clarified lysates were treated with recombinant TEV protease overnight at 4°C to cleave off the MBP tag and release His_6_-PKII. Treated lysates were then incubated with 100 µL of Ni-NTA resin (Thermo Scientific 88221), after resin equilibration with buffer A (50 mM NaH_2_PO_4_, 500 mM NaCl, 5 mM imidazole, pH 8.0) to bind His_6_-PKII and any co-associating proteins. Resin was collected by centrifugation, washed three times with buffer A, and eluted by incubation with 100 µL buffer B (buffer A+500 mM imidazole) for 15 min. on ice. Eluates were diluted into SDS sample buffer and further analyzed by SDS-PAGE and western blot.

### Parasite growth assays

Parasites were synchronized with 5% (wt/vol) D-sorbitol for 10 min at room temperature, washed 3× with drug-free RPMI medium, and then seeded for growth assays at an initial ring parasitemia of ~0.25% with daily media changes and parasitemia measurements. For tests of regulatable aACP expression, Dd2 aACP-HA-Flag 10× Aptamer/TetR-DOZI parasites were split into biological triplicate populations either grown without aTc (–aTc), with 200 µM IPP (Isoprenoids IPP001) added (–aTc, +IPP), or with 0.5 µM aTc (+aTc). Parasitemia was measured daily in technical duplicate 10 µL samples, diluted into 200 µL of 1 µg/mL acridine orange (Invitrogen Life Technologies A3568) in PBS and analyzed by flow cytometry using a BD FACSCelesta system monitoring SSC-A, FSC-A, PE-A, FITC-A, and PerCP-Cy5-5-A channels. Averages ± SD were determined and plotted using GraphPad Prism 9.0.

Synchronous growth assays of ∆ACP, ∆ACPS, and ∆FabD PfMev parasites were performed similarly, with growth monitored ±50 µM mevalonate in cultures. For growth assays in low-lipid conditions, parasites and RBCs were washed into modified complete RPMI medium in which Albumax I was replaced with fatty acid-free bovine serum albumin (GoldBio A-421-10) supplemented with 45 µM oleic (Sigma O1008) and 30 µM palmitic (Sigma 57-10-3) acids with daily media changes. Parasites were cultured ≥1 week in low-lipid conditions prior to initiating synchronous growth assays, which were completed in biological triplicate samples.

### Production and validation of custom anti-aACP antibody

The sequence encoding mature, processed (∆1–40) aACP was cloned into pET28 with an N-terminal His-tag, expressed in *E. coli*, purified by Ni-NTA, cleaved by thrombin, and purified by FPLC using previously reported methods (Falekun et al., 2021). Purified protein was then injected into a rabbit for polyclonal antibody production by Cocalico Biologicals Inc (https://cocalicobiologicals.com) following their standard protocol. Rabbit serum was validated for specific detection of aACP with recombinant protein expressed in bacteria (Figure 2—figure supplement 5).

### Fluorescence microscopy

Live-parasite microscopy and immunofluorescence assays (IFA) with fixed parasites were performed as previously described (Okada et al., 2022). For live-cell experiments, parasite nuclei were visualized by incubation with 1–2 µg/mL Hoechst 33342 (Pierce 62249) for 10–20 min at room temperature. The parasite apicoplast was visualized in live PfMev parasites using the endogenous fluorescence of ACP_L_-GFP expressed in this line (Swift et al., 2020b). For IFA experiments, parasites were fixed, stained, and mounted as described previously (Blackwell et al., 2024; Tonkin et al., 2004). The nucleus was visualized in IFA samples using ProLong Gold Antifade Mountant with DAPI (Invitrogen P36931). The apicoplast was visualized using polyclonal rabbit anti-aACP antibodies either previously published (Gallagher and Prigge, 2010) or produced for this study and a goat anti-rabbit 2° antibody (Invitrogen A32731). Images of aACP knockdown parasites (Dd2 and PfMev) were taken on an EVOS M5000 imaging system, with processing done in Fiji/ImageJ. Images of ∆ACPS, ∆ACP, and ∆FabD parasites were acquired on a Zeiss Axioimager M2 microscope, with processing done with VOLOCITY (PerkinElmer). All image processing was done on a linear scale. Scale bars are ~1 µm.

### SDS-PAGE and western blots

For analysis of *P. falciparum* samples, blood-stage parasites were treated with 0.05% saponin (Sigma 84510) in PBS for 5 min at room temperature and harvested by centrifugation. Saponin pellets were then lysed by resuspension in 1% Triton X-100 in PBS after addition of protease inhibitors (Invitrogen A32955) and sonicated ~20 pulses (50% duty cycle, 30–40% power) on a Branson microtip sonicator. Parasite lysates were clarified by centrifugation at 17,000×*g* for 10 min. Bacterial samples were harvested and lysed as described above. Protein concentrations in lysates were quantified by Lowry colorimetry, and 50 µg of total protein from each sample was mixed with SDS sample buffer and heated at 95°C for 10 min. Samples were loaded and fractionated by 10–16% SDS-PAGE in Tris-HCl buffer, transferred to nitrocellulose membrane using the Bio-Rad wet-transfer system for 1 hr at 100 V, and blocked with 5% non-fat milk in TBS-T (50 mM Tris pH 7.4, 150 µM NaCl, 0.5% Tween-20). Membranes were probed with primary antibodies: rat anti-HA-tag monoclonal 3F10 (Roche), rabbit anti-EF1α or rabbit anti-EF1β (Mamoun and Goldberg, 2001), mouse anti-Myc-tag monoclonal (Invitrogen MA1-21316), and/or mouse anti-His-tag monoclonal-HRP (QIAGEN 34460). Membranes were washed 3× in TBS-T buffer and probed with secondary antibodies: donkey anti-rabbit IRDye680 (LI-COR 926-68023), goat anti-rat IRDye800CW (LI-COR 926-32219), goat anti-rat-HRP (Invitrogen 62-9520), or goat anti-mouse-HRP (Invitrogen A28177). Membranes were imaged on a LI-COR Odyssey CLx system or Invitrogen iBright 1500 imager. Band intensities were quantified by densitometry using ImageJ and analyzed using GraphPad Prism 9.0.

### Measuring DNA and RNA abundance in parasites by PCR

aACP knockdown Dd2 or PfMev parasites were synchronized with 5% D-sorbitol and cultured +aTc, –aTc/+IPP (Dd2), or –aTc/+Mev (PfMev) for 36 or 84 hr prior to harvest for DNA and RNA extraction, as previously reported (Blackwell et al., 2024). Parasite DNA was extracted using a QIAamp DNA Blood Mini Kit (QIAGEN 51106). Parasite RNA was purified by TRIzol (Invitrogen 15596026) and phenol-chloroform extraction. RNA was reverse-transcribed to cDNA using a SuperScript IV VILO RT Kit (Invitrogen 11766050) using gene-specific primers, as published previously (Blackwell et al., 2024). Invitrogen Quantstudio Real-Time PCR systems were used to quantify abundance of DNA and cDNA using SYBR Green dye. The relative DNA or cDNA abundance of each apicoplast gene was normalized to the average of three nuclear-encoded genes for each sample, and –aTc conditions were compared to +aTc conditions at each time point by the comparative Ct method (Schmittgen and Livak, 2008).

All qPCR experiments were performed in biological triplicate, with each sample analyzed in technical duplicate, and data were analyzed by unpaired Student’s t-test in GraphPad Prism 9.0. PCR analysis of apicoplast, mitochondrial, and nuclear genome status was performed in ∆ACP, ∆ACPS, and ∆FabD PfMev parasites, as previously reported (Swift et al., 2020b).

### Immunoprecipitations

IP experiments for mass spectrometry analysis were performed for Dd2 parasites episomally expressing WT or S95A aACP-HA_2_ or WT mACP-HA_2_, as previously described (Falekun et al., 2021). Blood-stage parasites from ~50 mL cultures were harvested by centrifugation, treated with 0.05% saponin (Sigma 84510) in PBS for 5 min at room temperature, and clarified by centrifugation at 3000×*g* for 30 min at 4°C. IP was performed using Pierce anti-HA magnetic beads (Thermo Scientific 88836). Parasite pellets were lysed in 1 mL cold 1% Triton (Sigma 9002931)/PBS, 1% digitonin (GoldBio 11024-24-1)/PBS, or RIPA buffer (150 mM NaCl, 1% Triton X-100, 0.5% deoxycholate, 0.1% SDS, 50 mM Tris, pH 8.0) plus protease inhibitor (Thermo Scientific A32955). Pellets were dispersed by brief sonication on a Branson sonicator equipped with a microtip probe and then incubated at 4°C for 1 hr on a rotator. Lysates were clarified by centrifugation at 17,000×*g* for 10 min. 30 μL of anti-HA magnetic beads was equilibrated in 170 μL of cold 1× Tris-buffered saline (20 mM Tris, pH 7.6, 150 mM NaCl)+0.05% Tween-20 (TBS-T), and the beads were collected on a magnetic stand. Parasite lysates supernatants (1 mL) were added to the equilibrated beads and incubated for 1 hr at 4°C by rotation. Beads were washed three times with ice-cold 1× TBS-T and transferred to a new tube. Bound proteins were eluted with 100 μL 8 M urea (in 100 mM Tris at pH 8.8) and precipitated by adding 100% trichloroacetic acid (Sigma 76039) to a final concentration of 20%. Samples were incubated on ice for 1 hr and centrifuged at 17,000×*g* for 25 min at 4°C. Supernatants were removed by vacuum aspiration, and protein pellets were washed once with 500 μL of cold acetone. The protein pellets were air-dried for 30 min and stored at –20°C.

### Proximity biotinylation experiments

For proximity biotinylation experiments with Dd2 parasites episomally expressing aACP-BioID2 or aACP-miniTurbo, 50 mL cultures were synchronized with 5% D-sorbitol and grown to ~15% parasitemia before treatment with 50 µM biotin (Sigma 58-85-5) for 24 hr before harvesting by centrifugation and saponin treatment. Parental Dd2 parasites were used as negative control and treated identically. Parasite pellets were lysed in 1 mL 1% Triton X-100/PBS and clarified by centrifugation at 17,000×*g*. Clarified lysates were added to 25 µL of M-280 streptavidin magnetic beads (Invitrogen 11205D) that had been equilibrated in PBS, incubated overnight with rotating at 4°C, and washed 5× with 1 mL 1% Triton/PBS, 4× with 1 mL 8 M urea/PBS, and 2× in PBS, ensuring that samples were moved to new tubes between each wash condition. The final washed beads were resuspended in 50 µL PBS and stored at 4°C briefly before shipment for on-bead tryptic digest and analysis by tandem mass spectrometry. 5 µL of the final suspended beads were diluted into SDS sample buffer, boiled at 95°C for 10 min, fractionated by 12% SDS-PAGE, transferred to nitrocellulose, and probed with streptavidin-AF680 conjugate (Invitrogen S32358).

### Mass spectrometry

Protein samples were reduced and alkylated using 5 mM Tris (2-carboxyethyl) phosphine and 10 mM iodoacetamide, respectively, and then enzymatically digested by sequential addition of trypsin and Lys-C proteases, as previously described (Wohlschlegel, 2009). The digested peptides were desalted using Pierce C18 tips (Thermo Fisher Scientific), dried, and resuspended in 5% formic acid. Approximately 1 μg of digested peptides was loaded onto a 25-cm-long, 75 μm inner diameter fused silica capillary packed in-house with bulk ReproSil-Pur 120 C18-AQ particles, as described previously (Jami-Alahmadi et al., 2021). The 140 min water-acetonitrile gradient was delivered using a Dionex Ultimate 3000 ultra-high performance liquid chromatography system (Thermo Fisher Scientific) at a flow rate of 200 nL/min (buffer A: water with 3% DMSO and 0.1% formic acid, and buffer B: acetonitrile with 3% DMSO and 0.1% formic acid). Eluted peptides were ionized by the application of distal 2.2 kV and introduced into the Orbitrap Fusion Lumos mass spectrometer (Thermo Fisher Scientific) and analyzed by tandem mass spectrometry. Data were acquired using a Data-Dependent Acquisition method consisting of a full MS1 scan (resolution = 120,000) followed by sequential MS2 scans (resolution = 15,000) for the remainder of the 3 s cycle time. Data were analyzed using the Integrated Proteomics Pipeline 2 (Integrated Proteomics Applications, San Diego, CA, USA). Data were searched against the protein database from *P. falciparum* 3D7 downloaded from UniprotKB (10,826 entries) on October 2013. Tandem mass spectrometry spectra were searched using the ProLuCID algorithm followed by filtering of peptide-to-spectrum matches by DTASelect using a decoy database-estimated false discovery rate of <1%. The proteomics data are deposited in the MassIVE data repository (https://massive.ucsd.edu) under the identifier MSV000100931. Protein enrichment values were calculated as the log_2_ ratio of spectral counts or intensity values for the indicated protein in the experimental versus negative-control datasets (mini-Turbo: Dd2, BioID2: Dd2, aACP: mACP, WT aACP: S95A ACP). Protein spectral count values of zero were arbitrarily converted to one for purposes of calculating a log_2_ enrichment ratio.

### Structural modeling

A structural model of mature *P. falciparum* aACP bound to mature PKII was generated using AlphaFold 3 (Abramson et al., 2024) and visualized using PyMOL 3.1 (MacPyMOL, 2007).

### Materials availability

All materials created during this study can be obtained by contacting the Sigala or Prigge labs.

## Data Availability

The proteomics data are deposited in the MassIVE data repository (https://massive.ucsd.edu) under the identifier MSV000100931. All data generated or analyzed during this study are included in the manuscript and supporting files. Source data files have been provided for Figures 2—5. Figure 4—source data 2 contains the full list of proteins identified by mass spectrometry experiments. Supplementary file 1 contains supplementary tables 1–4 (apicoplast-targeted proteins identified in mass spectrometry experiments) and supplementary table 5 (PCR primer sequences). Supplementary file 2 shows the primer scheme and amplicon sizes for PCR analyses of gene deletions. The following dataset was generated: WohlschlegelJ 2026Affinity purification of acyl carrier protein from PlasmodiumMassIVE10.25345/C5GT5FV51

## Additional files

Supplementary file 1. Supplementary tables 1–5. Supplementary table 1. Table of apicoplast-targeted proteins identified in parasites expressing acyl carrier protein (ACP)-miniTurbo or ACP-BioID2. Supplementary table 2. Table of apicoplast-targeted proteins identified by immunoprecipitation/tandem mass spectrometry (IP/MS) of apicoplast acyl carrier protein (ACP)-HA_2_. Supplementary table 3. Table of apicoplast-targeted proteins identified by immunoprecipitation/tandem mass spectrometry (IP/MS) of wild-type (WT) or Ser95Ala acyl carrier protein (ACP)-HA_2_. Supplementary table 4. Table of apicoplast-targeted protein interactors of apicoplast ACP (aACP) identified by both miniTurbo and immunoprecipitation/tandem mass spectrometry (IP/MS). Supplementary table 5. List of PCR primers.

Supplementary file 2. Scheme and primers for genomic PCR analyses to confirm gene disruptions.

MDAR checklist
