# Supplementary material for: Acyl carrier protein is essential for apicoplast biogenesis in malaria parasites independent of fatty acid synthesis
Source: eLife. 2026 Sep 14;15:RP111494. doi: 10.7554/eLife.111494 (PMC13574338; doi:10.7554/eLife.111494)
Supplement: Supplementary file 2 [file elife-111494-supp1.pdf]

Supplementary File 1

Supplementary Table 1

Apicoplast-targeted proteins identified in parasites expressing ACP-miniTurbo or ACP-BioID2

				Enrichment Ratio	
Plot ID	Gene ID	Annotation	Gene Name	MiniTurbo	BioID2
PKII	PF3D7_1037100	pyruvate kinase 2	PKII	6.672	2.000
1	PF3D7_0816600	chaperone protein ClpB1	ClpB1	6.304	0.000
2	PF3D7_0520800	conserved Plasmodium protein, unknown function	N/A	6.087	0.000
3	PF3D7_1443900	heat shock protein 90, putative	HSP90	5.883	0.000
4	PF3D7_1025300	conserved Plasmodium protein, unknown function	N/A	5.492	0.000
5	PF3D7_1232100	60 kDa chaperonin	CPN60	5.087	4.459
6	PF3D7_1406600	ATP-dependent Clp protease regulatory subunit ClpC, putative	ClpC	4.954	0.000
7	PF3D7_1440200	stromal-processing peptidase, putative	SPP	4.700	0.000
8	PF3D7_0526700	conserved protein, unknown function	N/A	4.585	0.000
9	PF3D7_1469600	biotin carboxylase subunit of acetyl CoA carboxylase, putative	ACC	4.585	0.000
10	PF3D7_1103400	FeS cluster assembly protein SufD, putative	SufD	4.248	0.000
11	PF3D7_0521400	conserved Plasmodium protein, unknown function	N/A	4.170	0.000
12	PF3D7_1123500	conserved Plasmodium protein, unknown function	N/A	4.087	0.000
13	PF3D7_1106500	conserved Plasmodium protein, unknown function	N/A	3.907	0.000
14	PF3D7_0508800	single-stranded DNA-binding protein	SSB	3.585	0.000
15	PF3D7_1411400	plastid replication-repair enzyme	PREX	3.322	0.000
16	PF3D7_1358000	patatin-like phospholipase, putative	PNPLA2	3.322	0.000
17	PF3D7_0316400	conserved Plasmodium protein, unknown function	N/A	3.322	0.000
18	PF3D7_1360800	falcilysin	Falcilysin	3.170	0.000
19	PF3D7_1338600	conserved Plasmodium protein, unknown function	N/A	2.807	0.000
20	PF3D7_1306200	conserved Plasmodium protein, unknown function	N/A	2.807	0.000
21	PF3D7_1229600	conserved Plasmodium protein, unknown function	N/A	2.807	0.000
22	PF3D7_1333000	20 kDa chaperonin	CPN20	2.807	0.000
23	PF3D7_1406400	pentatricopeptide repeat domain-containing protein, putative	PPR1	2.585	0.000
24	PF3D7_1352000	conserved Plasmodium protein, unknown function	N/A	2.585	0.000
25	PF3D7_0731600	acyl-CoA synthetase	ACS5	2.585	0.000
26	PF3D7_0916200	conserved Plasmodium protein, unknown function	N/A	2.322	0.000
27	PF3D7_0607900	conserved Plasmodium protein, unknown function	N/A	2.322	0.000
28	PF3D7_1413500	FeS assembly ATPase SufC	SufC	2.000	0.000
29	PF3D7_1472700	DNA-directed RNA polymerase, alpha subunit, putative	N/A	2.000	0.000
30	PF3D7_1223300	DNA gyrase subunit A	GyrA	2.000	0.000
31	PF3D7_1427000	conserved Plasmodium protein, unknown function	N/A	2.000	0.000
32	PF3D7_1323600	conserved Plasmodium protein, unknown function	N/A	2.000	0.000
33	PF3D7_0313800	conserved Plasmodium protein, unknown function	N/A	2.000	0.000

34	PF3D7_0504400	ATP-dependent helicase, putative	N/A	2.000	0.000
35	PF3D7_0711000	AAA family ATPase, CDC48 subfamily	CDC48	2.000	0.000
36	PF3D7_0518100	RAP protein, putative	AMR2	1.585	0.000
37	PF3D7_0727500	mTERF domain-containing protein, putative	N/A	1.585	0.000
38	PF3D7_1225100	isoleucine--tRNA ligase, putative	api-IRS	1.585	0.000
39	PF3D7_0706100	conserved Plasmodium protein, unknown function	N/A	1.585	0.000
40	PF3D7_0505700	conserved Plasmodium membrane protein, unknown function	N/A	1.585	0.000
41	PF3D7_0904700	bacterial histone-like protein	HU	1.585	0.000
42	PF3D7_1429100	apicoplast ribosomal protein L15 precursor, putative	N/A	1.585	0.000
43	PF3D7_1337200	1-deoxy-D-xylulose 5-phosphate synthase	DXS	1.585	0.000
44	PF3D7_0804400	methionine aminopeptidase 1c, putative	METAP1c	1.000	0.000
45	PF3D7_1307600	DNA-directed RNA polymerase alpha chain, putative	rpoA	1.000	0.000
46	PF3D7_0920200	CS domain protein, putative	N/A	1.000	0.000
47	PF3D7_1470800	conserved Plasmodium protein, unknown function	N/A	1.000	0.000
48	PF3D7_1369600	conserved Plasmodium protein, unknown function	N/A	1.000	0.000
49	PF3D7_0827600	conserved Plasmodium protein, unknown function	N/A	1.000	0.000
50	PF3D7_0505400	conserved Plasmodium protein, unknown function	N/A	1.000	0.000
51	PF3D7_0509600	asparagine--tRNA ligase	AsnRS	1.000	0.000
52	PF3D7_0827500	apicoplast ribosomal protein L21 precursor, putative	N/A	1.000	0.000
53	PF3D7_1022800	4-hydroxy-3-methylbut-2-en-1-yl diphosphate synthase	ISPG	1.000	0.000

Supplementary Table 2

Apicoplast-targeted proteins identified by IP/MS of apicoplast versus mitochondrial ACP-HA_2

				aACP/mACP Enrichment Ratio	
**Plot ID**	**Gene ID**	**Annotation**	**Gene Name**	**RIPA**	**Urea**
aACP	PF3D7_0208500	acyl carrier protein	aACP	3.585	4.644
PKII	PF3D7_1037100	pyruvate kinase 2	PKII	1.585	3.585
1	PF3D7_1126000	threonine--tRNA ligase	ThrRS	2.807	2.322
2	PF3D7_0731600	acyl-CoA synthetase	ACS5	2.000	0.515
3	PF3D7_0912400	alkaline phosphatase, putative	Alkaline Phosphatase	2.000	0.000
4	PF3D7_1123500	conserved Plasmodium protein, unknown function	N/A	2.000	0.000
5	PF3D7_1410700	conserved Plasmodium protein, unknown function	N/A	1.585	0.415
6	PF3D7_1232100	60 kDa chaperonin	CPN60	1.170	0.670
7	PF3D7_1360800	falcilysin	FLN	1.138	1.184
8	PF3D7_1420400	glycine--tRNA ligase	GlyRS	1.000	2.322
9	PF3D7_0816600	chaperone protein ClpB1	ClpB1	1.000	1.585
10	PF3D7_0627700	transportin	Transportin	1.000	1.585
11	PF3D7_1212000	glutathione peroxidase-like thioredoxin peroxidase	TPx(Gl)	1.000	0.000
12	PF3D7_1367700	alanine--tRNA ligase	AlaRS	0.000	3.322
13	PF3D7_1025300	conserved Plasmodium protein, unknown function	N/A	0.000	3.322
14	PF3D7_0111500	UMP-CMP kinase, putative	UMP-CMP Kinase	0.000	3.000
15	PF3D7_1437200	ribonucleoside-diphosphate reductase large subunit, putative	RNR-LSU	0.000	2.807
16	PF3D7_0529000	conserved Plasmodium protein, unknown function	N/A	0.000	2.807
17	PF3D7_0623500	superoxide dismutase [Fe]	SOD2	0.000	2.322
18	PF3D7_1457300	conserved Plasmodium protein, unknown function	N/A	0.000	1.807
19	PF3D7_1443900	heat shock protein 90, putative	HSP90	0.000	1.585
20	PF3D7_1337200	1-deoxy-D-xylulose 5-phosphate synthase	DXS	0.000	1.585
21	PF3D7_1205700	targeted glyoxalase II	tGLO2	0.000	1.585
22	PF3D7_0520800	conserved Plasmodium protein, unknown function	N/A	0.000	1.585
23	PF3D7_1119000	acyl-CoA-binding protein, putative	Acyl-CoA-binding protein	0.000	1.000
24	PF3D7_0505700	conserved Plasmodium membrane protein, unknown function	N/A	0.000	1.000
25	PF3D7_0721100	conserved Plasmodium protein, unknown function	N/A	0.000	0.678
26	PF3D7_0209300	2C-methyl-D-erythritol 2,4-cyclodiphosphate synthase	IspF	0.000	0.585

Supplementary Table 3

Apicoplast-targeted proteins identified by IP/MS of WT or Ser95Ala ACP-HA_2

Plot ID	Gene ID	Annotation	Gene Name	aACP/S95A Enrichment Ratio	
				Digitonin	RIPA
aACP	PF3D7_0208500	acyl carrier protein	aACP	0.585	0.585
PKII	PF3D7_1037100	pyruvate kinase 2	PKII	3.000	1.585
1	PF3D7_1307600	DNA-directed RNA polymerase alpha chain, putative	rpoA	3.907	0.000
2	PF3D7_1338600	conserved Plasmodium protein, unknown function	N/A	3.700	0.000
3	PF3D7_1360800	falcilysin	FLN	3.585	1.874
4	PF3D7_1364600	aldehyde reductase, putative	Aldehyde reductase	3.170	0.000
5	PF3D7_0820800	conserved Plasmodium protein, unknown function	N/A	3.170	0.000
6	PF3D7_0920600	conserved Plasmodium protein, unknown function	N/A	3.170	0.000
7	PF3D7_1025300	conserved Plasmodium protein, unknown function	N/A	3.170	0.000
8	PF3D7_1005900	conserved Plasmodium protein, unknown function	N/A	3.000	0.000
9	PF3D7_1228700	conserved Plasmodium protein, unknown function	N/A	2.700	0.000
10	PF3D7_1212000	glutathione peroxidase-like thioredoxin peroxidase	TPx(Gl)	2.585	2.000
11	PF3D7_0912400	alkaline phosphatase, putative	Alkaline Phosphatase	2.322	2.000
12	PF3D7_0313700	conserved Plasmodium protein, unknown function, unspecified product	N/A	2.322	0.000
13	PF3D7_1021000	conserved Plasmodium protein, unknown function	N/A	2.322	0.000
14	PF3D7_1410700	conserved Plasmodium protein, unknown function	N/A	2.000	1.585
15	PF3D7_1126000	threonine--tRNA ligase	ThrRS	2.000	1.222
16	PF3D7_1443900	heat shock protein 90, putative	HSP90	2.000	1.000
17	PF3D7_0731600	acyl-CoA synthetase	ACS5	2.000	0.000
18	PF3D7_1221800	conserved Plasmodium protein, unknown function	N/A	2.000	0.000
19	PF3D7_1306200	conserved Plasmodium protein, unknown function	N/A	2.000	0.000
20	PF3D7_1323600	conserved Plasmodium protein, unknown function	N/A	2.000	0.000
21	PF3D7_1457300	conserved Plasmodium protein, unknown function	N/A	2.000	0.000
22	PF3D7_1232100	60 kDa chaperonin	CPN60	1.766	2.170
23	PF3D7_0816600	chaperone protein ClpB1	ClpB1	1.766	0.678
24	PF3D7_1420400	glycine--tRNA ligase	GlyRS	1.585	1.000
25	PF3D7_0627700	transportin	Transportin	1.585	1.000
26	PF3D7_1123500	conserved Plasmodium protein, unknown function	N/A	1.585	1.000
27	PF3D7_1472700	DNA-directed RNA polymerase, alpha subunit, putative	N/A	1.585	0.000
28	PF3D7_1440200	stromal-processing peptidase, putative	SPP	1.585	0.000
29	PF3D7_1419200	thioredoxin-like protein, putative	ATrx1	1.585	0.000
30	PF3D7_1367700	alanine--tRNA ligase	AlaRS	1.585	0.000
31	PF3D7_0405400	pre-mRNA-processing-splicing factor 8, putative	PRPF8	1.585	0.000
32	PF3D7_0721100	conserved Plasmodium protein, unknown function	N/A	1.585	0.000

33	PF3D7_1226900	conserved Plasmodium protein, unknown function	N/A	1.585	0.000
34	PF3D7_1409100	aldo-keto reductase, putative	Aldo-Keto Reductase	1.000	0.000
35	PF3D7_1205700	targeted glyoxalase II	tGLO2	1.000	0.000
36	PF3D7_1015200	cysteine--tRNA ligase	CysRS	1.000	0.000
37	PF3D7_0711000	AAA family ATPase, CDC48 subfamily	CDC48	1.000	0.000
38	PF3D7_0530200	phosphoenolpyruvate/phosphate translocator	PPT	1.000	0.000
39	PF3D7_0504400	ATP-dependent helicase, putative	ATP-dependent Helicase	1.000	0.000
40	PF3D7_0111500	UMP-CMP kinase, putative	UMP-CMP kinase	1.000	0.000
41	PF3D7_0624400	conserved Plasmodium protein, unknown function	N/A	1.000	0.000
42	PF3D7_1125000	conserved Plasmodium protein, unknown function	N/A	1.000	0.000
43	PF3D7_0209300	2C-methyl-D-erythritol 2,4-cyclodiphosphate synthase	IspF	0.585	1.585

Supplementary Table 4

Apicoplast-targeted protein interactors of aACP identified by both miniTurbo and IP/MS

(common to Supplementary Tables 1 and 2)

No.	Gene ID	Annotation	Gene Name
1	PF3D7_1232100	60 kDa chaperonin	CPN60
2	PF3D7_1025300	conserved protein, unknown function	N/A
3	PF3D7_1443900	heat shock protein 90, putative	HSP90
4	PF3D7_0505700	conserved Plasmodium membrane protein, unknown function	N/A
5	PF3D7_0816600	chaperone protein ClpB1	ClpB1
6	PF3D7_0731600	acyl-CoA synthetase	ACS5
7	PF3D7_1337200	1-deoxy-D-xylulose 5-phosphate synthase	DXS
8	PF3D7_0520800	conserved protein, unknown function	N/A
9	PF3D7_1360800	falcilysin	FLN
10	PF3D7_1037100	pyruvate kinase 2	PKII
11	PF3D7_1123500	golgi protein 2	N/A

Supplementary Table 5

Primers sequences used for cloning and PCR analyses

Prime	Name	Sequence
P1	aACP_S95A_F	GGGGGCTGATGCTTTAGACCTCGTTG
P2	aACP_S95A_R	CAACGAGGTCTAAAGCATCAGCCCCC
P3	aACP_TEOE_F	aaacacgatttttctcgagATGAAGATCTTATTACTTTG
P4	aACP_BioID2_R	AAtcctccacttcccctaggTTGCTTATTATTTTTTTCTATATAA
P5	aACP_miniTurbo_	ATtcctccacttcccctaggTTGCTTATTATTTTTTTCTATATAA
P6	BioID2_aACP_F	ACTAAACATTTCGAAAAAGTAAAATCAcctaggggaagtggaggaTTCAAGAACCTGATC
P7	BioID2_HA5_R	GATAAGCGTAATCTGGAACATCGTATGGGTAGGCATAATCTGGAACATCGTAAGGATACG
P8	miniTurbo_aACP_	GATATTAACTAAACATTTCGAAAAAGTAAAATCAcctaggggaagtggaggaatcccgct
P9	miniTurbo_aACP_	GATAAGCGTAATCTGGAACATCGTATGGGTAGGCATAATCTGGAACATCGTAAGGATACG
P10	PKII_TEOE_F	aaacacgatttttctcgagATGAATTTAATTCATATTTGTTTATTTATC
P11	PKII_Myc3_R	TCTGAGATGAGTTTTTGTTCCCTAGGATTTGTTAGACATGGTTGACATAA
P12	Myc3_PKII_F	TTATGTCAACCATGTCTAACAAATCCTAGGGAACAAAAACTCATCTCAGAAGAGGATCTG
P13	Myc3_PKII_R	gtacataaatatattatataactcgacgCGGCCGC
P14	aACP_KD_gRNA_	TAAGTATATAATATTAGACCTCGTTGAATTAATTAGTTTTAGAGCTAGAA
P15	aACP_KD_gRNA_	TTCTAGCTCTAAAACTAATTAATTCAACGAGGTCTAATATTATATACTTA
P16	aACP_5'UTR_F	CCATAACGATAGGGGAGAAATATTATATATAGC
P17	aACP_3'UTR_R	ATAAAAATACCGTTAACTTAAGATATATGTTAACCCAAATTAGCTAGCCAATTCATTT
P18	pMG75_apt_R	CCCAGGCCTCTAGTTTAC
P19	aACPKO_5'HF_F	gccacgaGCGGCCtgaagtatatatccacgtccataacag
P20	aACPKO_5'HF_R	aagcgcaGCGGCCccatctttttgtgtatttttaaaagct
P21	aACPKO_3'HF_F	cgacagacGCCGGggtacaaaaaaataaaatgatacacaa
P22	aACPKO_3'HF_R	ggccaccaGCCGGgggatacattttaacaaagaagaaata
P23	ACPSKO_5'HF_F	gccacgaGCGGCCccaggagttcacacacataatagattg
P24	ACPSKO_5'HF_R	aagcgcaGCGGCCattcggcatttgtttttgtattcaatg
P25	ACPSKO_3'HF_F	cgacagacGCCGGgaggggtcaataaattgttttccaata
P26	ACPSKO_3'HF_R	ggccaccaGCCGGaccctctaccaatagatttgacgatag
P27	FabDKO_5'HF_F	gccacgaGCGGCCacactcacactcacactcacattatcc
P28	FabDKO_5'HF_R	aagcgcaGCGGCCacgaccctatcttatttgtattctcct
P29	FabDKO_3'HF_F	cgacagacGCCGGgtggaaaaccagaatctatggattatc
P30	FabDKO_3'HF_R	ggccaccaGCCGGggtggtatcatatatatggaattgtcc
P31	aACPKO_gRNA_F	TAAGTATATAATATTaatgaactcaaattttaccaGTTTTAGAGCTAGAA
P32	aACPKO_gRNA_R	TTCTAGCTCTAAAACtggtaaaatttgagttcattAATATTATATACTTA
P33	ACPSKO_gRNA_F	TAAGTATATAATATTtaaaatattgaacccacgagGTTTTAGAGCTAGAA
P34	ACPSKO_gRNA_R	TTCTAGCTCTAAAACctcgtgggttcaatattttaAATATTATATACTTA
P35	FabDKO_gRNA_F	TAAGTATATAATATTgatagaaaatttggtttatgGTTTTAGAGCTAGAA
P36	FabDKO_gRNA_R	TTCTAGCTCTAAAACcataaaccaaattttctatcAATATTATATACTTA
P37	ACP.5F	cctatgtttgtgtactttttttttcccc
P38	ACP.5WTR	ggttgcttcttttctttttaatatttgtaag
P39	ACP.3WTF	cccatctagctctttaaaaagtacttttg
P40	ACP.3R	catacataaatatatgaacatttttataaagaggtaac
P41	ACPS.5F	gtaatatttgtggatatatatttctttacataccttgc
P42	ACPS.5WTR	gtaatatcatttctcttacaaaaagaagataaaatattc
P43	ACPS.3WTF	cagaacaagttgtaatgtttatgatattagtgttg

P44	ACPS.3R	cctaattgtctagctattttttttgctttg
P45	FabD.5F	gagaagatatattaaaagagaaatttttttttttttttcg
P46	FabD.5WTR	ttccttttatatctttatttacactaacattatgtctaag
P47	FabD.3WTF	acaaaatgaatgatgatatttttattgttagttatatgac
P48	FabD.3R	ctttaaaatataaaatatactttttttgtcaatttgtaag
P49	pRS.R	TACAAAATGCTTAAGCGCAGCGGCC
P50	pRS.F	CATATTTATTAAATCTAGAATTCGACAGACGCCG
P51	LDH.F	GGAGATGTAGTTTTGTTCGATATTG
P52	LDH.R	CTTGTAAAGGGATACCACCTACAG
P53	SufB.F	CATGTAGCTATAGTAGAAATAATAGTAAAAGATTATGG
P54	SufB.R	GACTCTGAAATACTTAAACCACGTTGC
P55	Cox1.F	CTTCATCTTTAAGAATAATTGCACAAGAAAATGTAAATC
P56	Cox1.R	GGAAGCTTAGTATGGGTACATCATATGTAC
