# Supplementary material for: Acyl carrier protein is essential for apicoplast biogenesis in malaria parasites independent of fatty acid synthesis
Source: eLife. 2026 Sep 14;15:RP111494. doi: 10.7554/eLife.111494 (PMC13574338; doi:10.7554/eLife.111494)
Supplement: Supplementary file 3 [file elife-111494-supp2.pdf]

A

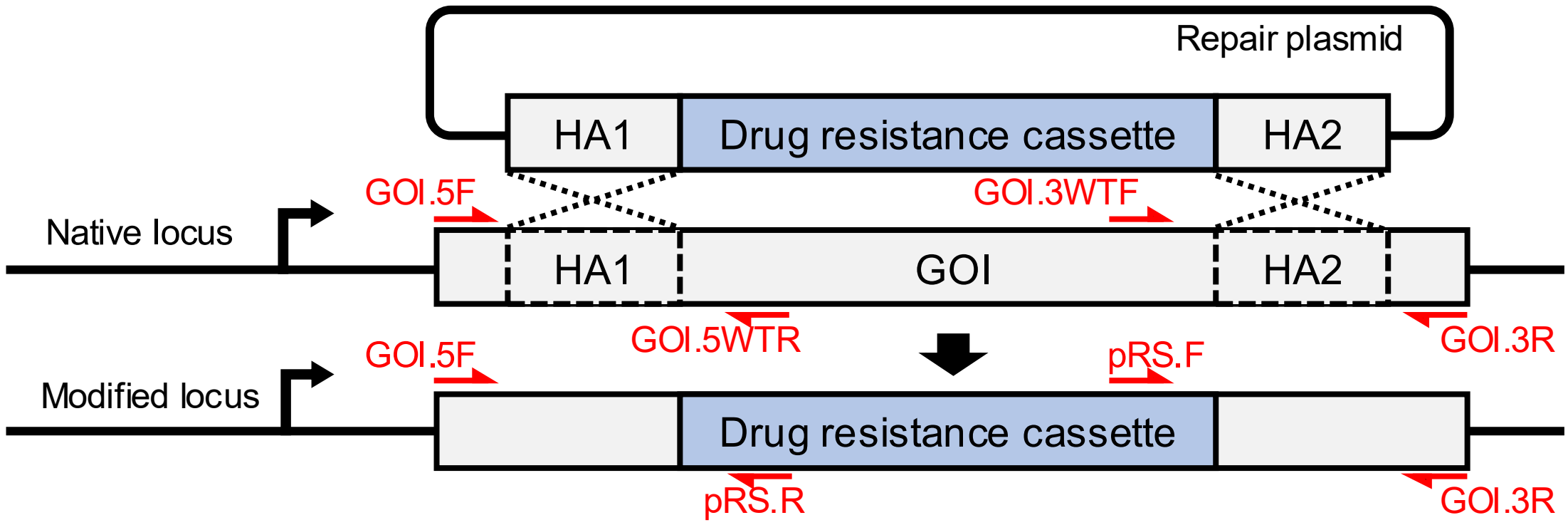

B

PCR reaction	Primer combination		Parasite line
	Forward	Reverse	
Δ5’	GOI.5F	pRS.R	Gene knockout lines
Δ3’	pRS.F	GOI.3R	
5’	GOI.5F	GOI.5WTR	
3’	GOI.3WTF	GOI.3R	
Δ5’	GOI.5F	pRS.R	PfMev (parental)
Δ3’	pRS.F	GOI.3R	
5’	GOI.5F	GOI.5WTR	
3’	GOI.3WTF	GOI.3R	

C

	Anticipated amplicon sizes (bp)			
Gene of interest	Δ5’	Δ3’	5’	3’
acp	526	526	539	596
acps	547	584	560	654
fabD	488	547	501	617

Genomic PCR analysis to confirm targeted gene disruption. (A) Schematic depiction of targeted gene and primer-annealing sites. (B) Primer combinations to test 5’ or 3’ integration or confirm parental locus. (C) Expected PCR amplicon sizes for indicated primer pairs.
