# Supplementary material for: Acyl carrier protein is essential for apicoplast biogenesis in malaria parasites independent of fatty acid synthesis
Source: eLife. 2026 Sep 14;15:RP111494. doi: 10.7554/eLife.111494 (PMC13574338; doi:10.7554/eLife.111494)

Uncropped WB for detection of ACP-KD in Dd2 upon aTc washout

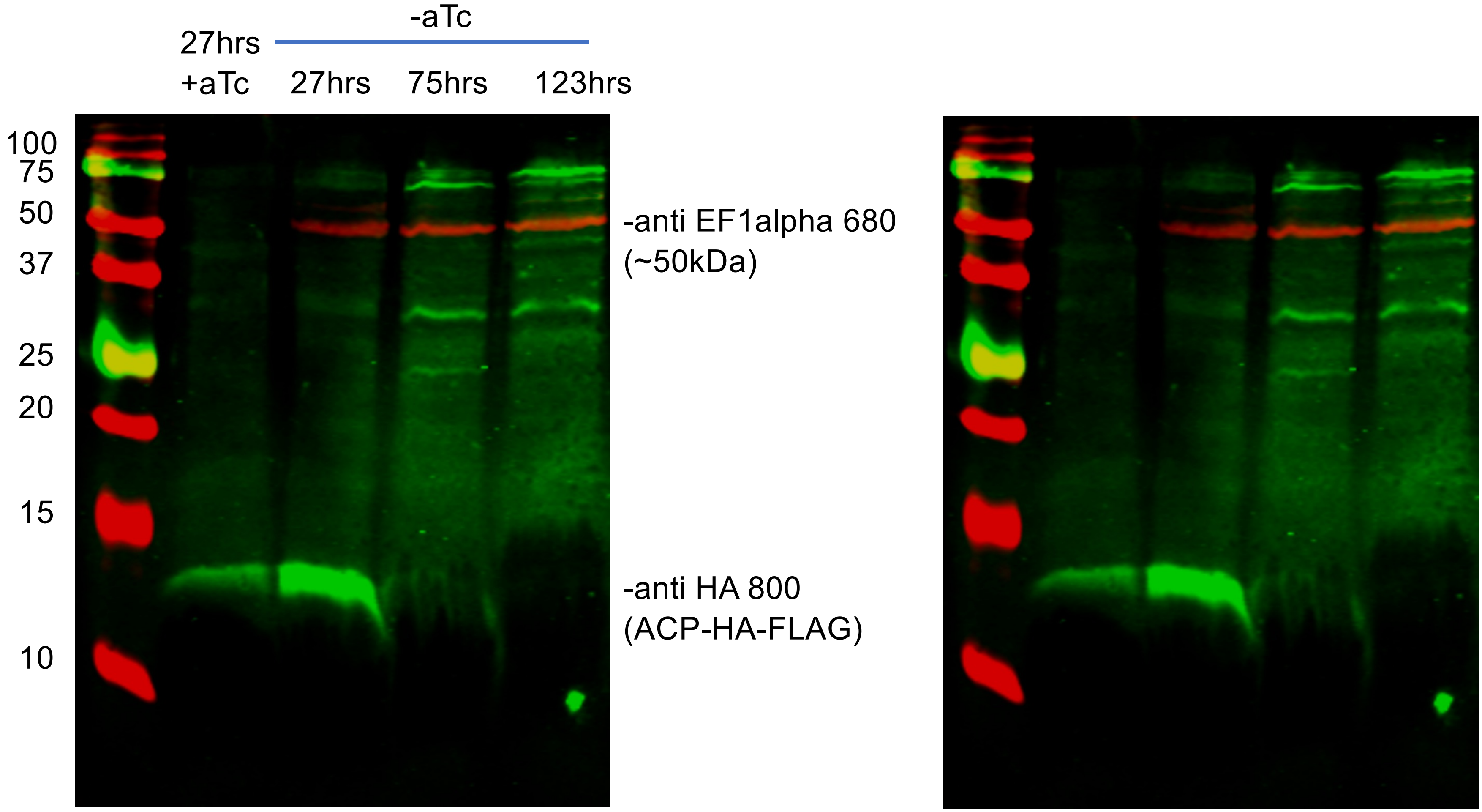

Supplement: Supplementary file 4 [file elife-111494-fig2-data1.zip › Figure 2_Source Data_1/Figure 2C_Source Data_1.pdf]
