# Supplementary material for: Acyl carrier protein is essential for apicoplast biogenesis in malaria parasites independent of fatty acid synthesis
Source: eLife. 2026 Sep 14;15:RP111494. doi: 10.7554/eLife.111494 (PMC13574338; doi:10.7554/eLife.111494)
Supplement: Supplementary file 4 [file elife-111494-fig2-data1.zip › Figure 2_Source Data_1/Figure 2B_Source Data_1.pdf]

Uncropped PCR gels for organellar genomes in ACP-KO and Wt Mev Bypass NF54

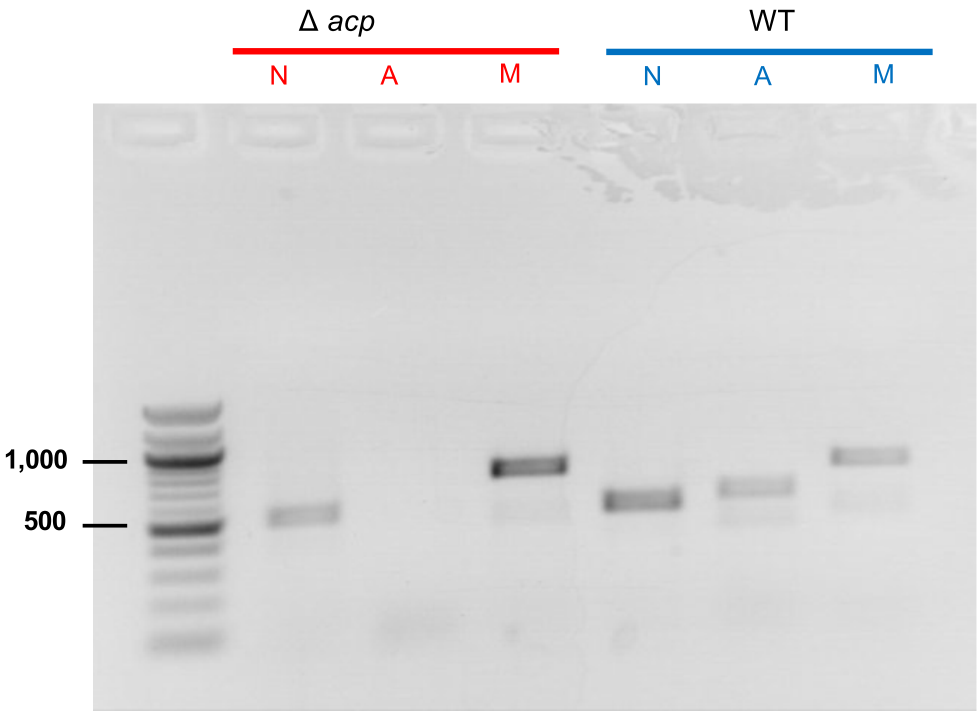

Unlabeled

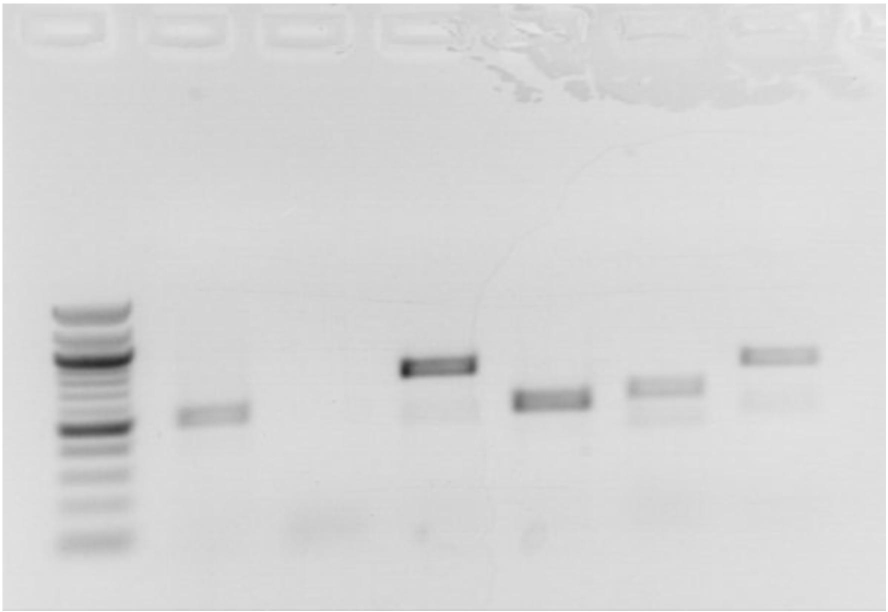

N = nuclear gene
A = apicoplast gene
M = mitochondrial gene
