# Supplementary material for: Acyl carrier protein is essential for apicoplast biogenesis in malaria parasites independent of fatty acid synthesis
Source: eLife. 2026 Sep 14;15:RP111494. doi: 10.7554/eLife.111494 (PMC13574338; doi:10.7554/eLife.111494)

Figure 2 – figure supplement 5- source data 1
Uncropped gel image for western blot validation of custom anti-ACP antibody.

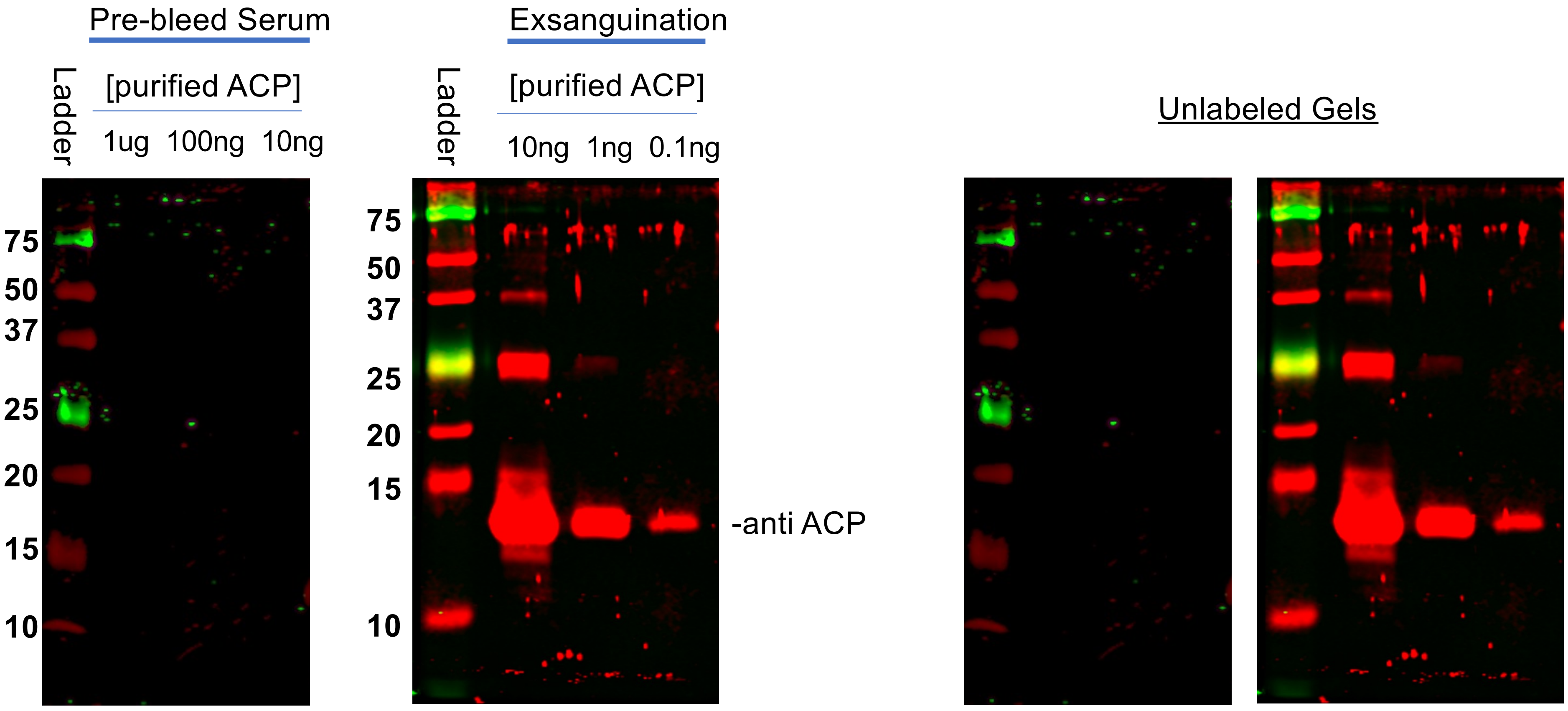

Supplement: Supplementary file 8 [file elife-111494-fig2-figsupp5-data1.zip › Figure 2_FS5_SD1/Figure 2_FS5_Source Data 1.pdf]
