# Supplementary material for: Acyl carrier protein is essential for apicoplast biogenesis in malaria parasites independent of fatty acid synthesis
Source: eLife. 2026 Sep 14;15:RP111494. doi: 10.7554/eLife.111494 (PMC13574338; doi:10.7554/eLife.111494)
Supplement: Supplementary file 10 [file elife-111494-fig3-data1.zip › Figure 3_Source Data 1/Figure 3B_Source Data 1.pdf]

Figure 3 – Source Data 1

Uncropped PCR gels for organellar genomes in ACPS-KO and Wt PfMev NF54

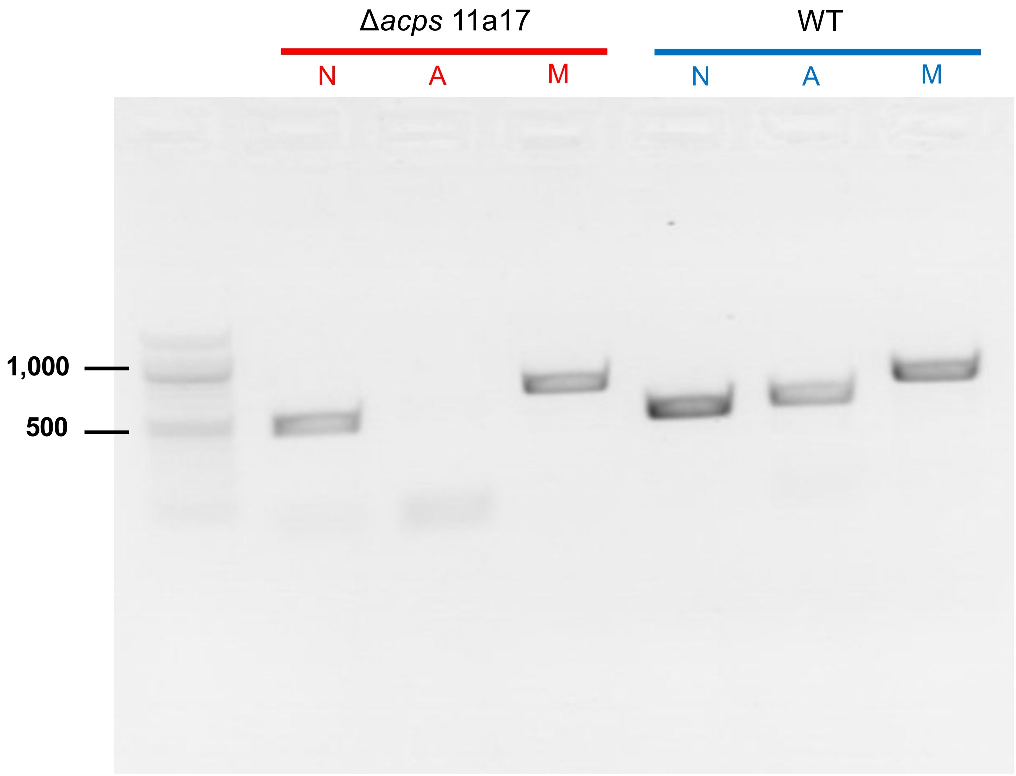

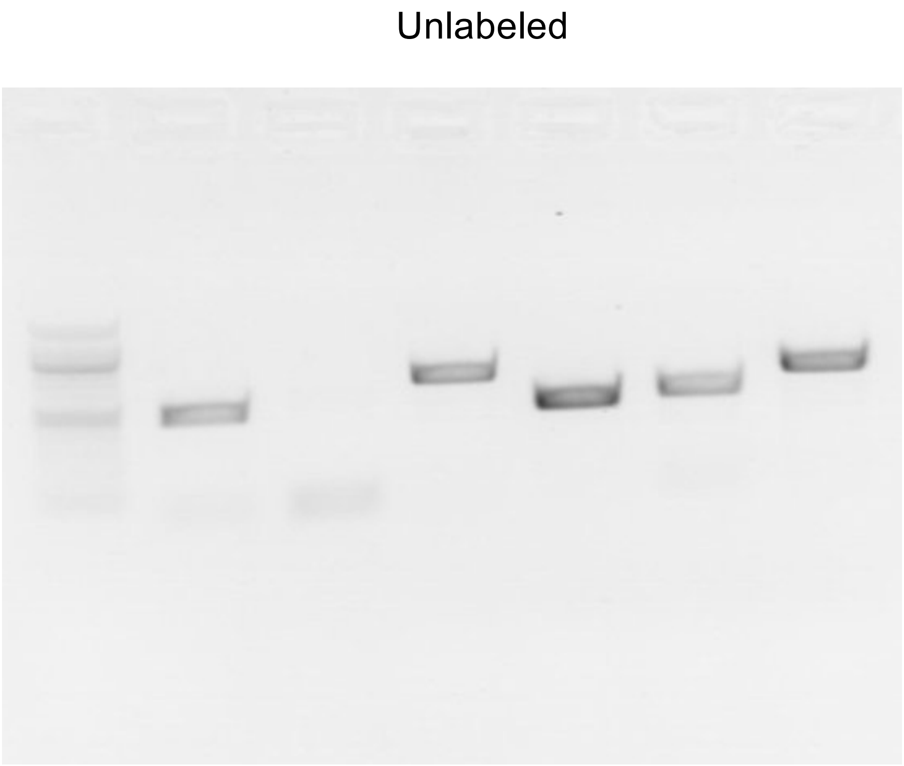

N = nuclear gene
A = apicoplast gene
M = mitochondrial gene
