# Supplementary material for: Acyl carrier protein is essential for apicoplast biogenesis in malaria parasites independent of fatty acid synthesis
Source: eLife. 2026 Sep 14;15:RP111494. doi: 10.7554/eLife.111494 (PMC13574338; doi:10.7554/eLife.111494)

Figure 3 – Figure Supplement 1B- source data 1
Uncropped PCR gels for deletion of FabD in PfMev NF54

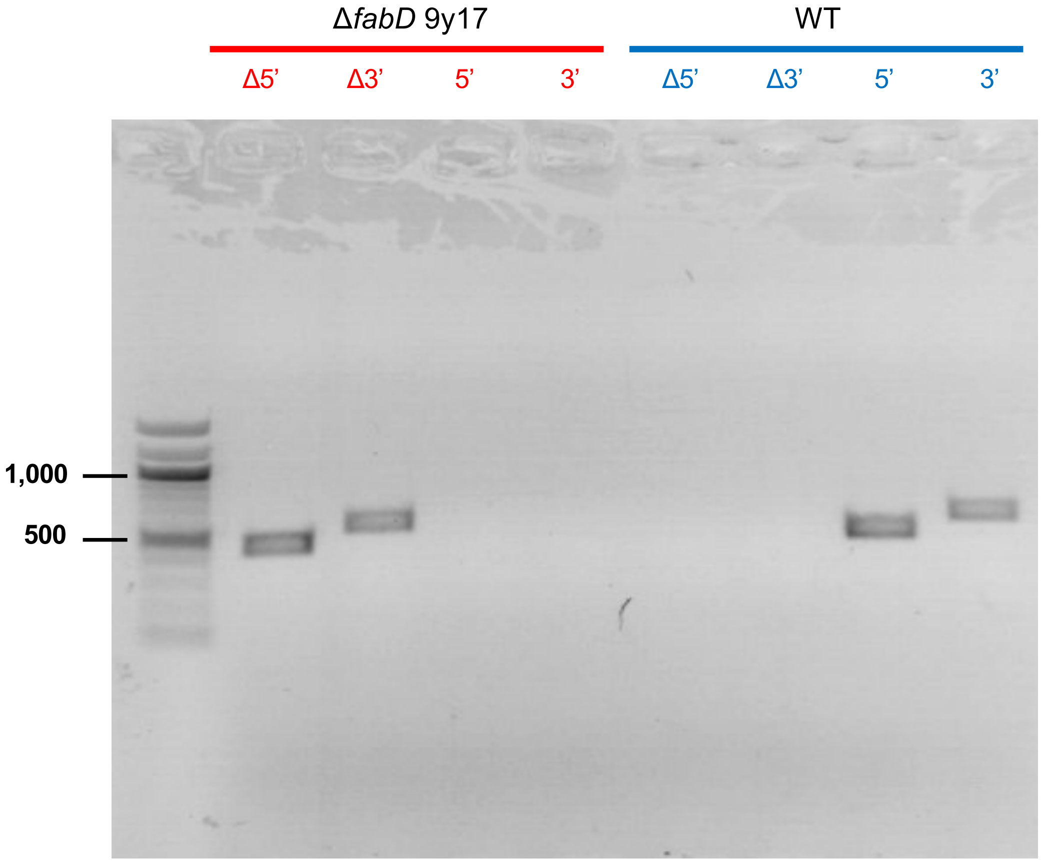

Unlabeled

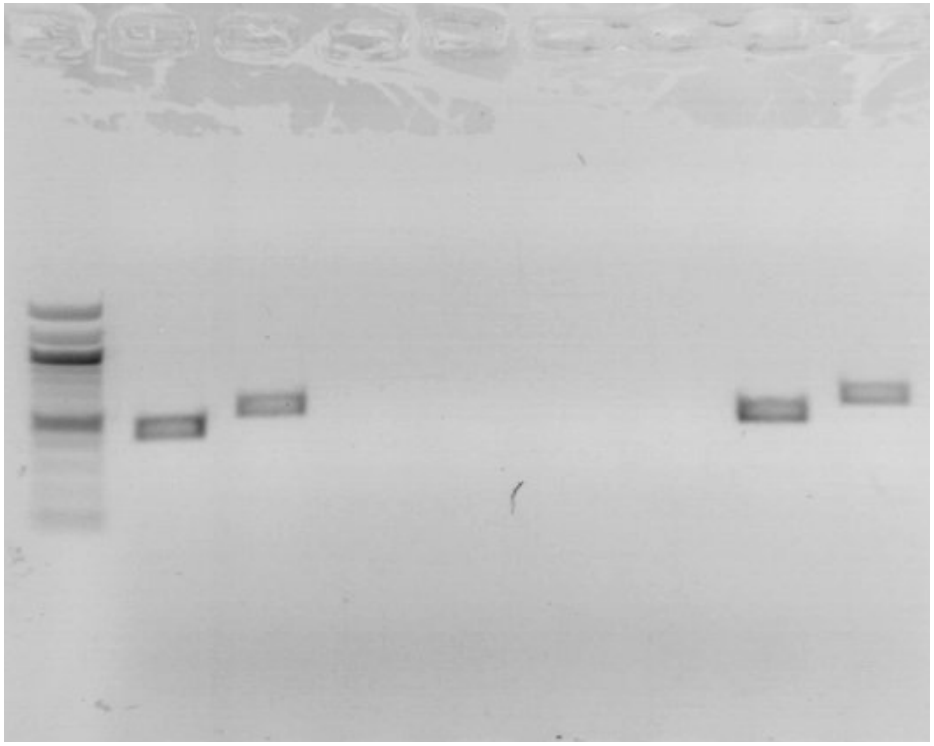

Supplement: Supplementary file 12 [file elife-111494-fig3-figsupp1-data1.zip › Figure 3_FS1_SD1/Figure 3_FS1B_Source Data 1.pdf]
