# Supplementary material for: Acyl carrier protein is essential for apicoplast biogenesis in malaria parasites independent of fatty acid synthesis
Source: eLife. 2026 Sep 14;15:RP111494. doi: 10.7554/eLife.111494 (PMC13574338; doi:10.7554/eLife.111494)
Supplement: Supplementary file 14 [file elife-111494-fig4-data1.zip › Figure 4_Source Data 1/Figure 4D_Source Data 1_WT aACP_rep 1_aACP.pdf]

Figure 4 – source data 1- rep 1- aACP

Uncropped Western Blots of immunoprecipitation of aACP and PKII in *E. coli*

Replicate 1 – Probed for aACP

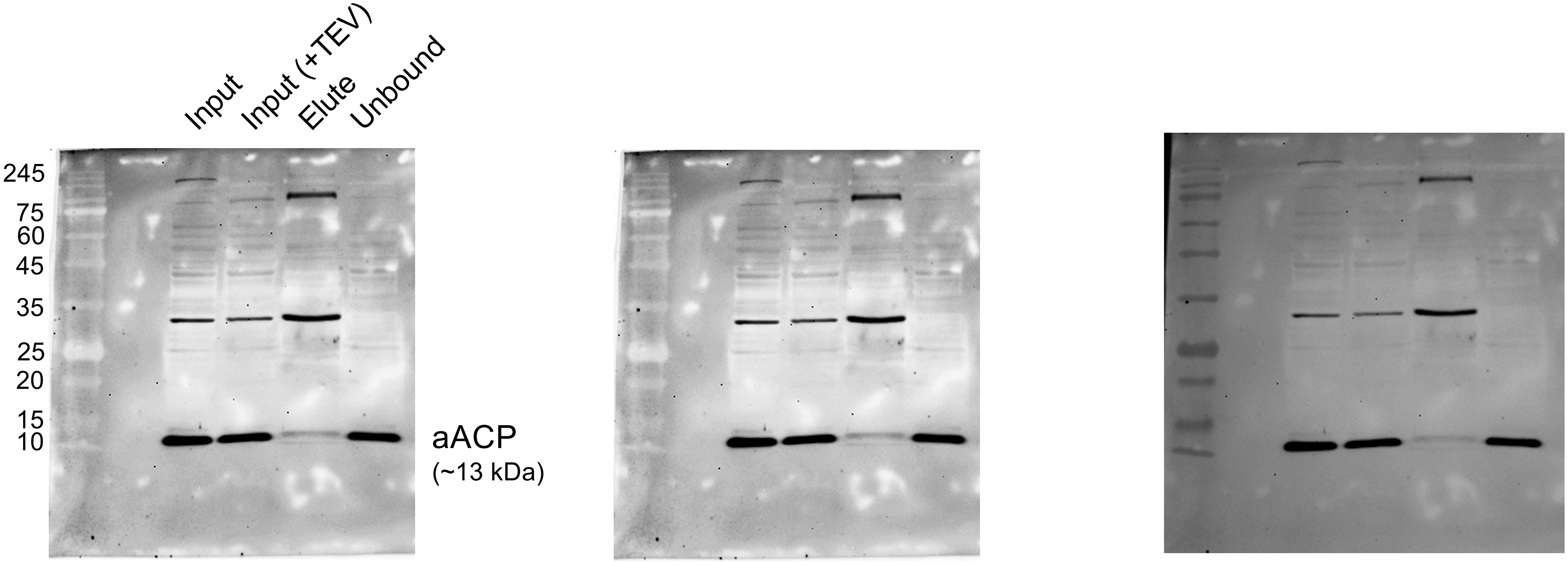

Chemi Labeled

Chemi Unlabeled

Membrane Overlay Unlabeled

Ni-NTA pulldown (PKII 6xHis)
TEV protease-treated
