# Supplementary material for: Acyl carrier protein is essential for apicoplast biogenesis in malaria parasites independent of fatty acid synthesis
Source: eLife. 2026 Sep 14;15:RP111494. doi: 10.7554/eLife.111494 (PMC13574338; doi:10.7554/eLife.111494)

Figure 4 – figure supplement 1- source data 1

Uncropped blot for western blot analysis in Figure 4- figure supplement 1

Anti-Streptavidin-800 probing after anti-Streptavidin IP

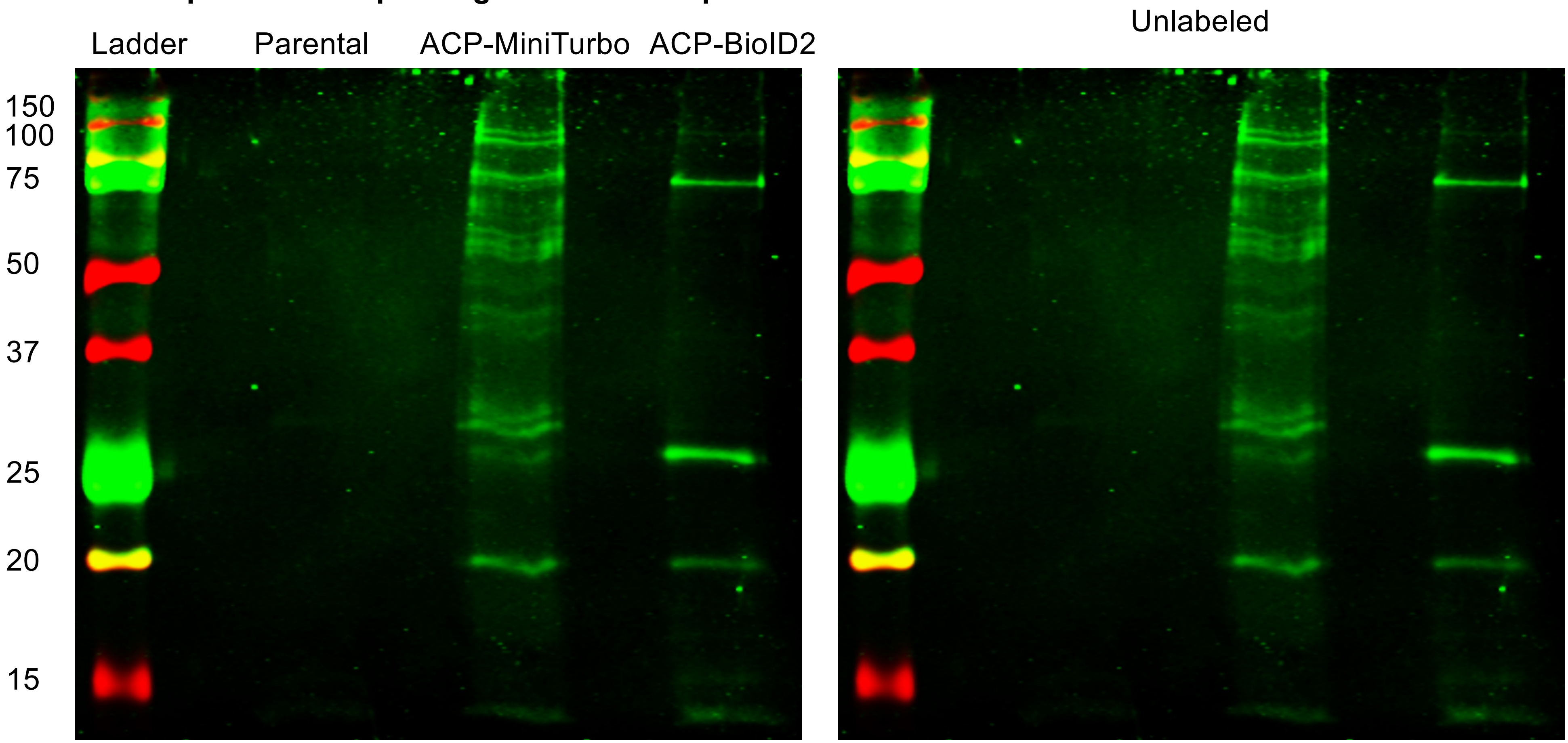

Supplement: Supplementary file 16 [file elife-111494-fig4-figsupp1-data1.zip › Figure 4_FS1_SD1/Figure 4_FS1_Source Data 1.pdf]
