# Supplementary material for: Acyl carrier protein is essential for apicoplast biogenesis in malaria parasites independent of fatty acid synthesis
Source: eLife. 2026 Sep 14;15:RP111494. doi: 10.7554/eLife.111494 (PMC13574338; doi:10.7554/eLife.111494)
Supplement: Supplementary file 18 [file elife-111494-fig4-figsupp3-data1.zip › Figure 4_FS3_SD1/Figure 4_FS3_Source Data 1_WT aACP_rep 2_aACP.pdf]

Figure 4 – figure supplement 3- source data 1- WT aACP- rep 2- aACP
Uncropped Western Blots of immunoprecipitation of aACP and PKII in *E. coli*
Replicate 2 – Probed for aACP

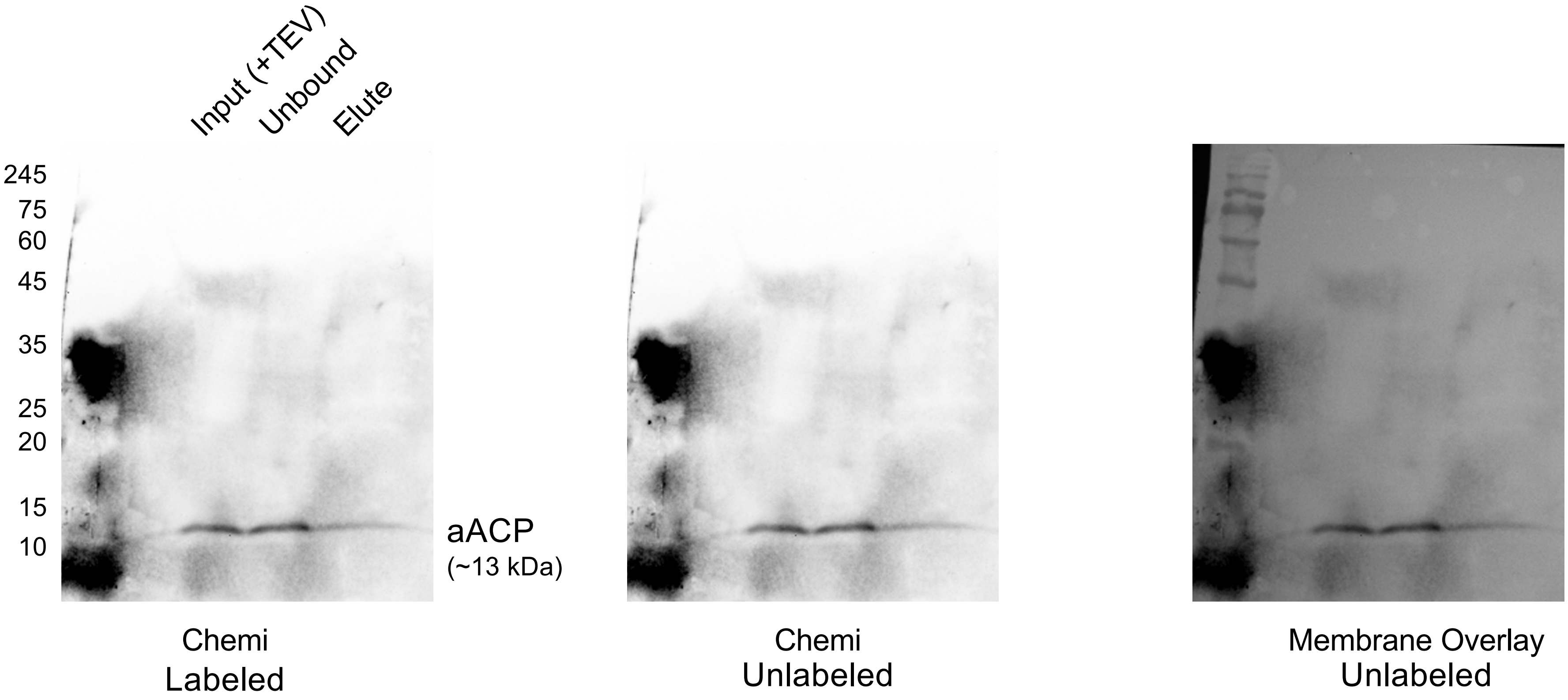

Ni-NTA pulldown (PKII 6xHis)
TEV protease-treated
