# Supplementary material for: Acyl carrier protein is essential for apicoplast biogenesis in malaria parasites independent of fatty acid synthesis
Source: eLife. 2026 Sep 14;15:RP111494. doi: 10.7554/eLife.111494 (PMC13574338; doi:10.7554/eLife.111494)
Supplement: Supplementary file 20 [file elife-111494-fig5-data1.zip › Figure 5_Source Data 1/Figure 5_rep 1_PKII.pdf]

Figure 5 – source data 1_rep 1_PKII
Uncropped Western Blots of PKII levels upon ACP-KD
Replicate 1 – Probed for PKII

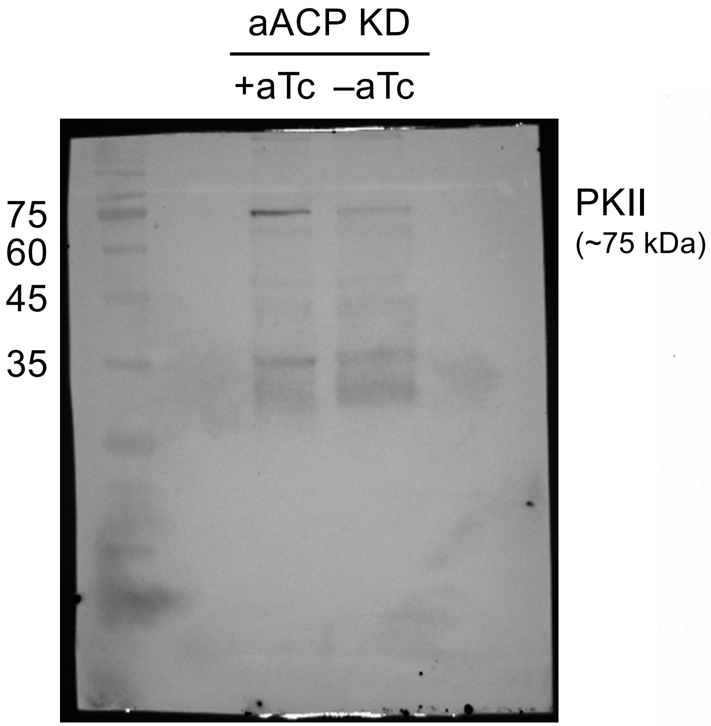

Membrane Overlay

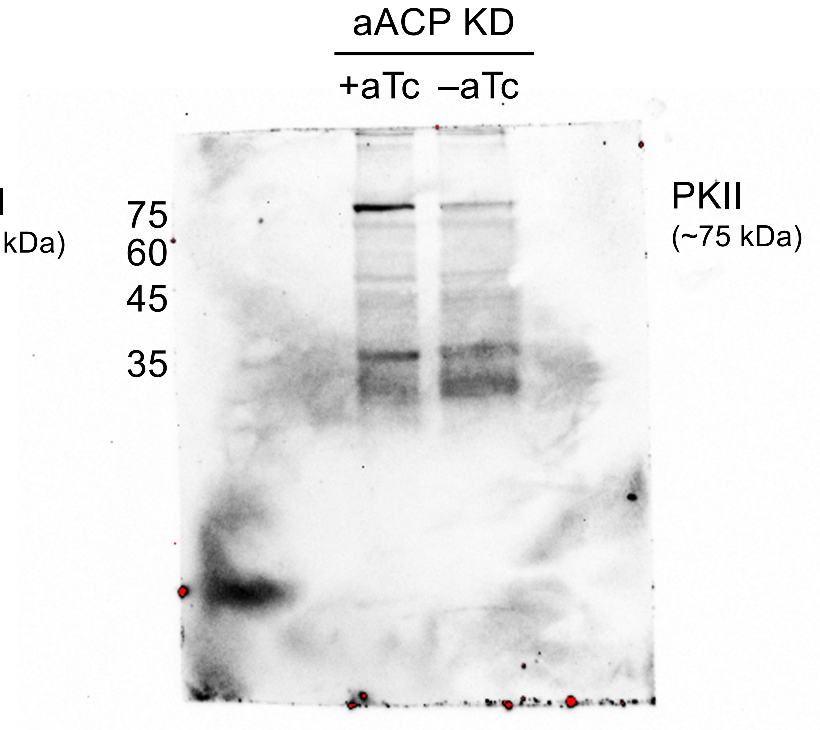

Chemi

Labeled
