# Supplementary material for: Acyl carrier protein is essential for apicoplast biogenesis in malaria parasites independent of fatty acid synthesis
Source: eLife. 2026 Sep 14;15:RP111494. doi: 10.7554/eLife.111494 (PMC13574338; doi:10.7554/eLife.111494)

Figure 5 – source data 1

Uncropped Western Blots of PKII levels upon ACP-KD

Replicate 2 – Probed for PKII

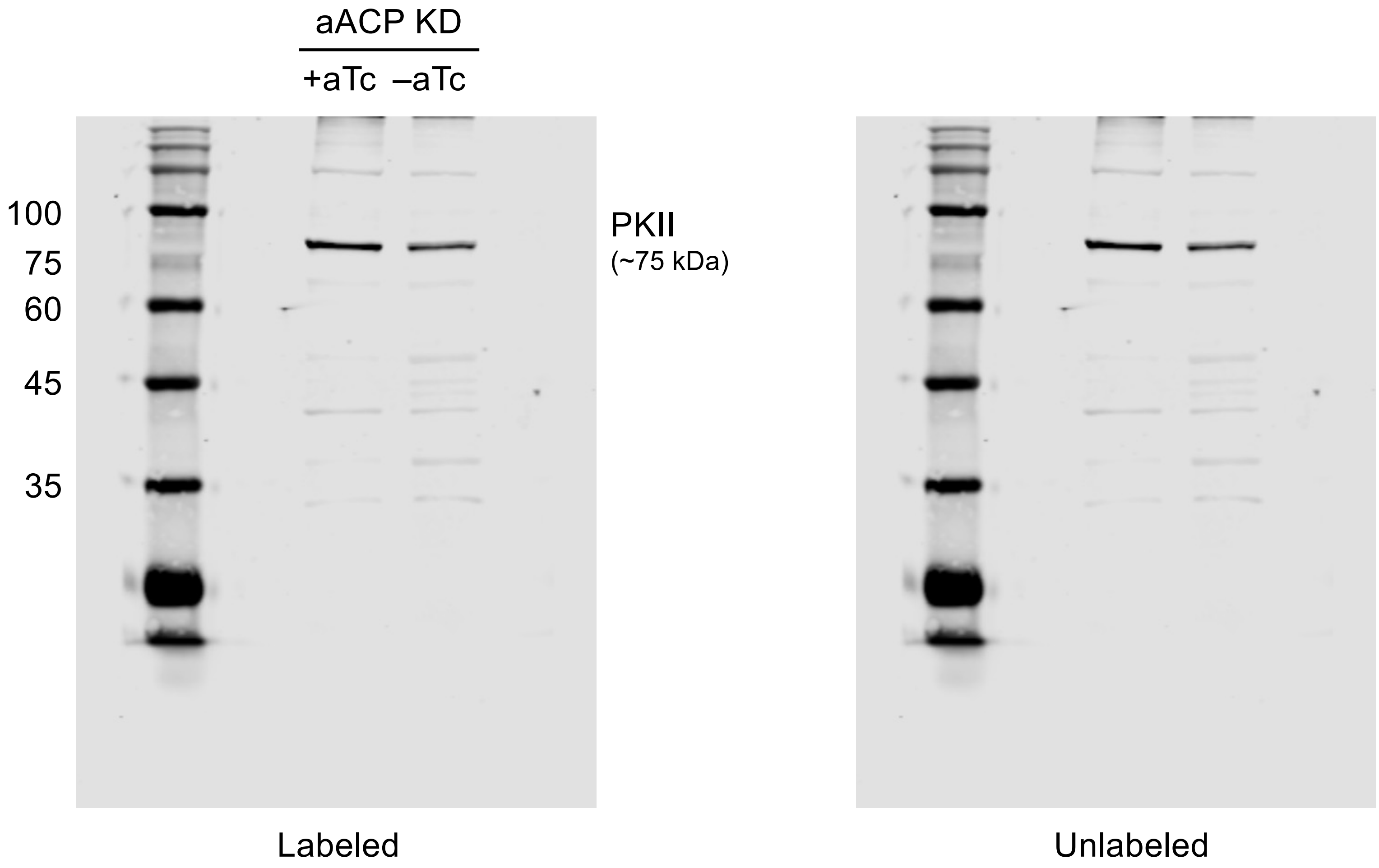

Supplement: Supplementary file 22 [file elife-111494-fig5-figsupp1-data1.zip › Figure 5_FS1_SD1/Figure 5_FS1_SD1_rep 2_PKII.pdf]
