# Supplementary material for: Acyl carrier protein is essential for apicoplast biogenesis in malaria parasites independent of fatty acid synthesis
Source: eLife. 2026 Sep 14;15:RP111494. doi: 10.7554/eLife.111494 (PMC13574338; doi:10.7554/eLife.111494)
Supplement: Peer review history

# Editor report: eLife Assessment

**DOI:** 10.7554/eLife.111494.3.sa0

**Authors:** Dominique Soldati-Favre

**Affiliations:** University of Geneva Switzerland

**Keywords:** Compelling, Important

This **important** study identifies a non-canonical essential role for acyl carrier protein in maintaining apicoplast metabolism and blood-stage survival in *Plasmodium falciparum*. The main conclusions are **compelling**, and supported by strong genetic and biochemical evidence, while function of fatty acid synthesis pathways in low-lipid conditions will require future studies to fully resolve. The work provides novel mechanistic insight into ACP-mediated stabilization of pyruvate kinase II and will be of broad interest to the malaria and apicoplast biology communities.

# Referee report: Reviewer #1 (Public review)

**DOI:** 10.7554/eLife.111494.3.sa1

[Editors' note: this version has been assessed by the Reviewing Editor without further input from the original reviewers. The authors have addressed the comments raised in the previous round of review.]

This study provides evidence that the apicoplast-locaized isoform of acyl-carrier protein (ACP) has acquired important non-enzymatic functions in the malaria parasite. Previous studies have shown that the apicoplast-located FASII-dependent pathway of fatty acid synthesis is not essential in Plasmodium blood stages. In contrast, genome-wide knockout studies suggested that ACP, a key protein in this pathway, is essential in these stages, indicating that it may have additional non-canonical functions. In this study, the authors confirm that ACP is essential in Pf blood stages (using both apicoplast IPP rescue and conditional knockdown); show that this essential function requires modification with 4-phosphopantetheine and use proximity biotinylation and complementary immunoprecipitation pull-down approaches to provide compelling evidence that ACP binds to and stabilizes the apicoplast-located isoform of pyruvate kinase II. Notably, these interactions appear to differ from those associated with the binding of mitochondrial isoforms of ACP to proteins involved in Fe-S biosynthesis. Loss of ACP was shown to lead to a decrease in PKII levels and apicoplast DNA/RNA synthesis, consistent with loss of NTP synthesis in this organelle. The data are clear and very well described, and the findings represent a significant advance in our understanding of metabolic regulatory mechanisms in apicomplexan apicoplast studies.

Strengths:

The study uses a variety of complementary genetic approaches to demonstrate the essentiality of ACP and the enzyme involved in its activation with 4-PP in Pf blood stages, demonstrating that the ascribed non-enzymatic function is mediated by holo-ACP. Similarly, a number of complementary biochemical approaches, including proximity biotinylation, immunoprecipitation, and co-expression of PfACP and PK-II in a heterologous bacterial expression system, are used to confirm the physiological significance of the PfACP and PK-II interaction. The study also reports additional findings, such as the independence of P. faciparum blood stages on exogenous (media) fatty acids, indicating that intracellular stages can salvage all of their requirements from the red blood cell.

Weaknesses:

Overall, this is a very strong study. While questions remain around the function of other apicoplast ACP-interacting proteins detected in this study, I don't have any suggestions for significant improvements.

# Referee report: Reviewer #2 (Public review)

**DOI:** 10.7554/eLife.111494.3.sa2

This study focuses on revealing the essential divergent function of the Acyl Carrier protein (ACP) in the deadliest human malaria parasite, *Plasmodium falciparum*. More precisely, using inducible KO, cellular and biochemical approaches, the authors determined that instead of a canonical role for ACP allowing the de novo synthesis of fatty acids in the apicoplast (essential relict plastid) of the parasite, the enzyme couples with pyruvate kinase II to generate nucleoside triphosphate to maintain parasite survival during blood stages. The study is novel, well-designed, providing interesting new data on Plasmodium and apicomplexa biology. The results convincingly support the major claim of the study. However, it is currently incomplete to support some claims on the essentiality of some apicoplast pathways.

In this study, Geher et al. focused on deciphering the role of the Acyl Carrier Protein (ACP) present in the relict non-photosynthetic plastid, i.e. the apicoplast of the most lethal human malaria parasite, *Plasmodium falciparum*. More particularly, they determined an essential function of ACP independent of its usual/typical function as the central protein for the normal function of the apicoplast Type II fatty acid synthesis (FASII) pathway. Rather, the protein seems to associate with the apicoplast Pyruvate Kinase II, together generating an essential nucleoside triphosphate (NTPs) source to fuel the apicoplast and parasite survival instead.

By generating a TetR-DOZY-based inducible KD line for ACP, they confirmed that the protein is indeed essential to maintain apicoplast integrity and parasite survival during asexual blood stages, as previously predicted and experimentally shown. They showed that ACP requires a biochemical modification, typically activating the protein for its function in the FASII pathway, i.e. binding of the 4-PP group by holoACP synthase. Then, they showed that the other enzymes of the FASII pathway are likely dispensable during the blood stage, as they were able to generate a KO line of the first enzyme of the pathway, FabD (which was predicted to be essential in *P. falciparum*). Based on a cell culture approach in a controlled culture medium, they further claimed that, unlike current evidence-based hypotheses, the FASII pathway (and thus a potentially FASII-linked ACP) has no role/activity during blood stages. Using a proximity biotinylation approach, they determined that ACP associates with the apicoplast pyruvate Kinase II (PKII), previously shown to generate NTPs in the apicoplast for energy and DNA/RNA maintenance (Xia et al. 2019), and not to fuel the FASII pathway as its main function in blood stages. Finally, they showed that the disruption of ACP induces the reduction of the presence/content in PKII in the parasite, as well as the drastic reduction of the apicoplast DNA and RNA content. Together, they concluded that the main function of ACP is indeed the NTP formation via its association with PKII, rather than its canonical role for the generation of fatty acids in the apicoplast.

This study is novel and focuses on a topic of particular interest in malaria biology, but also for most of the apicomplexa-related diseases, and beyond for plastid bearing orgnaisms and this unusual role for ACP. The study is well thought out with proper biochemical approaches that convincingly point to this association of ACP with PKII for NTP synthesis as a major function during *P. falciparum* blood stages.

# Author comment: Author response

**DOI:** 10.7554/eLife.111494.3.sa3

**Authors:** Sage WR Geher, Seyi Falekun, Jessica N Pita-Aquino, Russell P Swift, Megan Okada, Yasaman Jami-Alahmadi, James A Wohlschlegel, Sean T Prigge, Paul A Sigala

**Affiliations:** University of Utah Salt Lake City United States; University of Utah Salt Lake City United States; University of Utah Salt Lake City United States; Johns Hopkins University Baltimore United States; University of Utah Salt Lake City United States; University of California, Los Angeles Los Angeles United States; University of California, Los Angeles Los Angeles United States; Johns Hopkins University Baltimore United States; University of Utah Salt Lake City United States

The following is the authors’ response to the original reviews.

**eLife Assessment**

This important study identifies a non-canonical essential role for acyl carrier protein in maintaining apicoplast metabolism and blood-stage survival in *Plasmodium falciparum*. The main conclusions are largely supported by strong genetic and biochemical evidence, although some claims regarding the dispensability of fatty acid synthesis pathways remain incomplete. The work provides novel mechanistic insight into ACP-mediated stabilization of pyruvate kinase II and will be of broad interest to the malaria and apicoplast biology communities.

We note that the major and most important conclusion of our manuscript is that apicoplast ACP has an essential stabilizing interaction with pyruvate kinase II that is required for organelle function and biogenesis. This conclusion is entirely independent of our growth experiments with ∆ACP and ∆FabD parasites in low-lipid conditions, which in principle could be removed from the manuscript without weakening the major conclusions. Nonetheless, we feel that these findings in low-lipid conditions have merit, especially since they contrast with a prior study in the literature regarding *P. falciparum* growth in low-lipid conditions. We hope that these contrasting results and the questions they raise will stimulate future studies to fully test and understand FASII function under different conditions, including low-lipid conditions.

We kindly ask that the editorial assessment be revised to focus on the major conclusions. Alternatively, we would respectfully suggest revising the second sentence to something akin to “The main conclusions are largely supported by strong genetic and biochemical evidence, while function of fatty acid synthesis pathways in low-lipid conditions will require future studies to fully resolve.”

**Public Reviews:**

**Reviewer #1 (Public review):**

This study provides evidence that the apicoplast-locaized isoform of acyl-carrier protein (ACP) has acquired important non-enzymatic functions in the malaria parasite. Previous studies have shown that the apicoplast-located FASII-dependent pathway of fatty acid synthesis is not essential in Plasmodium blood stages. In contrast, genome-wide knockout studies suggested that ACP, a key protein in this pathway, is essential in these stages, indicating that it may have additional non-canonical functions. In this study, the authors confirm that ACP is essential in Pf blood stages (using both apicoplast IPP rescue and conditional knockdown); show that this essential function requires modification with 4-phosphopantetheine and use proximity biotinylation and complementary immunoprecipitation pull-down approaches to provide compelling evidence that ACP binds to and stabilizes the apicoplast-located isoform of pyruvate kinase II. Notably, these interactions appear to differ from those associated with the binding of mitochondrial isoforms of ACP to proteins involved in Fe-S biosynthesis. Loss of ACP was shown to lead to a decrease in PKII levels and apicoplast DNA/RNA synthesis, consistent with loss of NTP synthesis in this organelle. The data are clear and very well described, and the findings represent a significant advance in our understanding of metabolic regulatory mechanisms in apicomplexan apicoplast studies.

Strengths:

The study uses a variety of complementary genetic approaches to demonstrate the essentiality of ACP and the enzyme involved in its activation with 4-PP in Pf blood stages, demonstrating that the ascribed non-enzymatic function is mediated by holo-ACP. Similarly, a number of complementary biochemical approaches, including proximity biotinylation, immunoprecipitation, and co-expression of PfACP and PK-II in a heterologous bacterial expression system, are used to confirm the physiological significance of the PfACP and PK-II interaction. The study also reports additional findings, such as the independence of P. faciparum blood stages on exogenous (media) fatty acids, indicating that intracellular stages can salvage all of their requirements from the red blood cell.

Weaknesses:

Overall, this is a very strong study. While questions remain around the function of other apicoplast ACP-interacting proteins detected in this study, I don't have any suggestions for significant improvements.

We thank the reviewer for these positive comments.

**Reviewer #2 (Public review):**

This study focuses on revealing the essential divergent function of the Acyl Carrier protein (ACP) in the deadliest human malaria parasite, *Plasmodium falciparum*. More precisely, using inducible KO, cellular and biochemical approaches, the authors determined that instead of a canonical role for ACP allowing the de novo synthesis of fatty acids in the apicoplast (essential relict plastid) of the parasite, the enzyme couples with pyruvate kinase II to generate nucleoside triphosphate to maintain parasite survival during blood stages. The study is novel, well-designed, providing interesting new data on Plasmodium and apicomplexa biology. The results convincingly support the major claim of the study. However, it is currently incomplete to support some claims on the essentiality of some apicoplast pathways.

In this study, Geher et al. focused on deciphering the role of the Acyl Carrier Protein (ACP) present in the relict non-photosynthetic plastid, i.e. the apicoplast of the most lethal human malaria parasite, *Plasmodium falciparum*. More particularly, they determined an essential function of ACP independent of its usual/typical function as the central protein for the normal function of the apicoplast Type II fatty acid synthesis (FASII) pathway. Rather, the protein seems to associate with the apicoplast Pyruvate Kinase II, together generating an essential nucleoside triphosphate (NTPs) source to fuel the apicoplast and parasite survival instead.

By generating a TetR-DOZY-based inducible KD line for ACP, they confirmed that the protein is indeed essential to maintain apicoplast integrity and parasite survival during asexual blood stages, as previously predicted and experimentally shown. They showed that ACP requires a biochemical modification, typically activating the protein for its function in the FASII pathway, i.e. binding of the 4-PP group by holoACP synthase. Then, they showed that the other enzymes of the FASII pathway are likely dispensable during the blood stage, as they were able to generate a KO line of the first enzyme of the pathway, FabD (which was predicted to be essential in *P. falciparum*). Based on a cell culture approach in a controlled culture medium, they further claimed that, unlike current evidence-based hypotheses, the FASII pathway (and thus a potentially FASII-linked ACP) has no role/activity during blood stages. Using a proximity biotinylation approach, they determined that ACP associates with the apicoplast pyruvate Kinase II (PKII), previously shown to generate NTPs in the apicoplast for energy and DNA/RNA maintenance (Xia et al. 2019), and not to fuel the FASII pathway as its main function in blood stages. Finally, they showed that the disruption of ACP induces the reduction of the presence/content in PKII in the parasite, as well as the drastic reduction of the apicoplast DNA and RNA content. Together, they concluded that the main function of ACP is indeed the NTP formation via its association with PKII, rather than its canonical role for the generation of fatty acids in the apicoplast.

To clarify, we conclude that the essential function of ACP in blood-stage *P. falciparum* parasites includes a critical stabilizing interaction with pyruvate kinase II. Apicoplast ACP presumably still plays a central biochemical role in FASII pathway function, but that role in FASII is dispensable for blood-stage parasites.

This study is novel and focuses on a topic of particular interest in malaria biology, but also for most of the apicomplexa-related diseases, and beyond for plastid bearing orgnaisms and this unusual role for ACP. The study is well thought out with proper biochemical approaches that convincingly point to this association of ACP with PKII for NTP synthesis as a major function during *P. falciparum* blood stages. However, there are currently some important experimental issues/flaws, missing experiments that induced wrong interpretations and thus do not support some important claims of the study, notably for the role of FASII and the interaction between ACP and PKII.

We note that the major and most important conclusion of our manuscript is that apicoplast ACP has an essential stabilizing interaction with pyruvate kinase II that is required for organelle function and biogenesis. This conclusion is entirely independent of our growth experiments with ∆ACP and ∆FabD parasites in low-lipid conditions, which in principle could be removed from the manuscript without weakening the major conclusions. Nonetheless, we feel that these findings in low-lipid conditions have merit, especially since they contrast with a prior study in the literature regarding *P. falciparum* growth in low-lipid conditions. We hope that these contrasting results and the questions they raise will stimulate future studies to fully test and understand FASII function under different conditions, including low-lipid conditions.

We elaborate on these points and address the reviewer’s critiques below.

Therefore, at this point, the study is only partial and would require major additions and/or important text edits/revisions before being considered for acceptance.

We note that the manuscript has already been accepted for publication in accordance with the current eLife publishing model.

Major points:

From the graph of *P. falciparum* growth, we can see that in the lipid-rich condition, where both FabH KO and ACP KO can survive, the addition of mevalonate was essential for the growth of ACP KO. Along with the other evidence (PKII association, DNA levels...), we therefore agree that PfACP is involved in the mevalonate pathway.

To clarify, our model is that ACP supports IPP synthesis by the apicoplast nonmevalonate/MEP pathway indirectly by stabilizing and thus supporting function by pyruvate kinase II that supplies the pyruvate and NTPs required for IPP synthesis by the MEP pathway.

The authors claim that the FASII pathway is inactive/not essential in the *P. falciparum* blood stage. However, the authors have not shown any evidence on whether ACP is or not involved in the FASII pathway during the asexual blood stage.

To clarify, there is overwhelming data in the prior published literature that we cite (including refs. 13, 14, and 32) to establish that FASII is dispensable for blood-stage *Plasmodium* growth *in vivo* in rodent parasites and *in vitro* culture in human parasites. Prior studies also strongly support a role for apicoplast ACP as the central scaffold for FASII-mediated acyl chain synthesis. However, our and prior studies support the conclusion that essential ACP function in blood-stage parasites is independent of its role in FASII.

As currently designed, the experiments presented cannot conclude on that point for several reasons. Indeed, it was previously shown that (i) the expression of the protein from the FASII pathway are all present in blood stages and are significantly upregulated in patients that are under under "nutrient starvation" (Daily et al. Nature 2007), (ii) that, growing parasites under similar low lipid conditions in vitro induces an activation/upregulation of FASII, which can be measured by stable isotope precursor labelling and lipidomics (Botté et al. 2013).

We are aware of these prior studies and cite and discuss the Botté et al. 2013 reference in our manuscript, which provided isotope-labeling evidence to support FASII activity in low-lipid growth conditions for *P. falciparum*. We note that neither study addresses whether FASII activity is required for growth in low-lipid conditions.

(iii) that growing the PfFabI KO line under deprived lipid conditions leads to parasite death (Amiar et al. 2020), indicating that the FASII pathway can become critical, if not essential, depending on the host nutritionnal content together correlating patients' data and metabolic adaptation for the same reasons in the related parastie *Toxoplasma gondii* (Amiar et al. 2020, Krishnan et al. 2020, Liang et al. 2020, Primo et al. 2021, Charital et al. 2024, Dass et al. 2024, Bitew et al. 2025).

All of the studies cited by the reviewer focus primarily or exclusively on *Toxoplasma gondii* parasites. We agree with the reviewer that these and other studies provide strong evidence that FASII activity contributes to growth of *T. gondii* parasites, including roles for apicoplast ACP that appear to differ from what we have unveiled for *P. falciparum* malaria parasites. We acknowledge and discuss these differences from *T. gondii* in the final section of the Discussion section and think that exploring these differences will be a fascinating area for future study.

The Amiar et al. 2020 paper cited by the reviewer is the only study we are aware of that has directly tested the ability of a ∆FASII parasite (in this case, ∆FabI) to grow in low-lipid conditions. We acknowledge that they observed little to no growth of ∆FabI parasites in these conditions. Our growth assays with ∆ACP and ∆FabD parasites indicated a different outcome in which both WT and ∆FASII parasites grew similarly in low-lipid conditions. Our results thus contrast with the prior study. As noted below, the minimal lipid growth conditions explicitly reported in the methods section of the Amiar et al. paper are identical to those used in our study: fatty acid-free BSA, 30 µM palmitic acid, and 45 µM oleic acid (all sourced from Sigma) with daily media changes. Thus, the basis for these differences is unclear and additional follow-up work will be needed to explore and resolve these differences.

We have revised the final paragraph of the second results section of our manuscript to incorporate this perspective:

“These results contrast with the prior study [49] of ∆FabI parasites and the proposed model that blood-stage *P. falciparum* requires FASII activity for growth in low-lipid conditions and suggest that parasites can rely on scavenging host-derived fatty acids over a wide range of lipid conditions. Future studies involving tandem growth and isotope-labeling experiments of WT and ∆FASII parasites will be required to fully test and understand FASII function and the dependence of *P. falciparum* growth on this pathway in low-lipid conditions.”

Here, the authors are expecting to show that FabH (and thus the FASII pathway) is not essential in an experiment that is not designed to be in low lipid conditions but rather in lipid rich conditions: Such high lipid conditions of culture in this study is granted by daily feedings with high fatty acid supplement (30-90 uM palmitic acid and 30-60 uM oleic acid). These fatty acid concentrations were used previously by Mitamura et al. (2005) and Miichi et al.(2007) to replace non-determined supplements such as Serum or Albumax supplement to grant similar growth by a completely controlled culture medium.

This means the concentrations above do not represent limited fatty acid concentrations, especially not with daily feeding (representing an excess supplied amount of lipids, unlike regular 48h feedings) that allowed the authors to easily reach very high non-physiological parasitaemia of more than 20%!! Amiar et al. previously showed essentiality of FabI in *P. falciparum* in the limited fatty acid culture at a lower concentration (<30uM 16:0, <45um 18:1), than the Mi-Ichi et al. controlled medium with regular 48 h culture feeding. Therefore, with the current experimental settings, the FAH KO is placed in high lipid conditions, thus preventing any conclusion on its essentiality under low lipid conditions.

The basis for the reviewer’s statements here is unclear, as this critique and the conditions it describes do not conform to the published conditions reported in the Amiar et al. 2020 paper. The methods section of that study for “*Plasmodium falciparum* growth assays” explicitly states (page e7):

“Media was replaced daily, sub-culturing were performed every 48 h when required, and parasitemia monitored by Giemsa-stained blood smears. Growth assays in lipid-depleted media were performed by synchronizing parasites before transferring trophozoites to lipid-depleted media as previously reported (Botte ´ et al., 2013; Shears et al., 2017). Briefly, lipid-rich AlbuMAX II was replaced by complementing culture media with an equivalent amount of fatty acid-free bovine serum albumin (Sigma), 30 µM palmitic acid (C16:0; Sigma) and 45 µM oleic acid (C18:1; Sigma).”

We used identical culture conditions to those described above: fatty acid-free BSA in place of lipid-rich AlbuMAX, 30 µM palmitic acid, and 45 µM oleic acid. We thus obtained growth results that contrast with the prior study and suggest that additional, future studies will be required to understand and resolve these differences.

Furthermore, it is too uncertain to conclude that ACP is only essential for the mevalonate pathway.

Please see our response above that clarifies our model for ACP function in supporting pyruvate kinase II and the many apicoplast pathways that appear to depend on PKII.

This would be a similar discussion to the Yeh et al. 2011 and the Swift et al., where induced Apicoplast knockout caused parasites to require IPP to survive, but there were always remnant apicoplast vesicles and thus the putative presence of an active FASII in the parasite, where de novo fatty acid synthesis could be maintained.

It is extremely unlikely that FASII remains active upon apicoplast disruption and loss of the apicoplast genome. The apicoplast-encoded SufB is lost upon apicoplast disruption and can no longer participate in making Fe-S clusters. Without Fe-S synthesis, the apicoplast lipoate synthase (LipA) cannot make lipoate to activate pyruvate dehydrogenase (E2 subunit) and produce the acetyl-CoA needed for FASII activity. There is no experimental evidence that FASII remains active upon apicoplast disruption and loss of the apicoplast genome.

Amiar et al. (2020) and Krishnan et al. (2020) showed that disruption of FASII and absence of de novo FA synthesis in *T. gondii* could be compensated by the exogenous supplementation of myristic acid, C14:0.

As explained above, we acknowledge that FASII contributes to *Toxoplasma gondii* growth and includes functions that appear to differ from *P. falciparum*.

Here, high fatty acid supplementation using commercially available fatty acids may include unexpected fatty acid species such as myristic acid in palmitic acid or oleic acid, since all commercially available fatty acids guarantee only >99% but not 100%. If *P. falciparum* requires a very, very low amount of myristic acid to survive, the amount of possible contamination, like 1 nM, may be sufficient to maintain their survival. Thus, ACP and FabH might be very important to generate de novo fatty acids within parasites, but this was not shown by the authors.

As noted above, we used identical culture conditions and commercial sources of defined fatty acids to those reported in the Amiar et al. study. We do not see a basis for the reviewer’s critique that the two studies utilized differing culture conditions. Nevertheless, we agree that future studies are needed to understand and resolve these differences.

Therefore, the manuscript currently contains incorrect conclusions on the potential essentiality/use of FASII, against current experimental evidence.

As explained above, we do not see a basis for the reviewer’s critique here or for viewing one study as more or less definitive than the other, as identical culture conditions were used yet contrasting results were obtained for reasons that remain uncertain. Future studies beyond the scope of the present manuscript will be required to fully understand and resolve these differences.

**Recommendations for the authors:**

**Reviewer #2 (Recommendations for the authors):**

We either request more solid experimental evidence showing the absence of fatty acid synthesis at low fatty acid conditions by re-doing the growth assay in the lower fatty acid feeding conditions without daily feeding to clarify if the ACP and FabH are essential in the blood stage growth, or not; as well as showing the absence of fatty acid synthesis at low fatty acid conditions using isotope labelled precursor. Without these, the authors cannot conclude on this important point. Alternatively, toning down the text to acknowledge the possibility of FASII being active and critical under certain conditions would be acceptable.

We note that the Amiar et. al 2020 study cited by the reviewer reported growth assays for WT and ∆FabI NF54 *P. falciparum* in low-lipid conditions that similarly lacked direct tests of FASII activity by isotope labeling.

We agree that our results, which utilized distinct ∆ACP and ∆FabD NF54 (PfMev) lines, contrast with the results and conclusions of the Amiar et al. study. We fully agree with the reviewer that future studies, utilizing tandem growth assays and isotope-labeling metabolic flux assays (e.g., mass spectrometry), will be required to fully test and understand the dependence of FASII activity on the lipid content of the growth medium and the functional dependence of *P. falciparum* growth on FASII activity in low-lipid conditions.

We have revised the final paragraph of the second results section of our manuscript to incorporate this perspective:

“These results contrast with the prior study [49] of ∆FabI parasites and the proposed model that blood-stage *P. falciparum* requires FASII activity for growth in low-lipid conditions and suggest that parasites can rely on scavenging host-derived fatty acids over a wide range of lipid conditions. Future studies involving tandem growth and isotope-labeling experiments of WT and ∆FASII parasites will be required to fully test and understand FASII function and the dependence of *P. falciparum* growth on this pathway in low-lipid conditions.”
